# The chemistry of bisallenes

**DOI:** 10.3762/bjoc.8.225

**Published:** 2012-11-15

**Authors:** Henning Hopf, Georgios Markopoulos

**Affiliations:** 1 Institute of Organic Chemistry, Technical University of Braunschweig, Hagenring 30, D-38106 Braunschweig, Germany, fax: +49-(0)531-391-5388

**Keywords:** alicyclic, bisallenes, cyclic, cycloadditions, cycloisomerization, isomerization, molecular complexity, step economy

## Abstract

This review describes the preparation, structural properties and the use of bisallenes in organic synthesis for the first time. All classes of compounds containing at least two allene moieties are considered, starting from simple conjugated bisallenes and ending with allenes in which the two cumulenic units are connected by complex polycyclic ring systems, heteroatoms and/or heteroatom-containing tethers. Preparatively the bisallenes are especially useful in isomerization and cycloaddition reactions of all kinds leading to the respective target molecules with high atom economy and often in high yield. Bisallenes are hence substrates for generating molecular complexity in a small number of steps (high step economy).

## Table of Contents

Introduction

Review

1. Acyclic conjugated bisallenes
  1.1 Synthesis of hydrocarbons
  1.2 Synthesis of functionalized systems
  1.3 Spectroscopic and structural properties of conjugated bisallenes
  1.4 The chemical behavior of conjugated bisallenes
  1.4.1 Pericyclic reactions
  1.4.2 Ionic reactions
  1.4.3 Metal-induced reactions
2. Acyclic nonconjugated bisallenes
  2.1 1,2,5,6-Heptatetraene and its derivatives
  2.2 1,2,6,7-Octatetraene and its derivatives
  2.3 Higher acyclic α,ω-bisallenes
  2.4 The chemical behavior of nonconjugated, acyclic α,ω-bisallenes
3. Bisallenes with unsaturated spacer elements
4. Bisallenes containing ring systems
  4.1 Aromatic bisallenes
  4.2 Bisallenes connected by cyclic spacer groups
  4.2.1 Semicyclic bisallenes
  4.2.2 Endocyclic bisallenes
  4.2.3 Exocyclic bisallenes
5. Bisallenes as reactive intermediates
6. Bisallenes with heteroatoms in their tethers (heteroorganic bisallenes)
  6.1 Thermally induced reactions of heteroorganic bisallenes
  6.2 Transition metal-induced reactions of heteroorganic bisallenes

Conclusion

Acknowledgements

References

## Introduction

**Figure 1:**
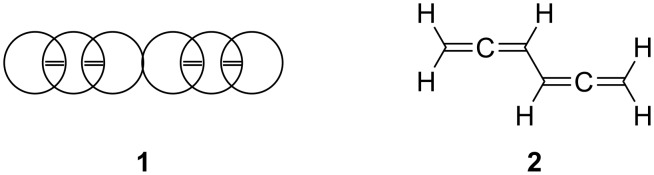
Loschmidt’s structure proposal for benzene (**1**) (Scheme 181 from [3]) and the corresponding modern structural formula **2**.

As every student of organic chemistry knows, the early years of this branch of science were exciting and confusing at the same time. This is particularly true for the chemistry of aromatic compounds before Kekulé finally established the hexagonal structure for benzene [1]. Benzene was discovered by Faraday in 1825, and Kekulé published his concept, which would revolutionize organic chemistry, in 1865 (and his dreams about the benzene ring in 1890) [2]. In fact, the early structure proposals for benzene varied considerably and Loschmidt, a contemporary of Kekulé who was also interested in developing a structural theory for organic compounds, wrote his first benzene structure (**1**) in 1861 as shown in Figure 1 [3–5]. If we translate this model into a modern structural formula we arrive at 1,2,4,5-hexatetraene (**2**, biallenyl) which, of course, also possesses the sum formula C_6_H_6_.

**Figure 2:**
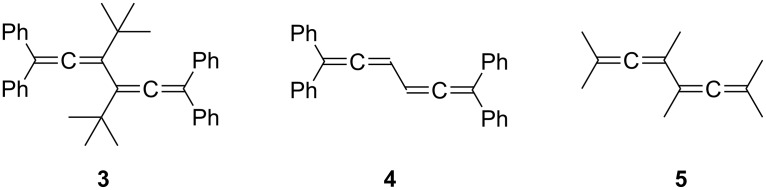
The first isolated bisallenes.

As it turned out, **2** was only prepared and characterized in 1970 [6], nearly 110 years after Loschmidt’s and Couper’s speculations. Still, there are some scattered reports on (conjugated) bisallenes in the chemical literature preceding the isolation of the parent system. The first “true” bisallene derivative was reported by Marvel and co-workers in the 1930s [7–8]; it is the highly substituted and hence stabilized hydrocarbon **3**, a hexa-substituted derivative of **2** (Figure 2).

Twenty-five years later, Kuhn and Fischer presented the preparation of 1,1,6,6-tetraphenyl-1,2,4,5-hexatetraene (**4**) as the first conjugated bisallene with unsubstituted allene carbon atoms [9]. And finally, also in the 1960s, Jacobs and Prempree reported on the synthesis of the first hexa-alkylated bisallene, hydrocarbon **5** [10–11]. We shall return to the preparation of these three compounds later.

The upsurge in interest in allene chemistry is a recent phenomenon. During the 1970s (the very few) German chemists working on and with allenes used to answer inquiries about their research interests with the (German) sentence, “*Ich arbeite (ueber) Allene*”, with allene being Berlin dialect for “alone”, i.e., “*I am working alone/on allenes*”. The international scene was not very much different: allenes and cumulenes were regarded by the chemical community as exotic compounds, of interest at best for the study of stereochemical problems (going back to the days of van’t Hoff), but not as substrates and/or intermediates useful in preparative organic chemistry. Today the situation has completely changed, as is, among other things, demonstrated by numerous review articles and a recent two volumes monograph on modern allene chemistry [12–14].

As far as the organization of the present review is concerned, we have tried to cover the literature up to the end of 2011. The emphasis will be on the synthesis and the chemical transformations of bisallenes. Often bisallenes are found as side products, formed in small amounts in otherwise nonrelated reactions; these cases will not be dealt with here explicitly. The scope will be restricted to bisallenes in the strict sense of the term, i.e. we shall not be concerned with tris- and higher oligoallenes, which have also been reported occasionally. Heteroanalogues of the bisallenes such as ketenes [15] or isothiocyanates [16] will not be dealt with either, even if they contain one “intact” allene group. Also excluded are all systems in which the role of one or more of the allene carbon atoms of the bisallene is taken over by metal atoms [17–19].

**Figure 3:**
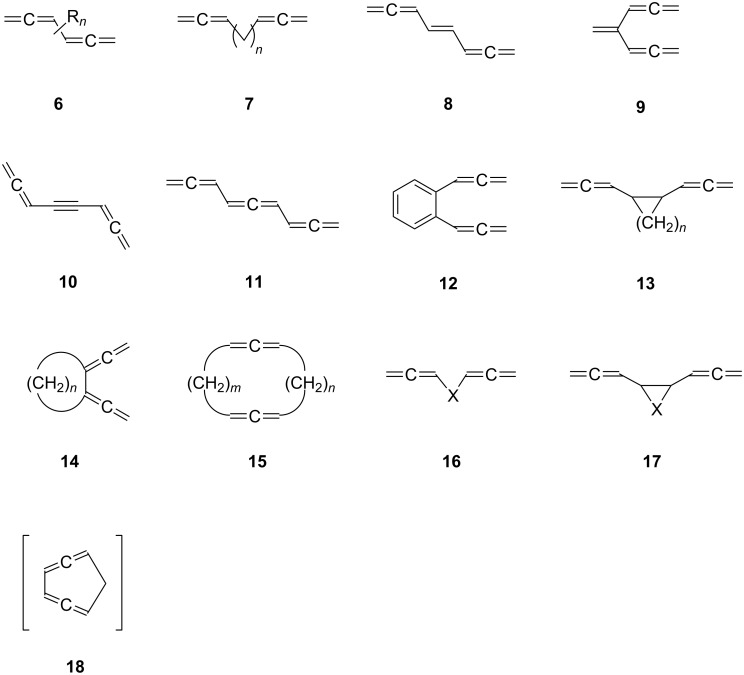
Carbon skeletons of selected bisallenes discussed in this review.

We will begin our discussion with the conjugated bisallenes **6** (R*_n_* with *n* from 0, i.e. H, up to 6, fully substituted, Figure 3), where the substituents R can vary from alkyl and aryl to any type of functional group. For practical reasons we will not use IUPAC-nomenclature for derivatives of **6**, but rather regard them as substituted conjugated bisallenes (1,2,4,5-hexatetraenes). In fact, we will see that bisallenes with “real” functional groups, especially substituents containing carbonyl groups, are comparatively rare.

In the next step, we “separate” the two allene moieties from each other. This can be accomplished in its simplest form by interspersing a methylene group between the two allene units (**7**, *n* = 1). Obviously, the larger *n* is, the bigger the distance between the terminating allene groups. However, from a certain point on, we would not expect any interaction between the two groups anymore, making, e.g., ring-closing processes increasingly unlikely (although the reader will be astonished to see how large *n* can be before this point is actually reached; see Section 2.3 below). Extremely long α,ω-bisallenes of this type should behave just like two (connected) monoallenes. Of course, the individual methylene groups in **7** may carry functional groups.

Next we introduce unsaturated spacers (“tethers”) between the two allene groups. The simplest interchange will involve a double bond, which can hold its two allene substituents in vicinal (structure **8**) or geminal form, **9**. Of course, a triple bond can serve as a spacer, **10**, as can another allene group, **11**, although we are then dealing with a trisallene [20].

An aromatic ring can play the tether role also, as demonstrated by the very simple example **12**, as well as any other mono-, **13**, or bi- and polycyclic ring system. In **13** the two allene moieties are both in exocyclic arrangement. However, both semicyclic, **14**, and endocyclic arrangements, **15**, are also conceivable. If one methylene group in **7** (*n* = 1) is replaced by a heteroatom, the heteroorganic bisallenes **16** result, in their simplest form as ethers (X = O), amines (X = NR), thioethers (X = S), etc. Analogously bisallenic epoxides (**17**, X = O) or aziridines (**17**, X = NR) can formally be generated from the corresponding all-carbon analogues **13**. Finally, we have summarized several interesting reactions in which bisallenes have been postulated as reaction intermediates; a structurally simple example to illustrate this group of compounds is 1,2,4,5-cycloheptatetraene (**18**), which is presumably generated as a reaction intermediate in a pyrolysis reaction (see Section 5).

How fast a very large structural variety can be generated from these basic patterns, becomes obvious if one realizes that, for example, in the aromatic systems **12**, the allene units can be anchored also in *meta*- and/or *para*-position and condensed aromatic compounds or heteroaromatic moieties may take over the role of the aromatic core.

## Review

### 1 Acyclic conjugated bisallenes

#### 1.1 Synthesis of hydrocarbons

Bisallenes of type **2** or **6** have been prepared by many different routes and strategies from which, however, three stand out:

i) The C_6_-carbon framework may be constructed by adding two more carbons to a C_4_-precursor. Most often this route takes the form of C_4_ + C_1_ + C_1_.

ii) The C_6_-system is built up by coupling two C_3_-units (C_3_ + C_3_). This is probably the most versatile way to prepare substituted, conjugated bisallenes.

iii) The starting material already contains six consecutive carbon atoms, but the system is either lacking the desired substitution pattern or the proper degree of unsaturation (C_6_ + 0).

A fourth possibility, the coupling of three C_2_ units (C_2_ + C_2_ + C_2_) is extremely rare and it appears that only two examples have been reported so far (see Scheme 12 and Scheme 17).

**Scheme 1:**
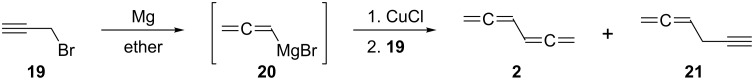
The preparation of 1,2,4,5-hexatetraene (**2**).

Starting with the simplest bisallene, the C_6_-isomer 1,2,4,5-hexatetraene (**2**), this parent system was first prepared by a protocol according to general route (ii) [6]. As summarized in Scheme 1, conversion of the C_3_-building block propargyl bromide (**19**) into its Grignard reagent, allenyl magnesium bromide (**20**), takes place first. The subsequent addition of CuCl very likely generates a copper organic intermediate, which is then coupled with a second equivalent of **19** to yield **2** and its isomer **21** (1,2-hexadien-5-yne, propargylallene). Formally, the dimerization of **19** to **2** involves two S_N_2’-reactions, but detailed mechanistic experiments have not been undertaken for this particular reaction so far [21]. The two C_6_H_6_-isomers **2** and **21** are produced in ca. 2:3 ratio and the total yield varies from 40–70%, depending primarily on the workup conditions. The separation of the two hydrocarbons by distillation is difficult because they are unstable und possess similar physical constants. If pure **21** were needed, **2** can be removed from the reaction mixture by trapping it by a Diels–Alder reaction (see Section 1.4.1 below). The analytically pure hydrocarbons were obtained by preparative gas chromatography [6]. Solutions of **2**/**21** in ether containing up to 50 g of the former can be prepared easily [21].

In principle this coupling reaction can also be employed to prepare the 1- and 3-monomethyl derivatives of **2** [22]. For preparative applications this approach is unsatisfactory, however, since too many isomers and secondary products are produced and their separation is difficult. Nevertheless, to determine the spectroscopic properties of these C_7_H_8_-hydrocarbons, they can be (and have been) separated by preparative gas chromatography.

**Scheme 2:**
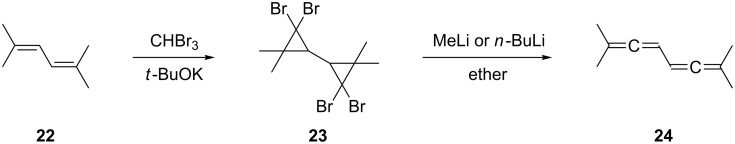
The preparation of a conjugated bisallene by the DMS-protocol.

In preparative chemistry one of the most frequently used conjugated bisallenes is the 1,1,6,6-tetramethyl derivative of **2**, **24** (Scheme 2), which was synthesized conveniently by Skattebøl and co-workers by a route that was later termed the Doering–Moore–Skattebøl synthesis of allenes (DMS-synthesis) [23], i.e., a C_4_ + C_1_ + C_1_-route belonging to category (i). In this synthesis the tetramethylbutadiene **22** is first dibromocyclopropanated to the tetrabromide **23**, which, on treatment with an organolithium reagent, is debrominated/rearranged to the target hydrocarbon **24**. Hydrocarbon **24** is a stable colorless solid that can be worked with under normal laboratory conditions without any problems.

**Scheme 3:**
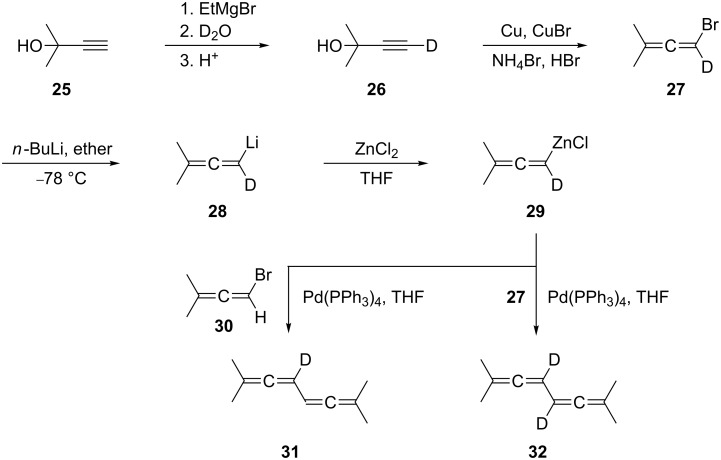
Preparation of the 3-deuterio- and 3,4-dideuterio derivatives of **24**.

The partially deuterated derivatives **31** and **32** have also been obtained by a C_3_-dimerization route (category (ii), see above), as shown in Scheme 3 [24]. 3,3-Dimethylpropargyl alcohol (**25**) was first converted by standard methodology to the deuterioallene **27** via the deuterioacetylene **26**. Metalation of **27** with *n*-butyllithium provided the allenyllithium reagent **28** next, which was subsequently converted into the organozinc reagent **29**. In a Negishi-type coupling of this intermediate with either the bromoallene **30** or its deuterated version **27** the two target compounds were obtained in the final step. Neither the 3-deuterio nor the 3,4-dideuterio derivative of the parent system **2** seem to be known.

Applying the DMS-approach to either isoprene or 1,3-pentadiene leads to the desired monomethyl derivatives of **2**, 3-methyl-1,2,4,5-hexatetraene and 1,2,4,5-heptatetraene, but the yields are poor and fulvenes are produced as side products [25]. The formation of fulvene derivatives and other compounds containing five-membered rings has often been noted in this type of approach [26]. These cyclic products are formed by mechanisms involving carbene (generated during the dehalogenation step of the dibromocyclopropane precursors) to carbene rearrangements followed by hydrogen shifts.

**Scheme 4:**
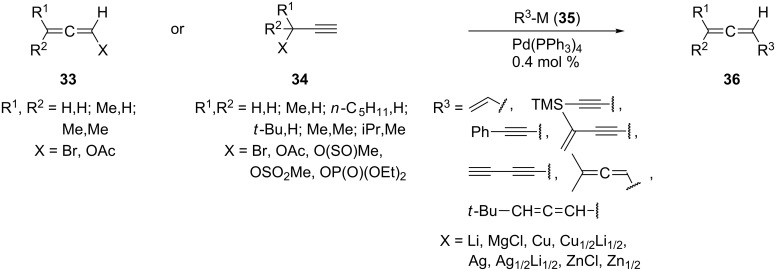
A versatile method to prepare alkylated conjugated bisallenes and other allenes.

The above C_3_-dimerization pathway (Scheme 3) via organozinc compounds goes back to Vermeer et al. and appears to be not only the most general route to alkylated conjugated bisallenes [27–31], but also to many other highly substituted allenes; it is summarized in compact form in Scheme 4. By this approach the substituted allene derivatives **33** or the propargyl derivatives **34** are coupled with organometallic reagents **35** in the presence of a Pd-catalyst to the different allenes and bisallenes **36**. The yields are normally good to excellent and the workup does not require chromatography of any kind.

**Scheme 5:**
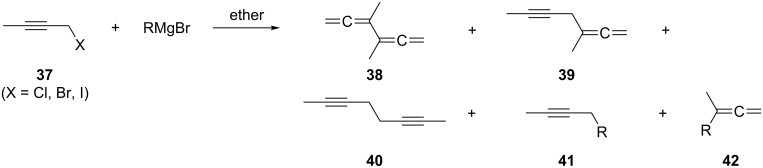
A preparation of 3,4-dimethyl-1,2,4,5-hexatetraene (**38**).

Many other (C_3_ + C_3_)-couplings have been reported in the chemical literature. For example, 2-butynyl halides **37** on treatment with Grignard reagents RMgBr provide hydrocarbons such as 3,4-dimethyl-1,2,4,5-hexatetraene (**38**), but the composition of the product mixture is anything but simple (Scheme 5) [32–36]. Not only are the three isomeric C_3_-coupling products **38** to **40** produced, but also those of **37** with the Grignard reagent, **41** and **42** (R = Et, *n*-Pr). All of these C–C-coupling reactions are thought to occur via radical intermediates. The potential of the coupling of 2-alkynylboranes with propargyl bromides to furnish alkylated bisallenes has evidently not been exploited yet [37].

**Scheme 6:**
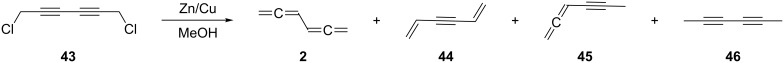
A (C_6_ + 0)-approach to 1,2,4,5-hexatetraene (**2**).

The last general approach (see (iii) above), in which the substrate molecule already contains all the carbon atoms required in the product, has also been employed for the preparation of the parent hydrocarbon **2** (Scheme 6). The reduction of 1,6-dichloro-2,4-butadiyne (**43**) in methanol with a zinc/copper couple furnished the bisallene **2**, but also provided at least three other C_6_H_6_-isomers, **44**–**46**, requiring extensive chromatographic separation and purification [38]. Biallenyl **2** is also produced when 1,5-hexadiyne is treated with sodium ethoxide in ethanol at 65 °C for 24 h. But again this is not a practical preparative method since it results in the formation of too many other isomers of the substrate and **2** [39].

**Scheme 7:**
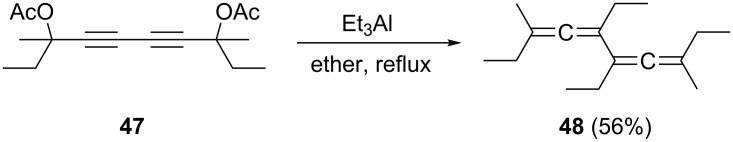
The preparation of a fully alkylated bisallenes from a 2,4-hexadiyne-1,6-diol diacetate.

Alkylated and terminally cycloalkylated bisallenes have also been obtained by another approach starting from C_6_-derived precursors: the alkylation of diacetylene glycol esters with organoaluminum reagents. The example shown in Scheme 7 is typical. Treatment of diacetate **47** with triethylaluminum in ether under reflux for 4 h provides the fully alkylated bisallene **48** in 56% yield [40–41].

**Scheme 8:**
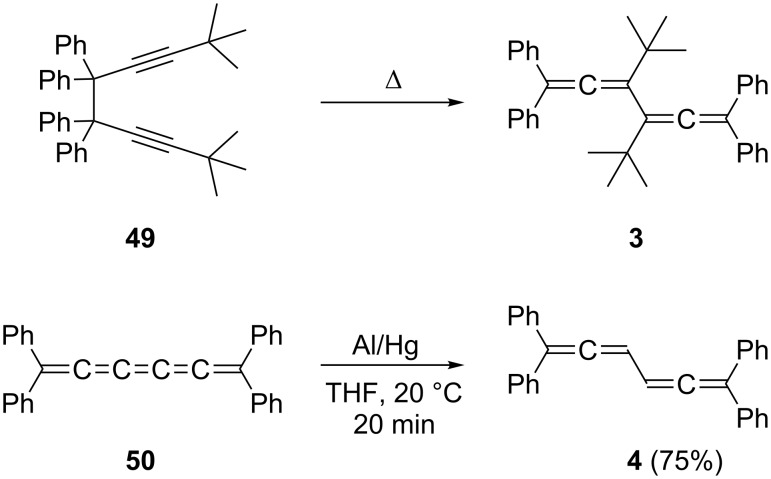
The preparation of the first phenyl-substituted conjugated bisallenes **3** and **4**.

Turning to phenyl- and/or aryl-substituted conjugated bisallenes, we have already encountered two classic representatives of this subclass: hydrocarbons **3** and **4** (see above). Both were obtained by general method (iii). In Marvel’s route to **3** [7–8] the bispropargyl precursor **49** was thermally isomerized to the bisallene (Scheme 8) by exploiting a process to which we shall return later (see Section 1.4.1). In Kuhn’s synthesis of **4** the [5]cumulene **50** was reduced in good yield to the conjugated bisallene by treatment with aluminum amalgam under very mild conditions [9].

**Scheme 9:**
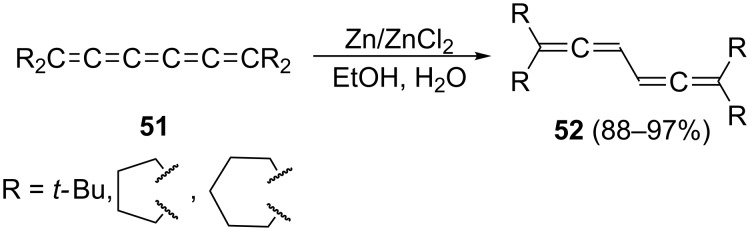
Selective hydrogenation of [5]cumulenes to conjugated bisallenes: another (C_6_ + 0)-route.

In an approach related to Kuhn’s reduction of **50** to **4**, tetraalkyl[5]cumulenes **51** are selectively hydrogenated with Zn/ZnCl_2_ in aqueous ethanol (Scheme 9) to the conjugated bisallenes **52**, again fully substituted in their terminal positions. The yields are almost quantitative (88–97%) and, provided the substrates **51** are available by a straightforward method, this synthesis can probably be extended [42].

**Scheme 10:**
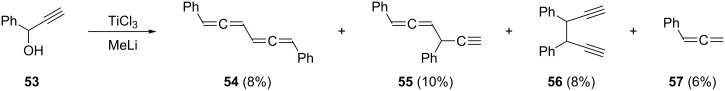
Aryl-substituted conjugated bisallenes by a (C_3_ + C_3_)-approach.

Not surprisingly the C_3_-dimerization protocol works for aryl-substituted bisallenes also as shown, for example, in Scheme 10 [43]. Thus, treatment of the phenylpropargyl alcohol **53** with TiCl_3_/CH_3_Li leads to the 1,6-diphenylbiallenyl **54**. Not only is the process characterized by a poor yield, but it also provides two isomers **55** and **56** of **54** as well as the deoxygenated (and rearranged) substrate **57**. Clearly, this is not a preparative method, but was sufficient to produce spectroscopic amounts of the desired hydrocarbon.

**Scheme 11:**
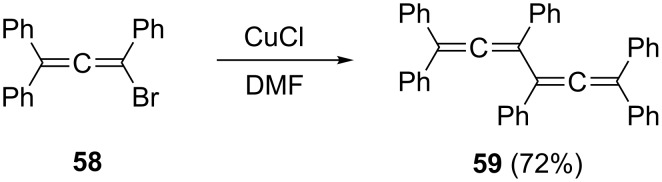
Hexaphenyl-1,2,4,5-hexatetraene (**59**) by a (C_3_ + C_3_)-approach.

Better results were obtained by Toda and Takehira, who dimerized the 1,1-diphenylallenyl bromide **58** with CuCl in DMF at room temperature in good yield to hexaphenylbisallene **59** (Scheme 11) [44]. The reaction could also be applied to the synthesis of other arylated bisallenes and presumably takes place via radical intermediates.

**Scheme 12:**
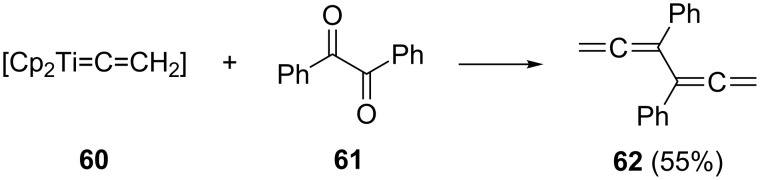
An allenation route to conjugated bisallenes.

A more recent way to allenes exploits the allenation of carbonyl compounds with titanocene alkenylidene reagents such as **60**. When applied to bibenzoyl (benzil), **61**, the 3,4-diphenylhexatetraene **62** is produced in good yield as the sole product (Scheme 12) [45]. Formally this constitutes a (C_2_ + C_2_ + C_2_)-route (see also Scheme 17).

We will encounter many of these routes to conjugated bisallenes in the coming sections dedicated to the preparation of compound types **7**–**17**. Not surprisingly, though, we will also meet new and specific approaches, and these will be discussed in due course.

#### 1.2 Synthesis of functionalized systems

Derivatives of **2** carrying “real”, i.e. heteroatom-containing functional groups, are comparatively rare and this constitutes one serious drawback of using these electron-rich compounds in preparative organic chemistry. For example, a systematic substructure search for conjugated bisallenes has revealed that virtually nothing is known about tetraolefins of this type carrying any carbonyl-containing function.

**Scheme 13:**
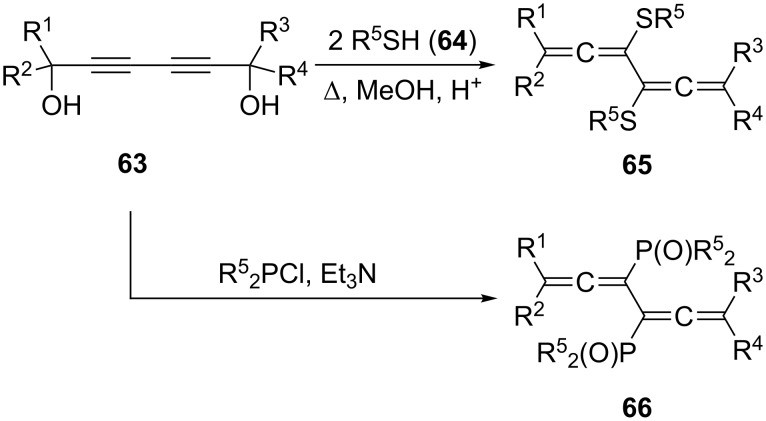
The preparation of 3,4-difunctionalized conjugated bisallenes.

As far as halogen substituents are concerned the situation is slightly better (see below). An early reference to the preparation of 3,4-dichloro-1,2,4,5-hexatetraene by Carothers and Coffman requires reinvestigation with modern spectroscopic methods [46–47]. If confirmed, it would not only describe the first preparation of a functionalized bisallene, but also the first representative of this class of molecules at all. Furthermore, by employing this dichloride in modern, metal-mediated coupling reactions it could become a useful substrate for the synthesis of highly unsaturated hydrocarbons, in particular of cross-conjugated systems (dendralenes) [48]. In fact, the only reaction coming close to a general route to introduce functionality into bisallenes at the present time seems to be the double S_N_2'-reaction of 2,4-hexadiyne-1,6-diol (**63**, e.g. R^1^ to R^4^ = aryl) and its derivatives with various nucleophiles (Scheme 13) [49–50]. Thus, heating the diols **63** with an aromatic or heteroaromatic thiol **64** provides the sulfur-substituted bisallene derivative **65** in good yields. Likewise, the stable diallenes **66** carrying phosphorus-containing substituents were obtained by heating the diols **63** with R^5^_2_PCl [51–54].

**Scheme 14:**
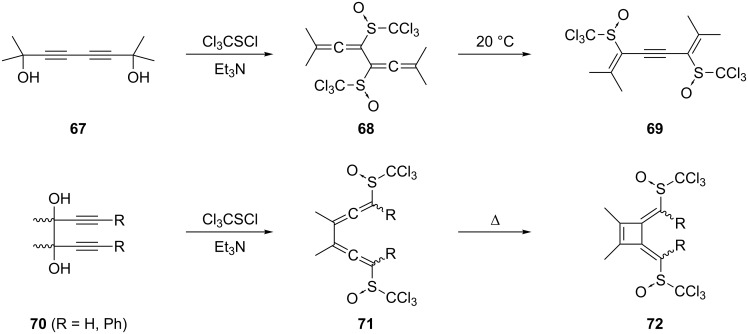
Problems during the preparation of sulfur-substituted conjugated bisallenes.

How critically the substituents in the 3- and 4-position determine the stability and reactivity of conjugated bisallenes was shown by treating the tetramethyl derivative **67** and related compounds with Cl_3_CSCl in triethylamine (Scheme 14) [55]. Although the bisallenes **68** could be identified unequivocally by spectroscopic methods at low temperatures, isolation of these compounds at room temperature failed because they easily isomerized to the dienynes **69**. The derivatives **71** can also be obtained from the isomeric diols **70** under comparable conditions, as shown by Braverman and co-workers. However, in only one case could these bisallene derivatives actually be isolated; all other compounds isomerized to the 3,4-bismethylenecyclobutene derivatives **72** by an electrocyclic process to which we shall return later (Section 1.4.1) [56–57].

**Scheme 15:**
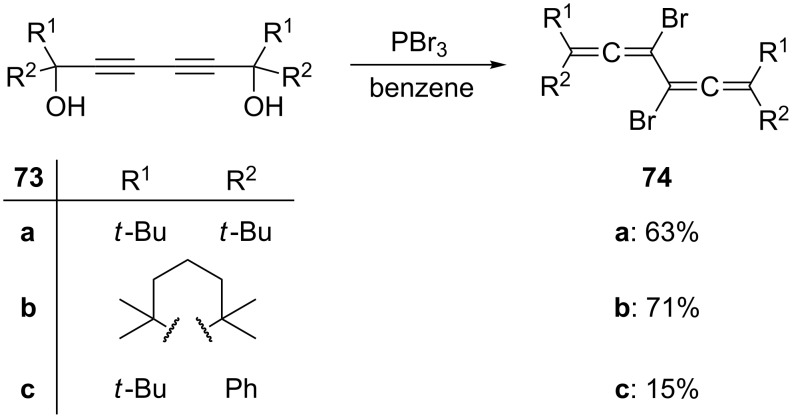
The preparation of 3,4-dibromo bisallenes.

In fact all of these reactions were preceded by the work of Bohlmann and Kieslich, who in the 1950s already prepared various 3,4-dibromobisallenes **74** by treating the highly substituted diyne diols **73** with phosphorus tribromide in benzene (Scheme 15) [58–63]. The details of these often overlooked seminal publications were recently studied by Parkhurst and Swager. According to these authors the 1,6-dibromide is produced first from the 1,6-diynediol. It isomerizes to the 3,4-dibromobisallene derivative next, especially under the influence of CuBr. The bisallene can subsequently undergo a CuBr-induced cyclization to a 1,2-dibromo-3,4-bismethylenecyclobutene derivative (see Section 1.4.1), thus providing a convenient route to these preparatively interesting four-membered ring compounds [64].

**Scheme 16:**
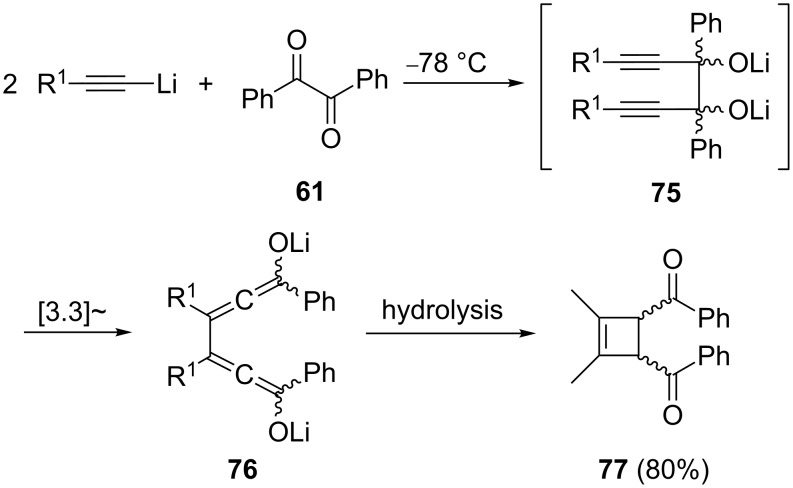
Generation of allenolates by an oxy-Cope rearrangement.

When an allene carries a hydroxy function, it is, of course, a special type of an enol (an allenol) and as such unstable. Yet, it may be generated as an intermediate to be employed in further transformations. Indeed, when α-diketones such as benzil (**61**, 1,2-diphenyl-1,2-ethanedione), are treated with two equivalents of a metal acetylide, the bisalcoholate **75** is produced first (Scheme 16) [65]. The expected [3.3]sigmatropic rearrangement of **75** takes place readily (see below, Section 1.4.1) at room temperature (it is, in essence, a double oxy-Cope rearrangement), and the resulting **76** subsequently undergoes an electrocylic ring closure to a four-membered ring system. On workup (hydrolysis) a mixture of the *cis* and *trans* isomers of **77** is obtained in very good yield (ca. 80%).

**Scheme 17:**
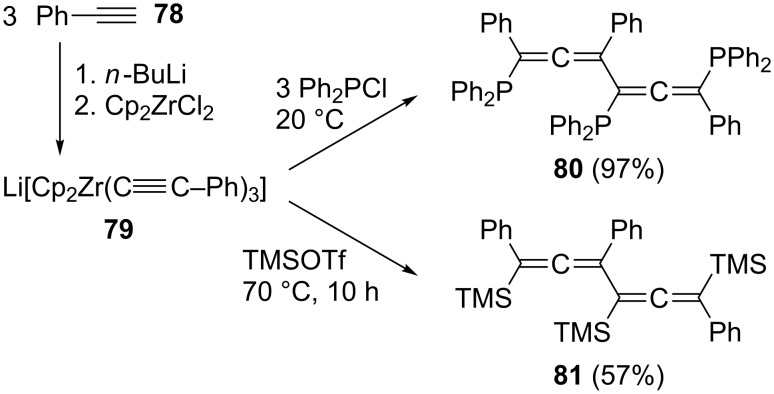
A linear trimerization of alkynes to conjugated bisallenes: a (C_2_ + C_2_ + C_2_)-protocol.

A conjugated bisallene carrying three diphenylphosphine substituents has been prepared recently by a Zr-promoted linear coupling of different aryl and hetaryl acetylenes in high yield (Scheme 17) [66]. Treating, e.g., phenylacetylene (**78**) first with *n*-butyllithium and subsequently with bis(cyclopentadienyl)zirconium dichloride generates the trimeric metal complex **79**, which on quenching with diphenylchlorophosphine yields the conjugated bisallene **80** in practically quantitative yield. Analogously, on quenching with trimethylsilyl triflate, **79** furnishes the tris(trimethylsilyl) derivative **81**. This formal (C_2_ + C_2_ + C_2_)-approach constitutes the only direct example we could find in the chemical literature for building up a bisallene derivative by a trimerization protocol from the same C_2_-precursor (acetylene).

**Scheme 18:**
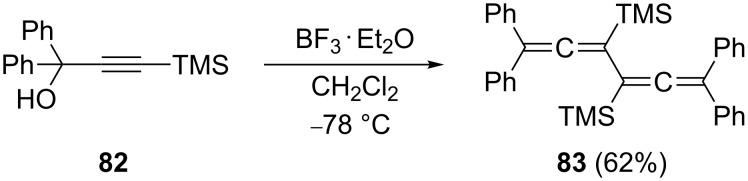
Preparation of a TMS-substituted conjugated bisallene by a C_3_-dimerization route.

Many other silylated derivatives of **2** have been described. For example, derivative **83** can be obtained in good yield by a C_3_-coupling route, as demonstrated in Scheme 18, from a trimethylsilyl-protected propargyl alcohol, **82** [67].

**Scheme 19:**
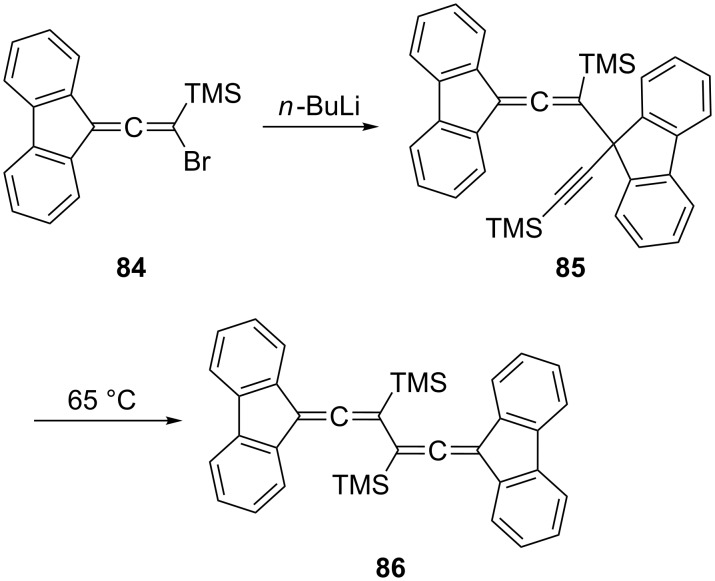
A bis(trimethylsilyl)bisallene by a C_3_-coupling protocol.

The fully substituted bisallene with two internal trimethylsilyl substituents **86** was prepared by McGlinchey et al. by a (2 × C_3_)-coupling route from the bromoallene **84** (Scheme 19) [68]. When **84** was heated with *n*-butyllithium in THF it dimerized to the unusual propargylallene derivative **85**, which, on slight heating (65 °C), isomerized to the bisallene **86**, which exists in the shown *transoid* conformation. On heating this conjugated bisallene to 110 °C it undergoes a remarkable isomerization/dimerization reaction to which we shall return in Section 1.4.1.

**Scheme 20:**
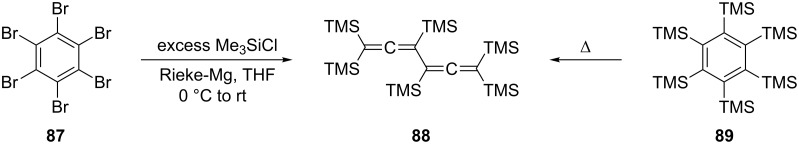
The rearrangement of highly substituted benzene derivatives into their conjugated bisallenic isomers.

The fully trimethylsilylated bisallene **88** is produced when hexabromide **87** is treated with excess trimethylsilyl chloride and Rieke magnesium at 0 °C (Scheme 20) [69–70]. The yield is low, however, and several other isomers of **88** are present in the reaction mixture. A far superior route to **88** consists of the thermal isomerization of hexakis(trimethylsilyl)benzene (**89**) [71–72]. Under flash vacuum pyrolysis conditions (400 °C, <0.01 Tor) **88** is produced from **89** as a mixture with several of its isomers (see below), but heating of the aromatic substrate at 200 °C for 10 h provides the bisallene as the sole product.

**Scheme 21:**
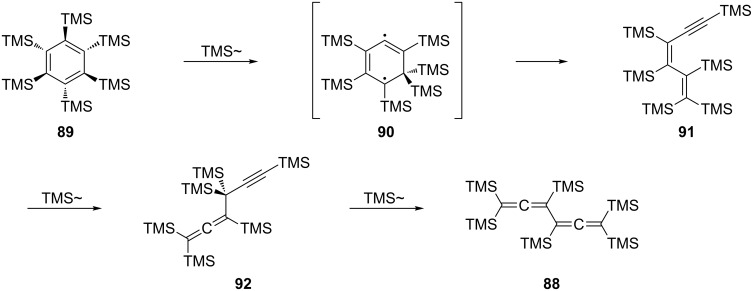
From fully substituted benzene derivatives to fully substituted bisallenes.

To rationalize the deep-seated reshuffling of the six carbon atoms of the benzene ring, Sakurai and co-workers suggested the mechanism given in Scheme 21. In the first step, the starting material **89**, which possesses a distorted chair structure, undergoes a TMS-shift to provide the diradical intermediate **90**. In the next step the ring is split to furnish derivative **91**, which, by another TMS-shift, isomerizes to the propargylallene **92**. The sequence ends with still another TMS-relocation to provide the final product **88**. Both, **91** and **92** are pyrolysis products under flash vacuum conditions (see above).

**Scheme 22:**
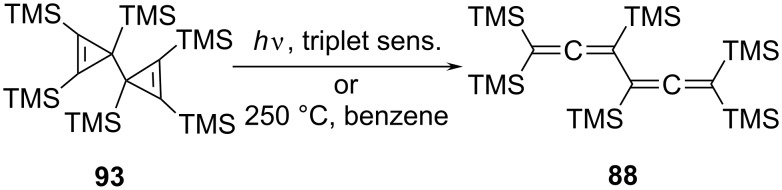
From a bicyclopropenyl to a conjugated bisallene derivative.

Interestingly, there are several other observations demonstrating that an extended network of relations exists between many of these C_6_-carbon frameworks. For example, hexakis(trimethylsilyl)-3,3'-bicyclopropenyl (**93**), upon either sensitized irradiation with a mercury-arc lamp or heating (250 °C, benzene), is converted quantitatively to **88**, which is presumably the thermodynamically most stable of the C_6_(TMS)_6_-isomers (Scheme 22) [73]. West and Priesner obtained **88** as one of the products produced when 2,4-hexadiyne was first metalated by the *n*-butyllithium/TMEDA-complex at room temperature and the resulting trilithiated compound MeC_5_Li_3_ subsequently trapped with trimethylsilyl chloride [74]. Comparable studies had already been carried out by Klein and Becker several years earlier [75].

**Scheme 23:**
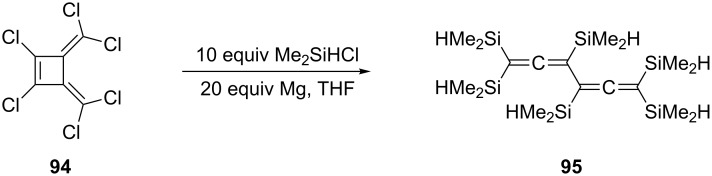
The conversion of a bismethylenecyclobutene into a conjugated bisallene.

Still another isomer of benzene is 3,4-bismethylenecyclobutene. When its hexachloro derivative **94** was treated by Gilman and co-workers with excess dimethylsilyl chloride and magnesium in THF, the hexasilylated derivative **95** was produced and again a ring-opening reaction to a conjugated bisallene had taken place (Scheme 23) [70]. In closing this section on silicon-substituted conjugated bisallenes, it should be mentioned that both the hexakis(trichlorosilyl) [76] and the hexakis(trimethylgermyl) [77] analogues of **88** and **95** are known.

**Scheme 24:**
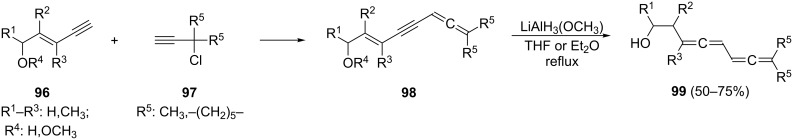
The preparation of monofunctionalized bisallenes.

The number of mono- and difunctionalized derivatives of **2** is still rather small. A case in point was provided by Goré and co-workers who obtained the primary and secondary alcohols **99** by first coupling the enynol **96** with different propargyl chlorides **97** to the allenynols **98** (Scheme 24) [78–79]. These latter derivatives were then reduced by either LiAlH_4_ or LiAlH_3_(OCH_3_) to provide the substituted bisallenes **99** in yields between 50 and 75%.

**Scheme 25:**
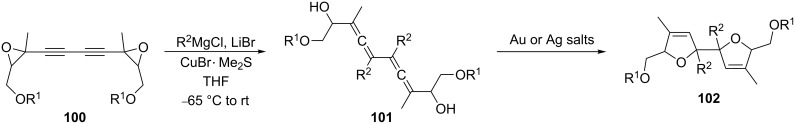
Preparation of bisallene diols and their cyclization to dihydrofurans.

Structurally comparable bisallene diols could be prepared, as shown by Krause and Poonoth [80–81], from bisoxiranes **100** by opening these with Grignard reagents in the presence of copper salts; this is again a double S_N_2'-process (Scheme 25). The resulting derivatives **101** are useful precursors for a double cyclization to novel bis(2,5-dihydrofuran) derivatives **102**; the yields in all three steps are acceptable to good (up to 60%).

**Scheme 26:**
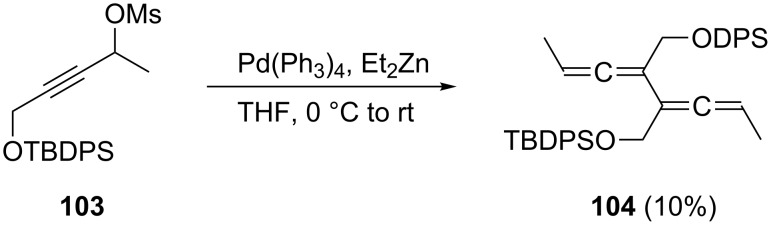
A 3,4-difunctionalized conjugated bisallene by a C_3_-coupling process.

A protected biallenyl diol, the derivative **104**, is produced as a mixture of diastereomers in small amounts (10%) when the mesylate **103** is treated with Pd(PPh_3_)_4_ and diethyl zinc in THF at 0 °C (Scheme 26; OTBDPS = *tert*-butyldiphenylsilyloxy) [82]. The allenic dimer is presumably formed by coupling of a transient allenyl zinc intermediate with an initially generated allenyl palladium species.

**Scheme 27:**
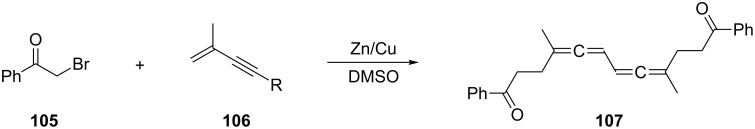
Preparation of a bisallenic diketone by a coupling reaction.

Another type of a difunctionalized conjugated bisallene has been produced by a coupling reaction. In this case the diketone **107** was obtained by a Zn/Cu-couple-induced coupling reaction between excess phenacyl bromide (**105**) and the enyne **106** (Scheme 27) [83]. Besides **107** its diyne and propargylallene dimers were produced, requiring a chromatographic separation of the product mixture. The overall yields were very good (78–91%); the relative yields of the three dimers were not determined, however.

**Scheme 28:**
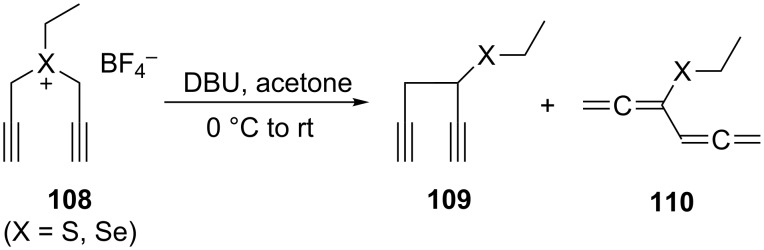
Sulfur and selenium-substituted bisallenes by a [2.3]sigmatropic rearrangement.

A monosubstituted bisallene has been prepared by Braverman and co-workers by treatment of the sulfonium or selenium salt **108** with DBU in acetone at 0 °C, providing a mixture of **109** and **110** in a 7:3 ratio (Scheme 28) [84]. For the formation of **110** a [2.3]sigmatropic rearrangement of **108** has been suggested, with the intermediately generated propargylallene derivative being stabilized by a prototropic shift to the final product **110**.

**Scheme 29:**
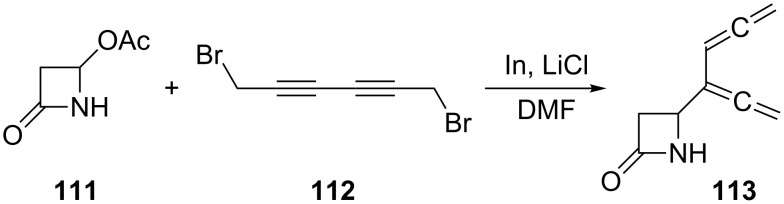
The biallenylation of azetidinones.

Another route to an unusually functionalized conjugated bisallene was reported recently [85]. The reaction amounts to a 1,2,4,5-hexatetraenylation of 4-acetoxy-2-azetidinones, such as **111** (Scheme 29). In this case the organoindium reagent, formed in situ from 1,6-dibromo-2,4-hexadiyne (**112**) and indium, was coupled with **111** in the presence of LiCl in DMF to yield the 2-azetidinone **113** carrying the 1,2,4,5-hexatetraen-3-yl substituent in the 4-position. The bisallene is a very good diene for Diels–Alder cycloadditions as will be discussed in Section 1.4.1.

**Scheme 30:**
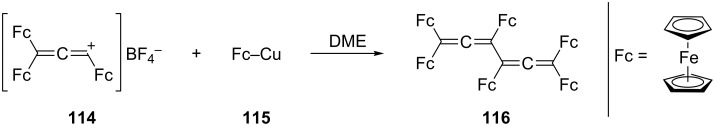
The preparation of a fully ferrocenylated conjugated bisallene.

The unusual hexakis(ferrocenyl)-1,2,4,5-hexatetraene (**116**) is produced as one of many side-products when tris(ferrocenyl)allenylium tetrafluoroborate (**114**) is treated with 1-cuprioferrocene (**115**) (Scheme 30) [86].

**Scheme 31:**
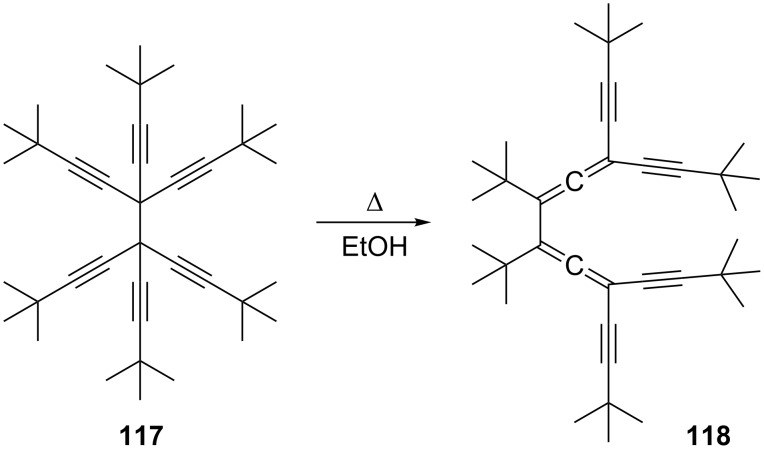
The first isomerization of a 1,5-hexadiyne to a 1,2,4,5-hexatetraene.

A number of bisallenic systems have been described in which the conjugated framework carries acetylenic substituents. In fact the earliest reports on these compounds, from Marvel and co-workers [87], go back to the 1930s (Scheme 31). Again, a thermal isomerization of a 1,5-hexadiyne derivative to a conjugated bisallene was employed, a process that will be discussed in detail in Section 1.4.1. Hexakis(*tert*-butylethynyl)ethane (**117**), when heated in alcoholic solution, isomerizes to the diallene **118**.

**Scheme 32:**
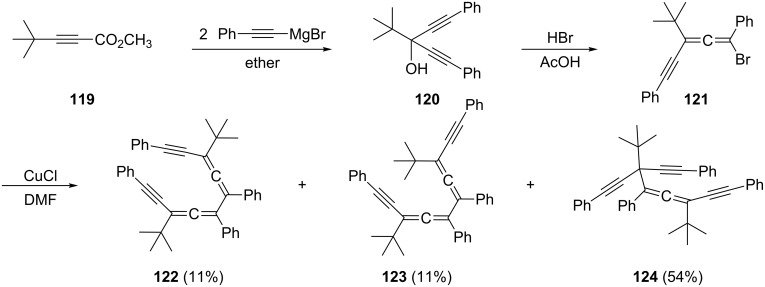
The preparation of alkynyl-substituted bisallenes by a C_3_-dimerization protocol.

Several hydrocarbon systems with a smaller number of triple bonds than **118** have also been reported. For example, Toda and Takahara have prepared the allenyne bromide **121** from the ester **119** via **120** by routine transformations (Scheme 32) [88–89]. When **121** is oxidatively coupled, e.g. a (C_3_ + C_3_)-approach to bisallenes is employed (see above), three dimers are obtained: the *d*,*l*-bisallene **122**, the *meso*-diastereomer **123** and the propargylallene **124**. We will discuss the remarkable thermal behavior of **122** and **123** in Section 1.4.1. A bisallene derivative carrying two alkynyl substituents at its inner (C-3 and C-4) positions is presumably formed when 1,1,2,2-tetrakis(*tert*-butylethynyl)ethane is first treated with phenyllithium and the resulting dianion is subsequently quenched with water [90].

**Scheme 33:**
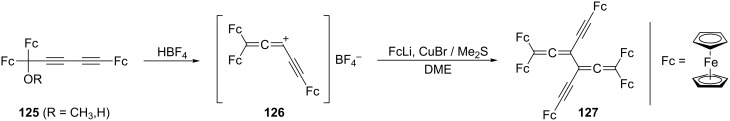
Preparation of another completely ferrocenylated bisallene.

Finally, it has been suggested that the diacetylenic bisallene **127**, carrying six ferrocenyl substituents (see Scheme 30) is produced when the diacetylene **125** is first ionized with HBF_4_ and the thus generated cation **126** reductively dimerized to **127** by treatment with FcLi and CuBr/Me_2_S in DME (Scheme 33) [91].

#### 1.3 Spectroscopic and structural properties of conjugated bisallenes

None of the numerous conjugated bisallenes discussed in Section 1.1 have been investigated from the structural and spectroscopic viewpoint as carefully as the parent system 1,2,4,5-hexatetraene (**2**). Not only have its vibrational spectra been measured and interpreted [92–93], but its most stable conformation has also been determined by electron diffraction in the vapor phase [94]. The results obtained by both of these physical methods are consistent with *C*_2_*_h_*-symmetry of the molecule, i.e. the *transoid* conformation is the most stable one. From its ^1^H NMR spectrum [95], measured in a nematic solvent at room temperature, it has also been concluded that the hydrocarbon prefers a planar *transoid* structure with *C*_2_*_h_*-symmetry, the terminal CH_2_-groups being orthogonal to the plane passing through the other atoms. Furthermore, the photoelectron spectra of **2** (and several of its isomers) as well as of some simple alkyl derivatives have been studied [96–97], as has been that of the tetramethyl derivative **24** [98–99].

Although **2** is a liquid at room temperature and its structure in the solid state has not been determined so far, many X-ray structural investigations of single crystals of derivatives of **2** have been carried out. We will present those performed on acyclic bisallenes in this Section and will return to the structures of various cyclic bisallenes in Section 4.2. As revealed by a structure search in the Cambridge Crystallographic Data files [100] about 42 bisallenes have presently been studied by X-ray structural analyses. Out of these, 21 have been carried out on acyclic bisallenes, including their *d*,*l* and *meso*-forms. Table 1 summarizes the structures of these derivatives and gives the leading references, which the reader can consult for further information.

**Table 1:** X-ray crystallographic studies on acyclic conjugated bisallenes of type **2**.

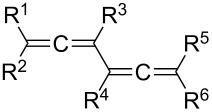						

R^1^	R^2^	R^3^	R^4^	R^5^	R^6^	ref.

CH_3_	CH_3_	H	H	CH_3_	CH_3_	[101]
*t*-Bu	CH_3_	H	H	CH_3_	*t*-Bu	[102]
CH_3_	CH_3_	TMS	TMS	CH_3_	CH_3_	[103]
Ph	CH_3_	H	H	CH_3_	Ph	[104]
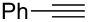	*t*-Bu	Ph	Ph	*t*-Bu	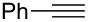	[88–89,105]
Ph	*p*-Tol	Br	Br	*p*-Tol	Ph	[106]
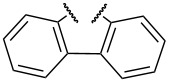		TMS	TMS	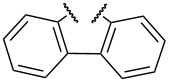		[68]
CH_3_	CH_3_	SO_2_CCl_3_	SO_2_CCl_3_	CH_3_	CH_3_	[57]
SiCl_3_	SiCl_3_	SiCl_3_	SiCl_3_	SiCl_3_	SiCl_3_	[77]
P(O)Ph_2_	Ph	P(O)Ph_2_	Ph	Ph	P(O)Ph_2_	[66]
MeOCH_2_-CH(OH)	CH_3_	*t*-Bu	*t*-Bu	CH_3_	MeOCH_2_-CH(OH)	[80–81,107]

#### 1.4 The chemical behavior of conjugated bisallenes

**1.4.1 Pericyclic reactions**

That **2** and its derivatives may be of interest for the study of pericyclic reactions becomes obvious when one looks at the electronic structure of these molecules. First of all there is a high concentration of π-electron density in a relatively small space: eight π-electrons distributed over six carbon atoms. This is always a good prerequisite for (enhanced) chemical reactivity, especially when one is dealing with energy-rich substrates as in the case of the biallenyls. Furthermore, the particular arrangement of double bonds in **2** leads to two types of π-electron (sub)systems: “internally” **2** possesses a conjugated diene system and “externally” it displays two cumulated ones. For the case of cycloadditions (see below) this should allow for [2 + 4] (Diels–Alder) and [2 + 2]cycloadditions (additions to allenes, ketenes, etc.). As we shall see later, both modes of cycloadditions have actually been observed.

**Scheme 34:**
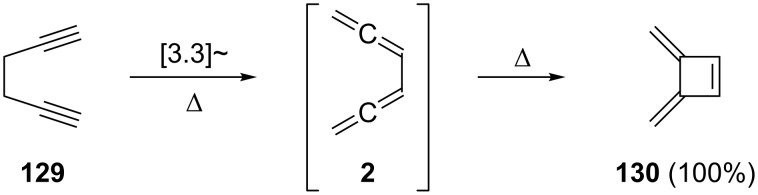
The cyclization of 1,5-hexadiyne (**129**) to 3,4-bismethylenecyclobutene (**130**) via 1,2,4,5-hexatetraene (**2**) as a reaction intermediate.

Our discussion on pericyclic reactions of **2** will start with thermal processes, i.e. isomerizations that are induced by heating this compound and its derivatives under various conditions. As already pointed out previously, this is also one of the oldest reactions known for conjugated bisallenes. After the first isolation of **2** it was quickly demonstrated that on heating (either in solution or in the gas phase) it cyclizes to 3,4-bismethylenecyclobutene (**130**) in quantitative yield (Scheme 34) [6]. Up to the present day this electrocyclization remains the most thoroughly investigated reaction of the conjugated bisallenes. As a matter of fact, these studies also demonstrate that **2** is the most likely intermediate in the thermal interconversion of 1,5-hexadiyne (**129**) to **130**, a Cope-type rearrangement that was discovered and discussed thoroughly by Huntsman and co-workers [108–111]. A shock-tube pyrolysis study of **2** in the temperature range of 540 to 1180 K at two nominal pressures (22 and 40 kbar) revealed that **2** plays an important role in the so-called propargyl mechanism, which is of importance in relation to the formation of aromatic compounds from nonaromatic precursors [112]. A radical cation Cope rearrangement of 1,5-hexadiyne (**129**) to the 1,2,4,5-hexatetraene radical cation was initiated by oxidizing bipropargyl radiolytically in a Freon matrix at 77 K [113]. The **129**→**2**→**130** sequence, and in particular its last step, has also been used several times for the preparation of functionalized bismethylenecyclobutenes, as demonstrated, for example, in Scheme 14.

If we look at the very early studies of Marvel and co-workers (see above, Introduction and Section 1.1), we can then rationalize the rearrangement of, e.g. **49** into **3** (Scheme 8, Section 1.1). The thermochemical parameters of the **2**→**130** cyclization have been determined and the activation parameters (*E*_a_ = 29.3 kcal/mol, log *A* = 13.6) [114–115] clearly speak for a concerted pericyclic process. This is, indeed, corroborated by studying the stereochemistry of the process, the work having been carried out by Huntsman [108–109], Pasto [29,115–116], Skattebøl [117] and others [118].

**Scheme 35:**
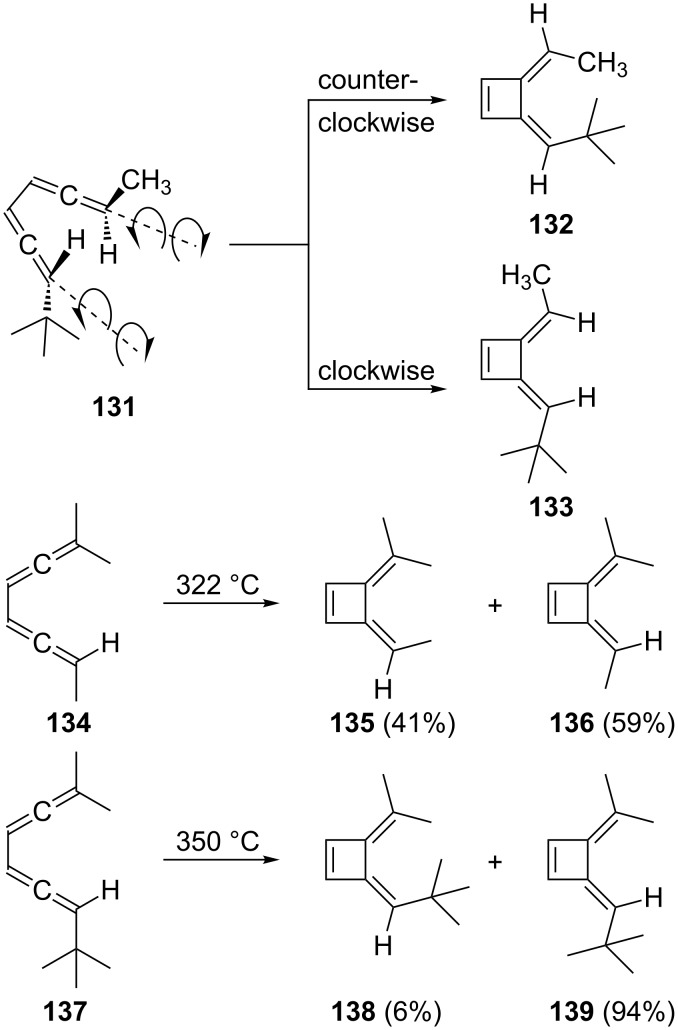
Stereochemistry of the thermal cyclization of bisallenes to bismethylenecyclobutenes.

As shown in Scheme 35, the electrocyclization occurs in a strictly conrotatory manner and the stereoselectivity of the process depends on the steric size of the terminal substituents. For example, bisallene **131** has two distinct conrotatory modes for ring closure: counterclockwise and clockwise, providing the two bismethylenecyclobutenes **132** and **133**, respectively. For derivative **134** the two conrotatory routes lead to **135** and **136** with a slight preference for the presumably sterically less hindered bismethylenecyclobutene **136**. In **137** with its highly space-filling *tert*-butyl substituent this preference for the “outside” arrangement of the voluminous substituent is clearly more pronounced (vastly preferred formation of **139** over **138**).

**Scheme 36:**
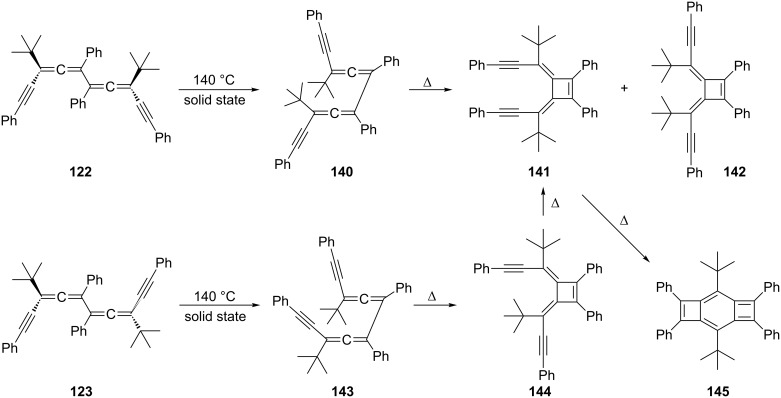
Bisallene→bismethylenecyclobutene ring closures in the solid state.

In a remarkable series of investigations Toda and co-workers have investigated the bisallene→bismethylenecyclobutene isomerization of the dialkynylated *d*,*l*- and *meso*-bisallenes, **122** and **123** respectively, in the solid state (Scheme 36) [119]. These rearrangements occur in high yield and with high stereoselectivity.

When **122** was heated in benzene it isomerized to **145** in quantitative yield within 2 h. A thorough analysis of the transformation, including its study in the solid state [105], revealed that it begins with a rotation around the central single bond, thus converting the *transoid* structure into the *cisoid* conformer **140**. Conrotatory ring closure subsequently leads to the bismethylenecyclobutene **141**. The alternative, which would provide isomer **142** with two *endo*-pointing *tert*-butyl groups, is avoided. In **141** the two phenethynyl moieties are so close that the hydrocarbon readily cyclizes to **145**. The situation for *meso*-compound **123** is similar. After the initial conformational change to **143** ring closure provides the *in*,*out*-isomer **144**, which cyclizes to **145** via the same intermediate **141**. The overall process is not only of interest because it involves transformations from single crystal to single crystal but also because of the benzocyclobutadiene derivatives **145**, which are obtained in unprecedented ease and display anomalies in their bond lengths (C_sp_^2^–C_sp_^2^ bonds as long as 1.540 Å) [120]. For another route to benzocyclobutenes see Section 2.4, Scheme 71.

**Scheme 37:**
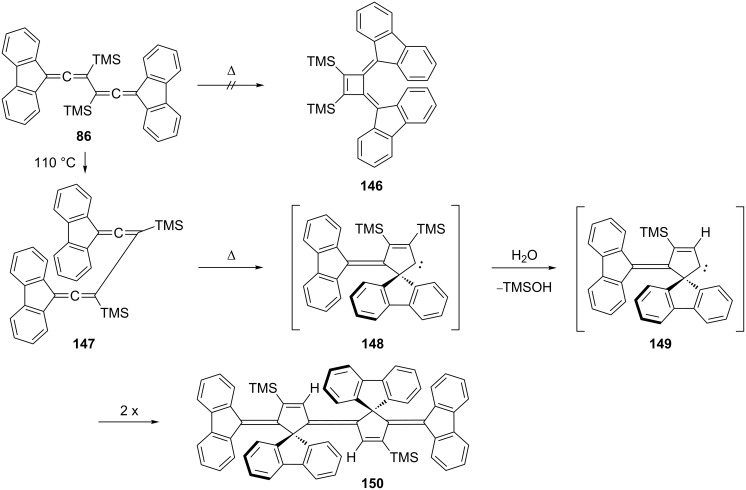
A bisallene cyclization/dimerization reaction.

A most interesting electrocyclization/dimerization process has been discovered by McGlinchey and co-workers for the disilylated hydrocarbon **86** (Scheme 37; see also Scheme 19, Section 1.2) [68]. Heating the highly hindered conjugated bisallene **86** in toluene does not lead to the expected bismethylenecyclobutene **146**; instead the isomerization takes a completely different course. This time no monomeric but a dimeric product is formed, i.e. the unusually structured TMS-derivative **150**. To rationalize this surprising result, the authors propose that after the conformational change of **86** to **147**, formation of the five-membered carbene **148** occurs, which by loss of one TMS-substituent is transformed into the carbene **149**. And this ultimately dimerizes to **150**. Derivative **150** can be desilylated to a C_60_H_36_ hydrocarbon, whose C_36_ backbone represents ca. 60% of the fullerene framework and which can be mapped onto C_60_ [121].

**Scheme 38:**
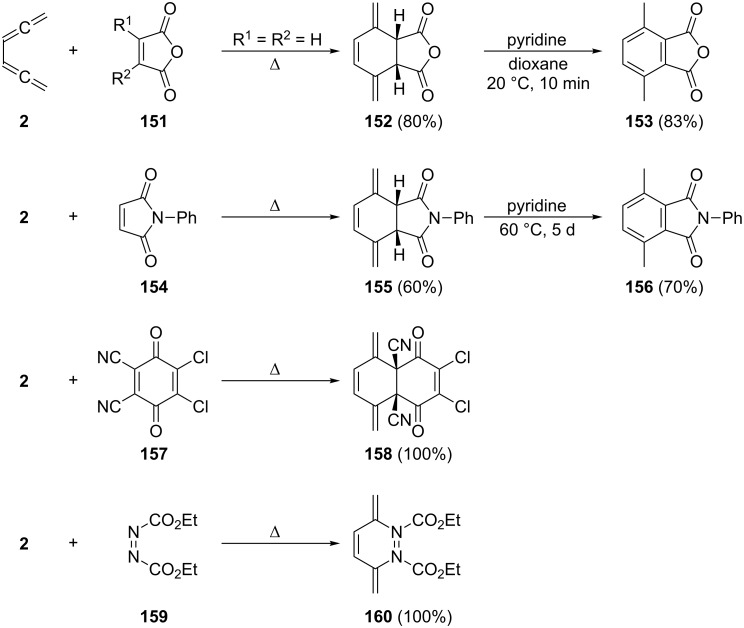
A selection of Diels–Alder additions of 1,2,4,5-hexatetraene with various double-bond dienophiles.

A pericyclic reaction of conjugated bisallenes which has also been studied extensively over the years involves the Diels–Alder addition with both double and triple bond dienophiles, as well as heteroatom containing dienophiles. This is demonstrated by several examples in Scheme 38. Most classes of double-bond dienophiles react in the expected fashion and provide the Diels–Alder adducts in good yields, for example, **152**, **155**, **158** and **160**. Whenever there are two α-hydrogen atoms in the adduct aromatization can be accomplished by base treatment (e.g. formation of **153** and **156**). Incorporation of substituents into the double bond of the dienophile slows down the addition process or prevents it completely. Unusual dienophiles such as cyclobutadiene or various 1,2-disubstituted cyclobutenes do not add to **2** [122].

**Scheme 39:**
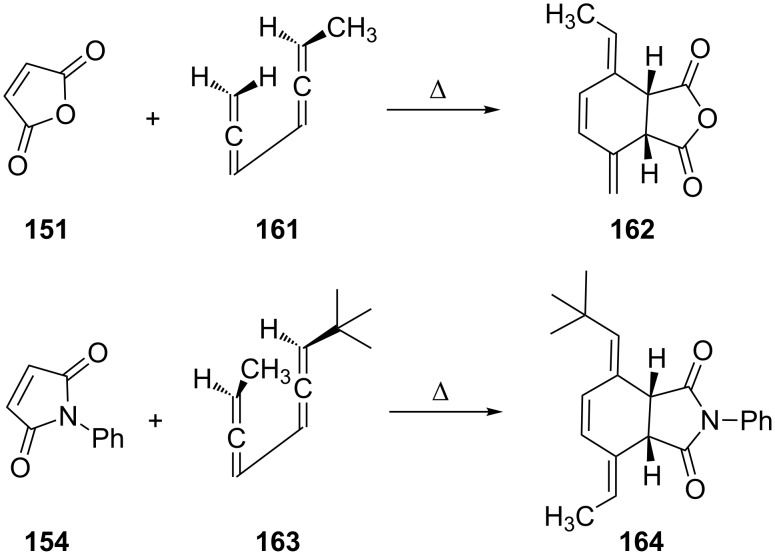
The stereochemistry of the [2 + 4] cycloaddition to conjugated bisallenes.

To investigate the stereochemistry of addition both 1,2,4,5-heptatetraene (**161**) [122] and the *erythro*-bisallene **163** [116] were subjected to Diels–Alder additions (Scheme 39) with maleic anhydride (**151**) and *N*-phenylmaleimide (**154**), respectively. In both additions the dienophiles attack the bisallene (in *s-cis* conformation) from the less hindered face to provide the diastereomers **162** and **164**, respectively, in an exclusive fashion.

**Scheme 40:**
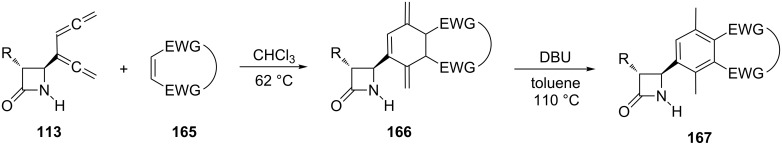
Preparation of azetidinone derivatives from conjugated bisallenes.

The azetidinones **113** described above (see Scheme 29) have also been employed in various cycloaddition experiments with double-bond dienophiles, **165**; the resulting Diels–Alder adducts **166** were subsequently aromatized to **167** by DBU-treatment (Scheme 40) [85].

**Scheme 41:**
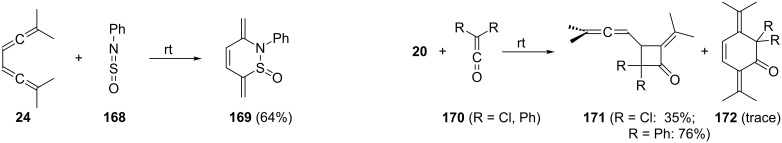
Cycloaddition of heterodienophiles to a conjugated bisallene.

Whereas the reaction of 1,1,6,6-tetramethyl-1,2,4,5-hexatetraene (**24**) with *N*-sulfinylaniline (**168**) gave the [2 + 4] cycloadduct **169** as the only isolable product (Scheme 41), the reaction of **24** with either dichloro (**170**: R = Cl) or diphenylketene (**170**, R = phenyl) furnished largely the [2 + 2] cycloadducts **171**. Only traces of the Diels–Alder adduct **172** are observed in this case. This example seems to be the only one in which the [2 + 4] and [2 + 2] cycloaddition mode compete [123]. Occasionally highly alkylated bisallenes such as **24** also provide ene-addition products.

**Scheme 42:**
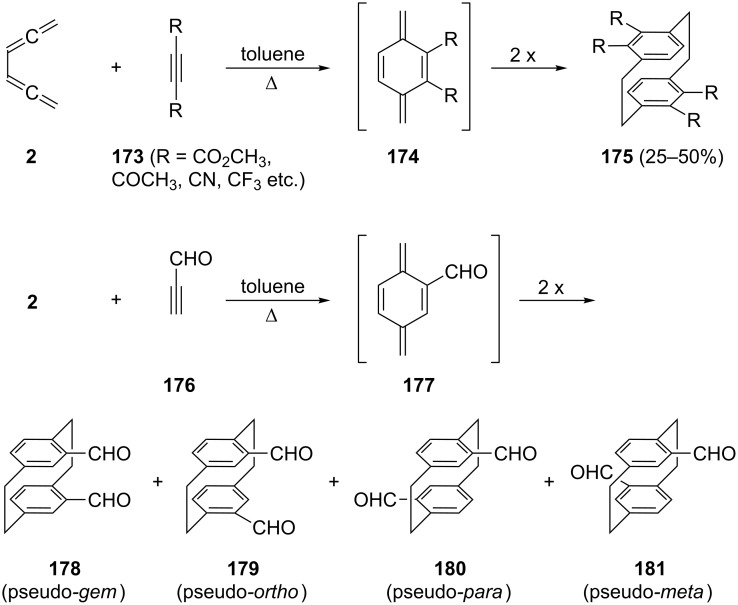
Addition of triple-bond dienophiles to conjugated bisallenes.

If the double-bond dienophile is replaced by an activated triple-bond dienophile **173**, another course of events is observed (Scheme 42) [21,124–126]. In this case the Diels–Alder step generates a *p*-xylylene intermediate **174** (a *p-*quinodimethane) first, which subsequently stabilizes itself by dimerization; the tetrasubstituted [2.2]paracyclophanes **175** are produced in acceptable yields (max. 50%). This route constitutes the preparatively most satisfactory way to obtain highly functionalized [2.2]paracyclophanes. Unactivated triple bonds do not undergo cycloaddition (no reaction with tolane, 2-butyne, cyclooctyne, etc.).

Switching to asymmetric triple-bond dienophiles, such as propiolic aldehyde (**176**), leads to a monosubstituted *p*-xylylene intermediate **177**, which has four different options to dimerize. These provide the isomeric bisformyl [2.2]paracyclophanes **178** to **181** in about equal ratio and in a total yield of up to 45% [127]. The apparently disadvantageous production of a mixture of isomers is not a real handicap of this route since these adducts differ in their physical properties (polarity, solubility) strongly enough to allow easy separation/purification by chromatography and/or recrystallization. The different isomers **178**–**181** are interesting starting materials in cyclophane chemistry in their own right. Other asymmetrically substituted triple-bond dienophiles that have been used like **176** include cyanoacetylene and methyl propiolate.

**Scheme 43:**
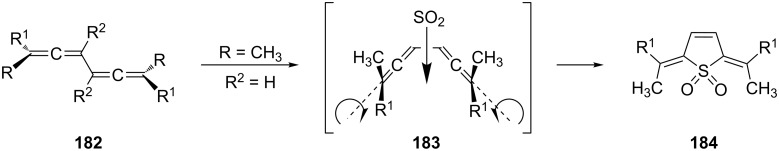
Sulfur dioxide addition to conjugated bisallenes.

A third group of pericyclic reactions of conjugated bisallenes that has been studied thoroughly concerns various cheletropic cycloadditions. The earliest studies in this area involve the addition of sulfur dioxide to different alkyl and aryl-substituted bisallenes (Scheme 43) [117]. The reaction yields the 1:1 adducts and from the stereochemistry of these products it must be concluded that the sulfur dioxide approaches the bisallenes from the least-hindered side and that the new bonds are formed under disrotation as illustrated in formula **183** [128].

**Scheme 44:**
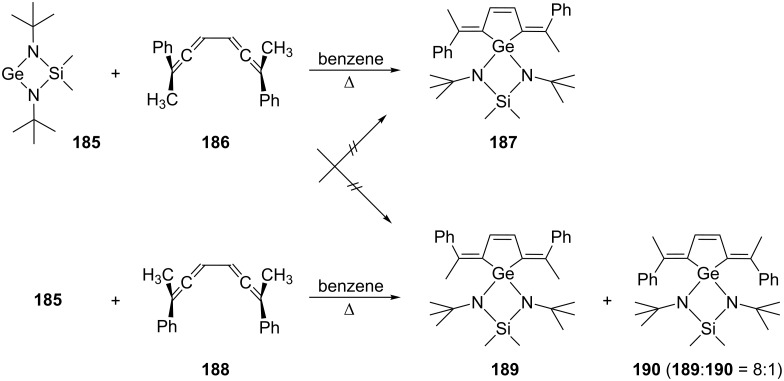
The addition of a germylene to a conjugated bisallene.

As far as the stereochemistry is concerned, several other addends behave similarly, as shown, e.g. by the germylene **185**, which provides the adduct **187** with **186** (Scheme 44). Likewise, the *meso*-compound **188** leads to **189** and **190**. There is no crossover between the two sets of reactions underlining their concerted nature. That **189** is the main product in the cycloaddition can be rationalized by the attack of **185** from the least shielded side of **188**, i.e. from the side of the methyl substituents. In summary, the germylene behaves as a singlet species in an orbital-symmetry-controlled cycloaddition (cheletropic, disrotatory cycloaddition) [129]. In an earlier study the Neumann group had already shown that stannylenes such as Me_2_Si(*tert-*BuN)_2_Sn or [(Me_3_Si)_2_CH]_2_Sn behave similarly towards the bisallenes **186** and **188** [130–132].

**Scheme 45:**
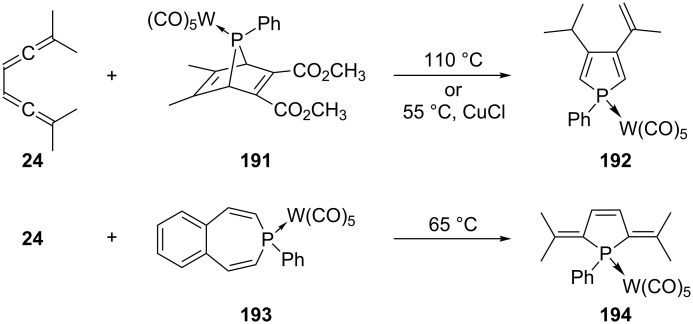
Trapping of conjugated bisallenes with phosphinidenes.

The tetramethylhexatetraene **24** was also reacted with various phosphinidenes generated in situ from various stable precursors. For example, the tungsten-complexed pentacarbonyl complex PhP=W(CO)_5_, generated by heating **191** (retro-Diels–Alder reaction), provided the unexpected 3,4-disubstituted phosphole **192** (Scheme 45) [133]. Analogously, the phospholene complex **194** resulted from heating **24** and **193** together [134].

**Scheme 46:**
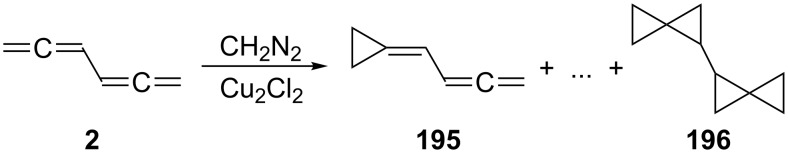
The cyclopropanantion of 1,2,4,5-hexatetraene (**2**).

Finally, as shown by Lüttke and Heinrich, the cyclopropanation of the parent system **2** with diazomethane/Cu_2_Cl_2_ provided a mixture of twelve cyclopropanation products in very good yield (ca. 90%), from the mono adduct **195** to the dispiro compound **196**, i.e. the 4:1 adduct (Scheme 46) [135]. The chemical behavior of these novel strained hydrocarbons has not been reported yet.

**Scheme 47:**
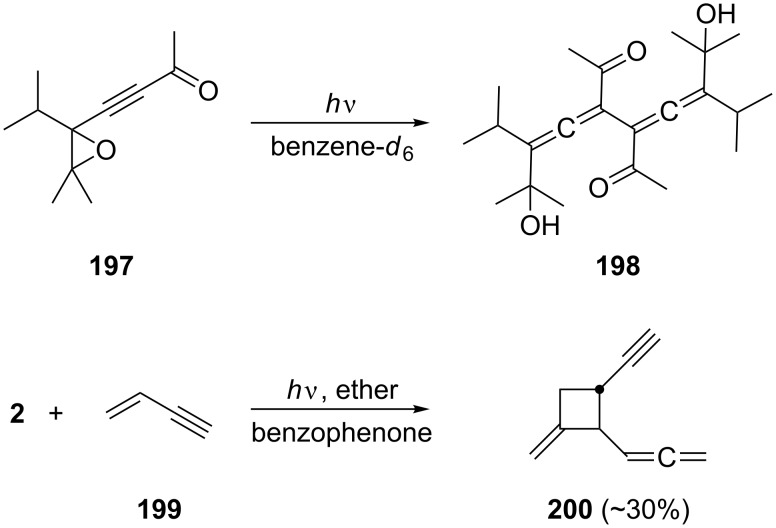
Photochemical reactions involving conjugated bisallenes.

In comparison to thermal (pericyclic) processes of conjugated bisallenes, very little is known about their photochemical behavior regardless of the mechanism of these processes. In fact we could only find two references describing photoreactions involving bisallenes. Thus, irradiation of the epoxide **197** in benzene-*d*_6_ (NMR experiment) yielded a plethora of photoproducts, among them two photodimers to which structure **198** was assigned by spectroscopic measurements (Scheme 47) [136]. In the second experiment, the parent hydrocarbon **2** was irradiated in the presence of vinylacetylene (**199**) and a triplet sensitizer (benzophenone). Besides the photodimers of **199**, the [2 + 2] photoadduct **200** was isolated in low yield [137].

**1.4.2 Ionic reactions**

Regardless of what types of reactions are summarized under this title, whether they involve carbocations or carbanions, or “only” polarized intermediates, ionic reactions involving **2** and its derivatives have only been studied to a limited extent so far. And this situation does not change very much if we include the chemical behavior of cyclic bisallenes as discussed in Section 4.2.

**Scheme 48:**
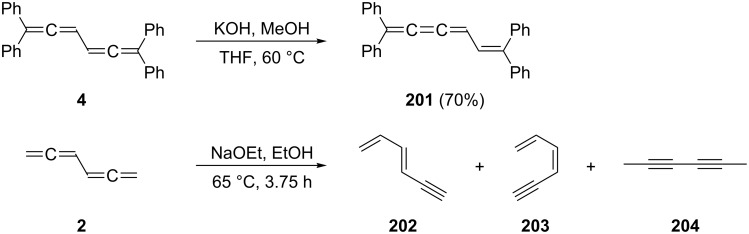
Base-catalyzed isomerizations of conjugated bisallenes.

Probably the earliest ionic reaction involving a conjugated bisallene concerns Kuhn's hydrocarbon **4** [9]. On treatment with base it isomerizes in good yield to the vinylbutatriene derivative **201**. Later it was shown that the parent system **2** isomerizes under comparable conditions into a mixture of **202**–**204** (Scheme 48) [39].

**Scheme 49:**
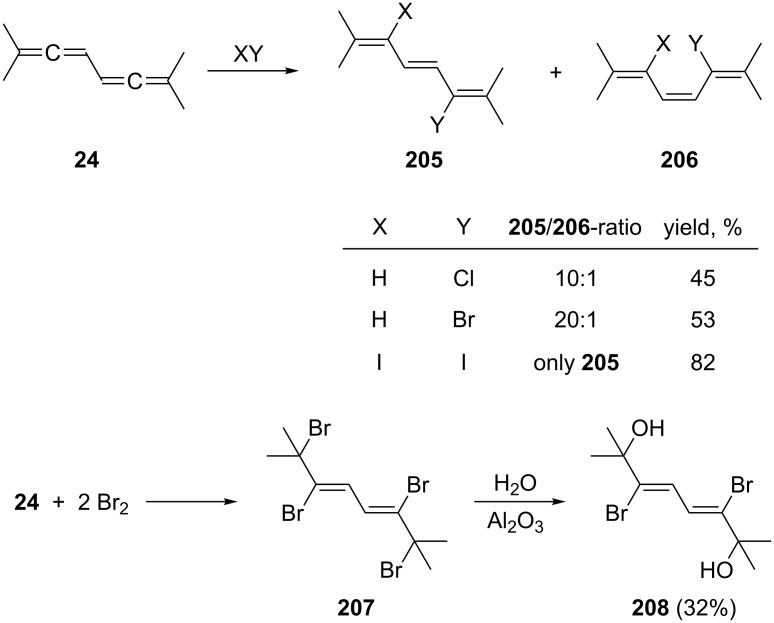
Ionic additions to a conjugated bisallene.

The addition of one equiv of protic acids (HCl, HBr) or iodine to 1,1,6,6-tetramethyl-1,2,4,5-hexatetraene (**24**), provided the diastereomeric 2,5-dihalogenated trienes **205** and **206** as shown in Scheme 49. Addition of excess bromine to **24** furnished the tetrabromide **207**, which on hydrolysis (H_2_O, Al_2_O_3_) provided the dibromo diol **208** [138].

**Scheme 50:**
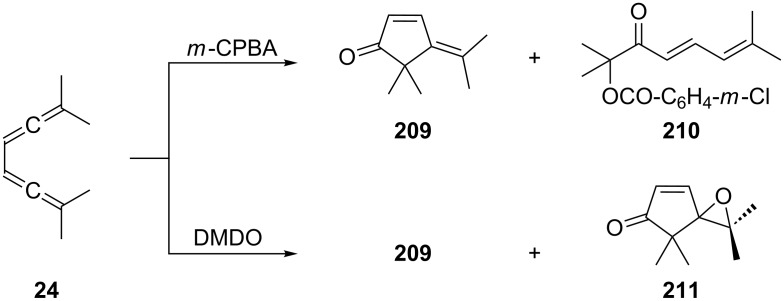
Oxidation reactions of a conjugated bisallene.

A careful study of the oxidation of several conjugated bisallenes with different oxidants has been carried out by Pasto and co-workers [139]. As summarized in Scheme 50, the tetramethylbisallene **24** yields two products with *meta*-chloroperbenzoic acid (*m*-CPBA): the alkylidenecyclopentenone **209**, and the “open” oxidation product **210** in whose formation the carboxylate anion resulting from the oxidation reagent is obviously involved. With dimethyldioxirane (DMDO) two products are produced again: **209** and the bisoxygenated product **211** for which **209** appears to be the most reasonable precursor. The oxidation of **24** with magnesium monoperoxyphthalate provides **209** in 32% isolated yield [138]. Looking at the structures of starting material and product **209** the process resembles the Nazarov cyclization of divinyl ketones to cyclopentenones [140].

**Scheme 51:**
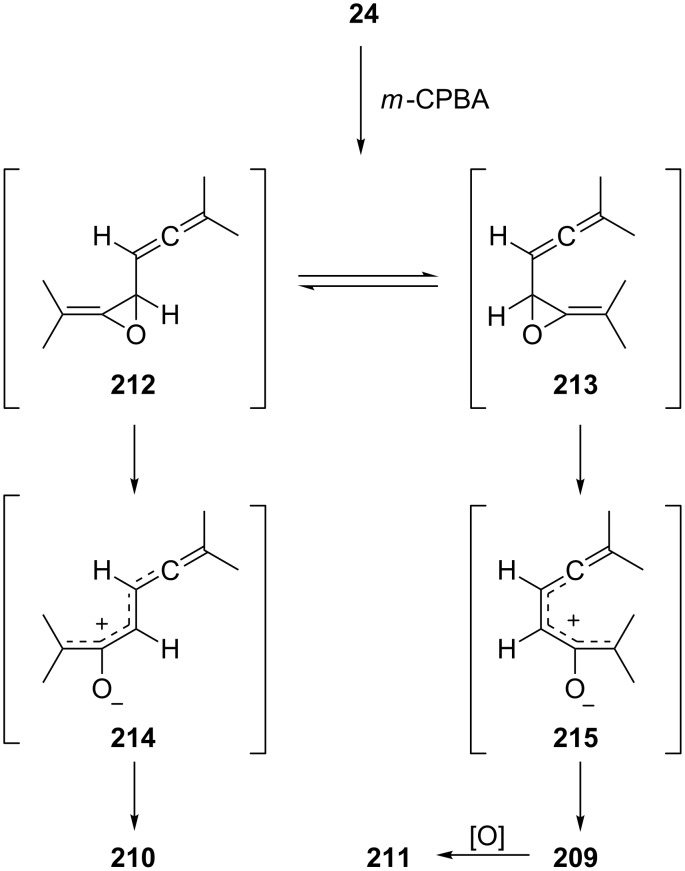
The mechanism of oxidation of the bisallene **24**.

Applying the mechanism discussed in this latter context to **24**, it is reasonable to propose the formation of an allene-epoxide first [141], which can exist either in a *transoid*, **212**, or *cisoid* conformation, **213** (Scheme 51). Whereas the former could open up to the “stretched” zwitterion **214**, which by carboxylate interception would lead to **210**, the more “closed” allenoxide **213** could isomerize to **215**, which on ring closure would furnish **209** and **211**, respectively. A 1:1 mixture of *erythro* and *threo*-8,8-dimethyl-2,3,5,6-nonatetraene behaves similarly, with the exception that this hydrocarbon is already oxidized by standing in air; the tetramethyl derivative **24** is stable towards air [139].

**1.4.3 Metal-induced reactions**

Although metal-initiated reactions (usually cyclizations) have become important ring-forming processes for the “higher” and heteroorganic bisallenes (see Sections 2.3 and 6.2), they are also known for conjugated bisallenes. We also would like to include metalation reactions in this chapter, namely the reactions we have already briefly referred to above (see Sections 1.1 and 1.2). Another example encountered earlier is the transformation recorded in Scheme 25, where silver and/or gold salts were employed to induce the cyclization of functionalized conjugated bisallenes.

**Scheme 52:**
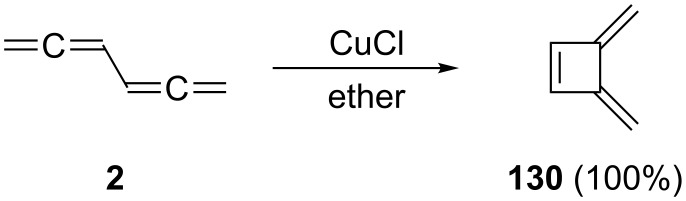
CuCl-catalyzed cyclization of 1,2,4,5-hexatetraene (**2**).

One of the first examples of a metal-catalyzed isomerization involves 1,2,4,5-hexatetraene (**2**) itself. When a suspension of **2** and CuCl in ether is stirred at room temperature the bisallene cyclizes to 3,4-bismethylenecyclobutene (**130**) in quantitative yield (Scheme 52) [142]. Many substituted bisallenes behave similarly and isomerize to the corresponding derivatives of **130** in a conrotatory manner [29].

**Scheme 53:**
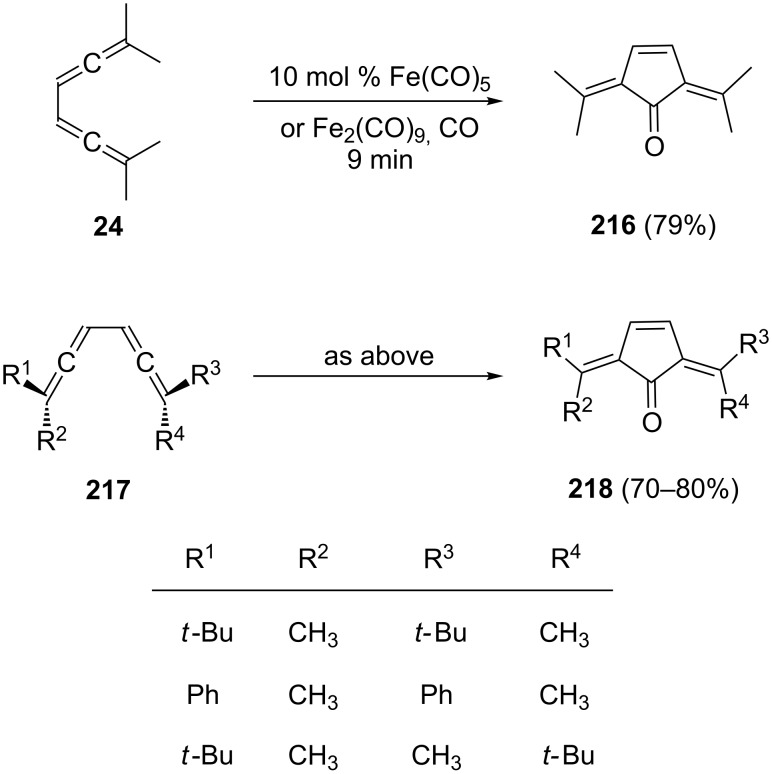
The conversion of conjugated bisallenes into cyclopentenones.

An interesting cyclization/functionalization of acyclic bisallenes was observed by Eaton and Rollman (Scheme 53) [102]. The cyclopentenone derivatives **216**/**218** are obtained in very good yields and the reaction takes place in a short time, as demonstrated for the model bisallene **24** in Scheme 53. In a later, more comprehensive study the scope and stereochemistry of the process were investigated thoroughly [24]. As can be seen in Scheme 53 the reaction takes place with high stereoselectivity, transferring the relative stereochemistry of the substrate bisallene to the product dialkylidenecyclopentenone. This outcome speaks for the η^4^-coordination of a *s-cis*-diallene conformation and rules out a mechanism whereby facial selectivity is determined by η^2^-coordination.

**Scheme 54:**
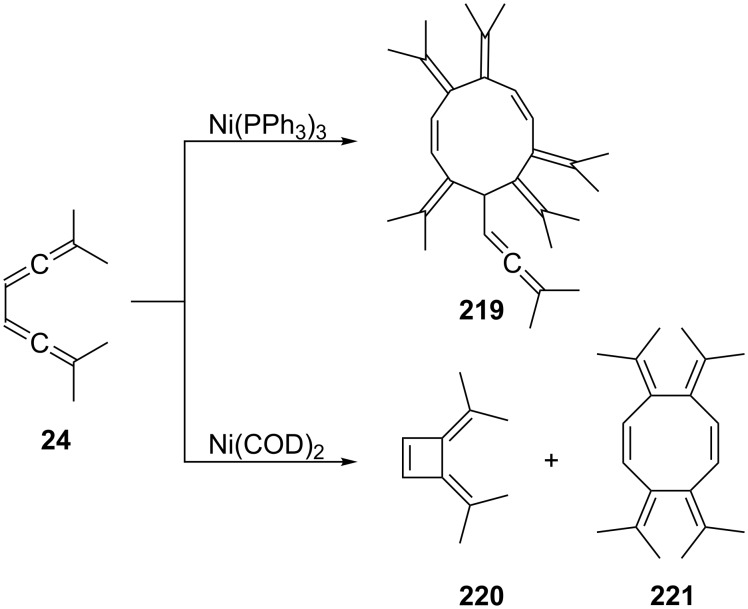
Oligomerization of a conjugated bisallene by nickel catalysts.

The behavior of **24** towards nickel catalysts has been studied by Pasto and Huang (Scheme 54) [143]. In the presence of Ni(PPh_3_)_3_ **24** trimerizes to the unusual ten-membered polyene **219**. When the reaction is carried out with Ni(COD)_2_ (COD = 1,5-cyclooctadiene) both the direct cycloisomerization product of the bisallene, hydrocarbon **220**, and the symmetrical dimer **221** are isolated. In the presence of the mixed ligand complex Ni(COD)(PPh_3_) a mixture of **220** and **221** is produced.

The metalations of bisacetylenic and bisallenic hydrocarbons already referred to (Section 1.2) [74–76] can probably also be exploited to introduce other substituents than TMS. Thus the quenching of the corresponding carbanions with, e.g., carbon dioxide to produce carboxylic acid derivatives of conjugated bisallenes could be tried [83,138,144].

### 2. Acyclic nonconjugated bisallenes

#### 2.1 1,2,5,6-Heptatetraene and its derivatives

**Scheme 55:**
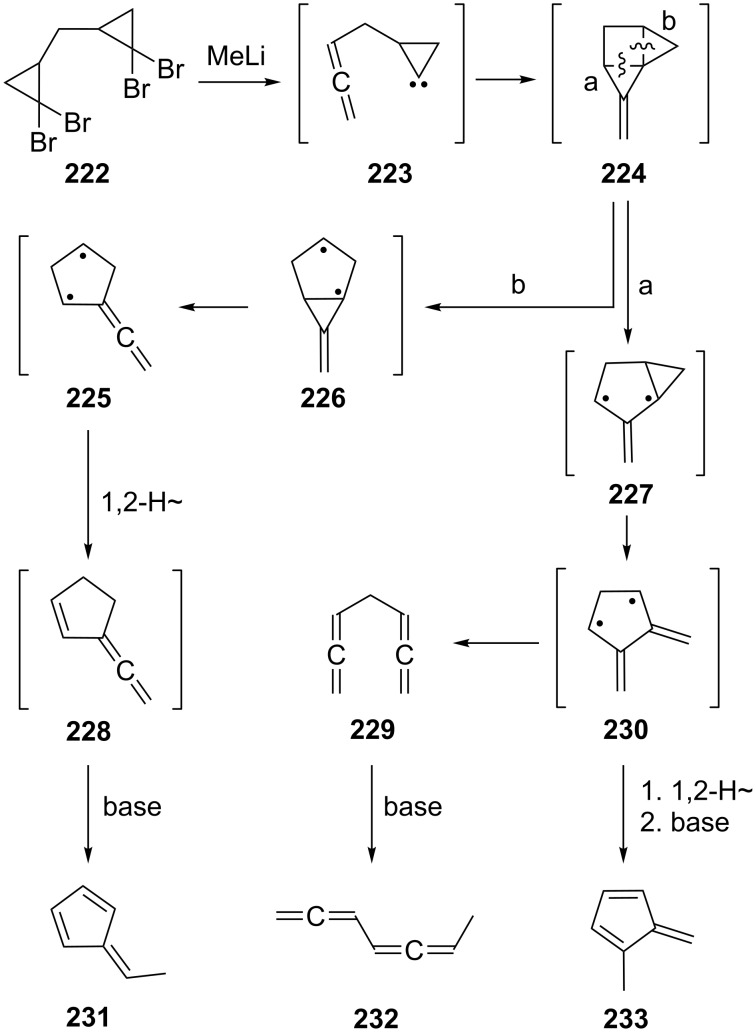
Generation of 1,2,5,6-heptatetraene (**229**) as a reaction intermediate.

The simplest conceivable acyclic, nonconjugated bisallenic hydrocarbon is 1,2,5,6-heptatetraene (**229**), a compound that has evidently escaped synthesis on a preparatively useful scale so far (Scheme 55). A reasonable route to prepare this hydrocarbon started from the dibromocarbene adduct of 1,4-pentadiene, the tetrabromide **222** [25,145]. However, when this was subjected to the DMS-allene synthesis (see Section 1.1), a rather complex mixture of hydrocarbons resulted in poor yield (ca. 10%), consisting of the two fulvenes **231** and **233** as well as 1,2,4,5-heptatetraene (**232**). A mechanism accounting for the formation of these unsaturated hydrocarbons is summarized in Scheme 55 and involves the generation of the intermediates **223** to **230**. According to this rationalization the immediate precursor of **232** is the desired but not isolated nonconjugated bisallene **229**.

**Scheme 56:**
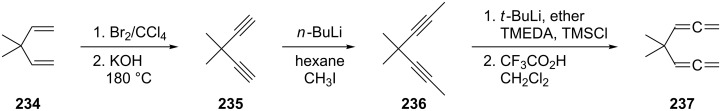
The preparation of a stable derivative of 1,2,5,6-heptatetraene.

That it is, indeed, the activated methylene moiety of **229** which plays a decisive role in its stability was demonstrated by the preparation of its 4,4-dimethyl derivative **237** by Roth and co-workers (Scheme 56) [146–147]. Starting from the geminally dimethylated 1,4-pentadiene **234**, these workers converted this diene by conventional steps via **235** into the 2,5-heptadiyne derivative **236**, which was subsequently subjected to a double prototropic rearrangement to yield the bisallene **237**.

**Scheme 57:**
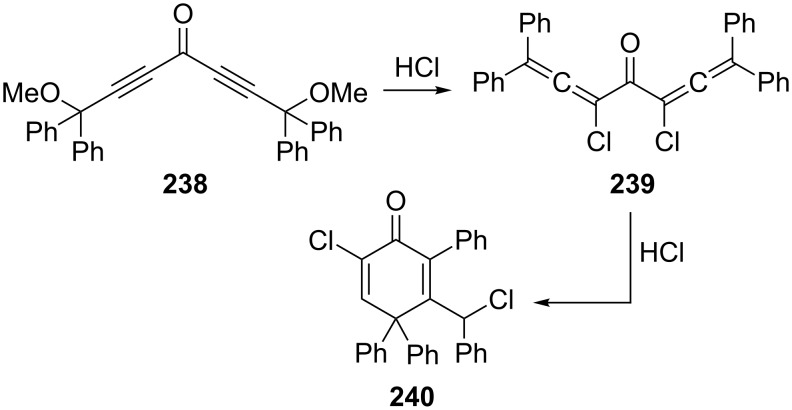
A bisallene with a carbonyl group as a spacer element.

Another way to remove the reactive α-hydrogen atoms in **229** consists of replacing them by functional groups. A case where this approach was successful has been reported by Ried and Marhold [148], who have prepared the dichloroketone **239** from **238** by HCl treatment. If **239** itself is exposed to hydrochloric acid for longer times it cyclizes to the cross-conjugated ketone **240** (Scheme 57).

#### 2.2 1,2,6,7-Octatetraene and its derivatives

**Scheme 58:**
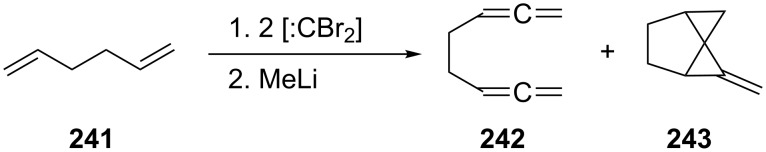
The first preparation of 1,2,6,7-octatetraene (**242**).

The next higher homologue of **229**, the C_8_-hydrocarbon **242** was first prepared by Skattebøl in the founding days of modern allene chemistry applying the DMS-protocol to 1,5-hexadiene (**241**, Scheme 58) [149]. The hydrocarbons **242** and **243** are produced in a total yield of 71% in 2.5:1 ratio, the tricyclic hydrocarbon **243** evidently generated by trapping of a cyclopropylidene intermediate by an already formed allene unit. Later work by the same authors showed that this approach can be extended to substituted [150] and also to functionalized [151] 1,2,6,7-octatetraenes. A direct conversion of α,ω-diynes into their chain-extended α,ω-diallenes by a Simmons–Smith reaction has also been described [152]; it yields mixtures of linear hydrocarbons, though, requiring chromatography for separation/purification.

**Scheme 59:**
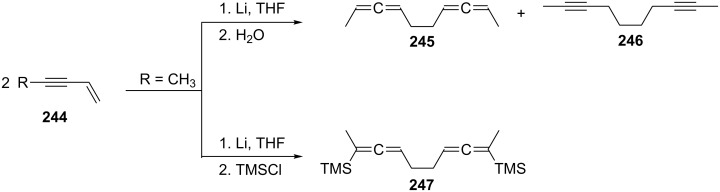
Preparation of 1,2,6,7-octatetraenes by (C_4_ + C_4_)-coupling of enynes.

Not surprisingly, several approaches to the C_8_-hydrocarbon system **242** by a (C_4_ + C_4_)-coupling protocol have been reported. These involve either enyne substrates or appropriately functionalized C_4_ building blocks. For example, alkyl and aryl-substituted enynes **244** on lithium treatment in THF followed by a water quench provide a mixture of the dimers **245** and **246** (Scheme 59) [153]. The dimer mixture is produced in acceptable yield (50–87%), and the bisallene is normally the dominant component. With trimethylsilyl chloride as the trapping agent, silyl-substituted bisallenes such as **247** are obtained.

**Scheme 60:**
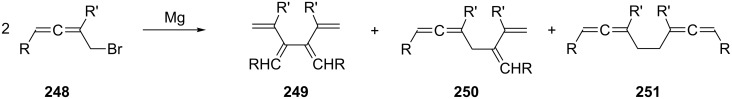
Preparation of 1,2,6,7-octatetraenes by (C_4_ + C_4_)-coupling of homoallenyl bromides.

The second approach referred to above, may be illustrated by the dimerization (Grignard coupling) of different (homo)allenic bromides **248** in the presence of magnesium (Scheme 60) [154]. Although the yields are poor (10–20%), the bisallenes **251** always constitute the main dimer of the reaction. Note that one of the dimers, **249**, has the structure of a [4]dendralene [48].

**Scheme 61:**
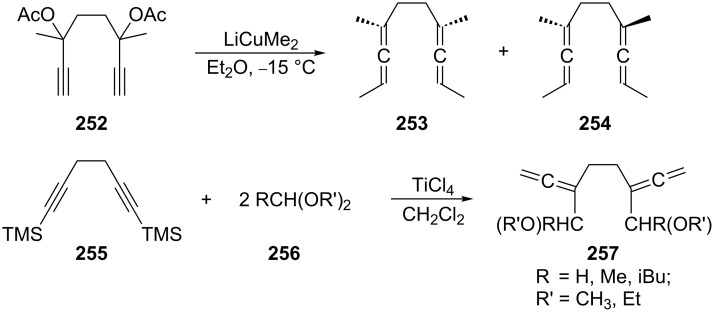
Preparation of 1,2,6,7-octatetraenes by alkylation of propargylic substrates.

Substituted bisallenes of type **242** have also been obtained by applying Crabbé’s cuprate method to dipropargylic diacetates (Scheme 61) [155], a protocol that has also been employed for the preparation of various cyclic bisallenes (see Section 4.2.2). The mixture of the diastereomers **253** (*meso*) and **254** (*d*,*l*) is produced in excellent yield (91%). Functionalized 1,2,6,7-octatetraenes **257** (ethers) are isolated in moderate yields (50–60%) when the protected bipropargyl derivative **255** was treated with different acetals **256** under the influence of titanium(IV) chloride. The resulting bisallenes **257** are useful substrates for the preparation of benzocyclobutenes, as will be discussed in Section 2.4 (Scheme 71) [156].

**Scheme 62:**
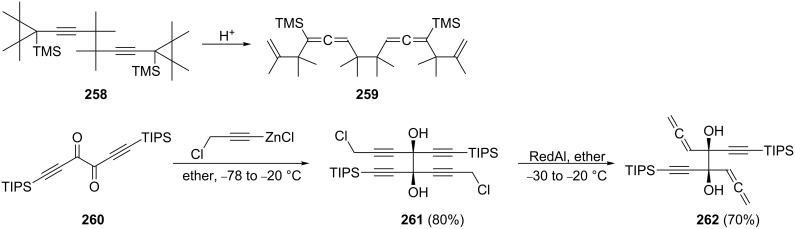
Preparation of two highly functionalized 1,2,6,7-octatetraenes.

We conclude this section with the preparation of two remarkable, highly substituted bisallenes of type **242** (Scheme 62). In the first transformation it was shown that the 1,5-hexadiyne derivative **258** on acid treatment undergoes an unusual rearrangement to the bisallene **259**. It is likely that the ring strain of the two cyclopropane rings contributes significantly to the driving force of the process [157]. The second reaction involves the diketone **260**, which on treatment with the ZnCl salt of propargyl chloride yields largely the *d*,*l*-diastereomer **261**. On reduction of **261** with Red-Al the bisallene **262** is obtained, which is a useful substrate for Pauson–Khand ring-formation reactions (see below, Section 2.4, Scheme 71) [158].

#### 2.3 Higher acyclic α,ω-bisallenes

Since the direct interaction of the two allene moieties is the most important preparative question related to acyclic bisallenes, it is not surprising that the number of publications dealing with the “higher” bisallenes, i.e. those where the spacer between the allene groups becomes longer and longer, is rather small and decreases with the length of the spacer. These more extended derivatives often just display the chemical behavior of two separate allene units. Still, surprisingly, a number of bisallenes has been described in which the number of unsubstituted (methylene) or substituted carbon atoms separating the allene units is three, four or even higher and reactions still take place between the allenic termini.

**Scheme 63:**
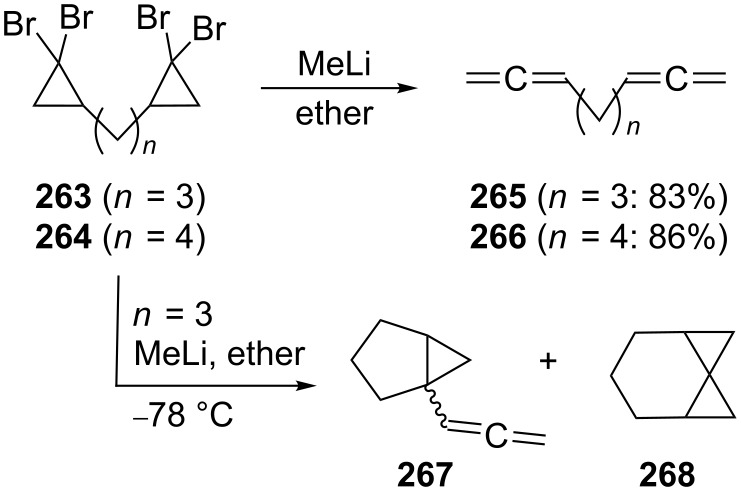
Preparation of several higher α,ω-bisallenes.

In fact many of the parent hydrocarbon systems are known, a case in point being **265** and **266**, which have been synthesized from the corresponding bis(dibromocyclopropane) derivatives **263** and **264** in very good yield by the DMS-route (Scheme 63) [149]. Actually the transformation is more complex, as was shown in a later investigation, at least at low temperatures. As demonstrated by Brinker [159] under these conditions besides, e.g. 1,2,7,8-nonatetraene (**265**), which is still the main product of the debromination/rearrangement, other hydrocarbons are formed, among them **267**, a formal 1,5-CH-insertion product, and **268**, which results by way of a 1,7-cycloelimination process (Scheme 63). The 1,2,8,9-decatetraene (**266**) has also been obtained by dimerization of 1-bromo-3,4-pentadiene with magnesium in THF (Grignard coupling) in 30% yield [160].

**Scheme 64:**
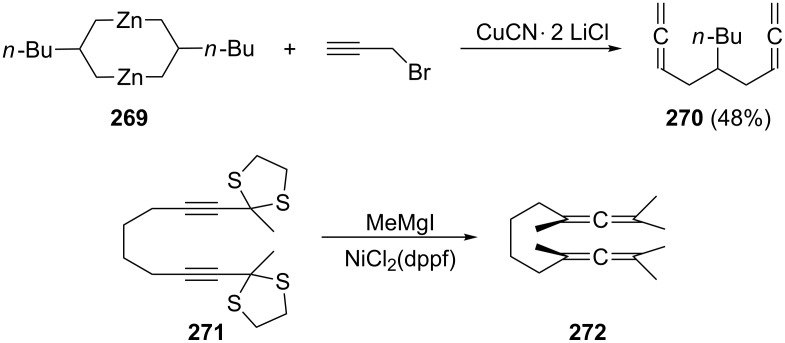
Preparation of different alkyl derivatives of α,ω-bisallenes.

Several alkyl derivatives of **265** and **266** have been prepared and although their importance in preparative organic chemistry is far lower than that of the functionalized derivatives described below, it is useful to have access to them for spectral comparison. For example, coupling of the bismetallic reagent **269** with propargyl bromide provides the *n*-butyl derivative of **265**, the branched hydrocarbon **270**, in 48% yield (Scheme 64) [161]. The Ni-catalyzed cross-coupling reaction of the diacetylenic dithioketal **271** with Grignard reagents such as methylmagnesium iodide furnished the hexamethyl derivative of bisallene **266**, hydrocarbon **272** [162].

**Scheme 65:**
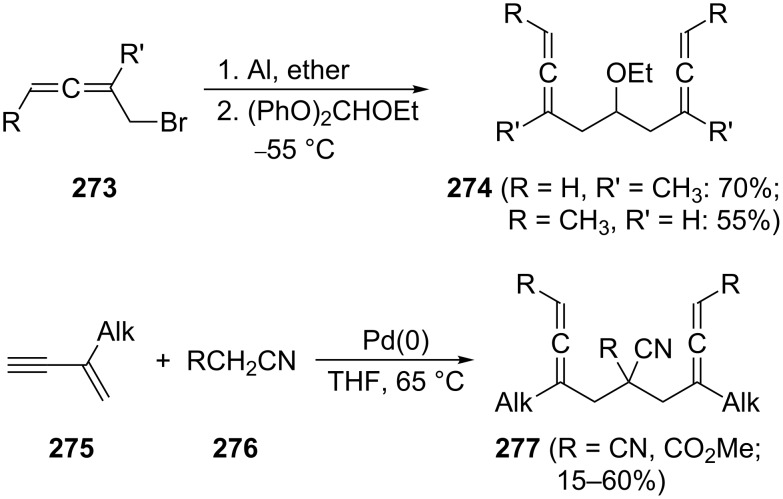
The preparation of functionalized 1,2,7,8-nonatetraene derivatives.

For the preparation of functionalized derivatives of **265** and **266**, as well as those in which their saturated methylene groups have partially been replaced by heteroatoms (O, N, see Section 6 below) or carry functional groups, a substantial number of methods exist. In an early study by Miginiac and Barbot the ethers **274** were prepared by reacting orthoesters at low temperatures with homoallenic bromides **273** (Scheme 65) [163]. Various cyanoesters or malononitriles, **276**, have been added to conjugated enynes **275** in the presence of a Pd-catalyst to generate the bisallenic nitriles **277** in fair yields [164].

**Scheme 66:**
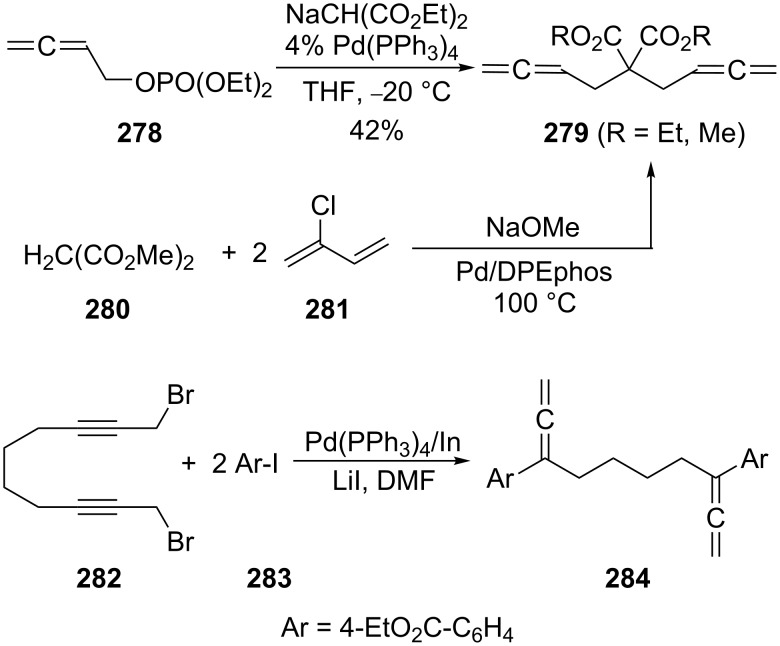
Preparation of functionalized α,ω-bisallenes.

Similar yields of the diallenic diesters **279** were obtained in the coupling of esters of α-allenic alcohols (acetates and in particular phosphates such as **278**) with the anions of β-diesters, β-ketoesters and α-phenylsulfonyl esters in the presence of catalytic amounts of palladium(0) complexes such as Pd(PPh_3_)_4_ (Scheme 66) [165–166]. The diester **279** is also obtained when unsubstituted malonate **280** is treated with excess 2-chloro-1,3-butadiene (**281**, chloroprene) and 2.5 equiv of sodium methoxide in the presence of Pd/DPEphos [167]. Another Pd-mediated coupling experiment, leading to a derivative of **266** involves the dibromide **282** and the aromatic haloester **283**; the bisallene derivative **284** was obtained in 68% yield [168].

**Scheme 67:**
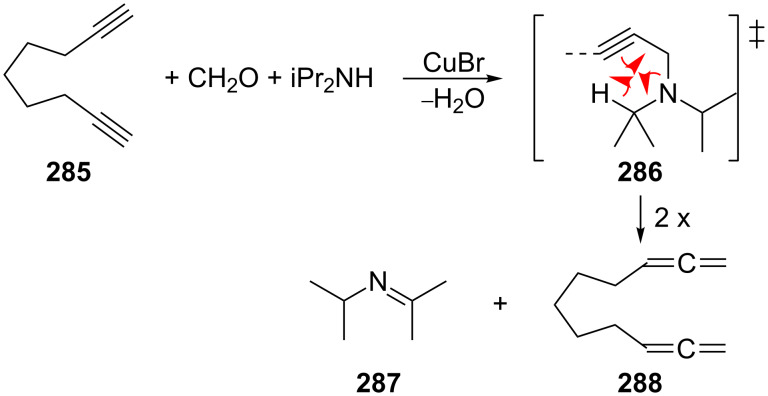
The preparation of an α,ω-bisallene by direct homologation of an α,ω-bisalkyne.

Extended α,ω-bisallenes are often prepared from the corresponding α,ω-dienes by the DMS-route (see Section 1.1 above), but a direct homologation of an α,ω-diyne has also been reported. Thus, 1,2,9,10-undecatetraene (**288**) was prepared by Crabbé and co-workers from 1,8-nonadiyne (**285**) by a route involving the cuprous bromide-catalyzed homologation of the diacetylene with formaldehyde and diisopropyl amine in dioxane under reflux (Scheme 67) [169]. A mechanism has been proposed for this reaction involving a CuBr-catalyzed Mannich reaction step followed by the pericyclic fragmentation of the initially produced intermediate **286** to the bisallene **288** and the imine **287**.

As far as the still more elongated α,ω-bisallenes are concerned, it is surprising to what extent intramolecular reactions are observed once the spacer element is rigidified by, e.g., aromatic subunits. The corresponding examples will be discussed in greater detail below (see Section 4.1).

#### 2.4 The chemical behavior of nonconjugated, acyclic α,ω-bisallenes

The structurally simplest of these hydrocarbons is 1,2,5,6-heptatetraene (**229**), and its base-induced isomerization to 1,2,4,5-heptatetraene (**232**) has already been referred to in Scheme 55.

**Scheme 68:**
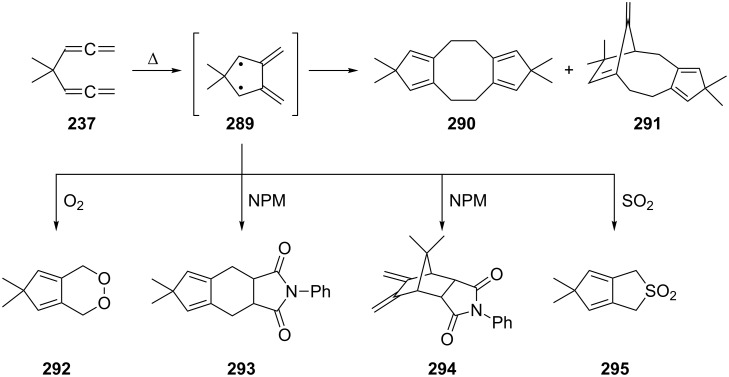
The gas-phase pyrolysis of 4,4-dimethyl-1,2,5,6-heptatetraene (**237**).

A thorough study of the thermal behavior of its 4,4-dimethyl derivative **237** was carried out by Roth, Maier and co-workers. As illustrated in Scheme 68, on heating, the dimethyl-substituted, and hence protected, hydrocarbon **237** first cyclizes to the diradical **289**, an intermediate that can also be produced from other precursors [146]. In the absence of trapping agents, 2,2-dimethyl-4,5-dimethylene-1,3-cyclopentanediyl (**289**) dimerizes to two dimers, i.e. **290** and **291**, whereas interception with oxygen leads to **292**, with *N*-phenylmaleimide (NPM) to adducts **293** and **294**, and with sulfur dioxide to sulfolene **295**. As we shall see below, structurally similar compounds are formed when the higher homologues of **229**/**237** are pyrolyzed.

**Scheme 69:**
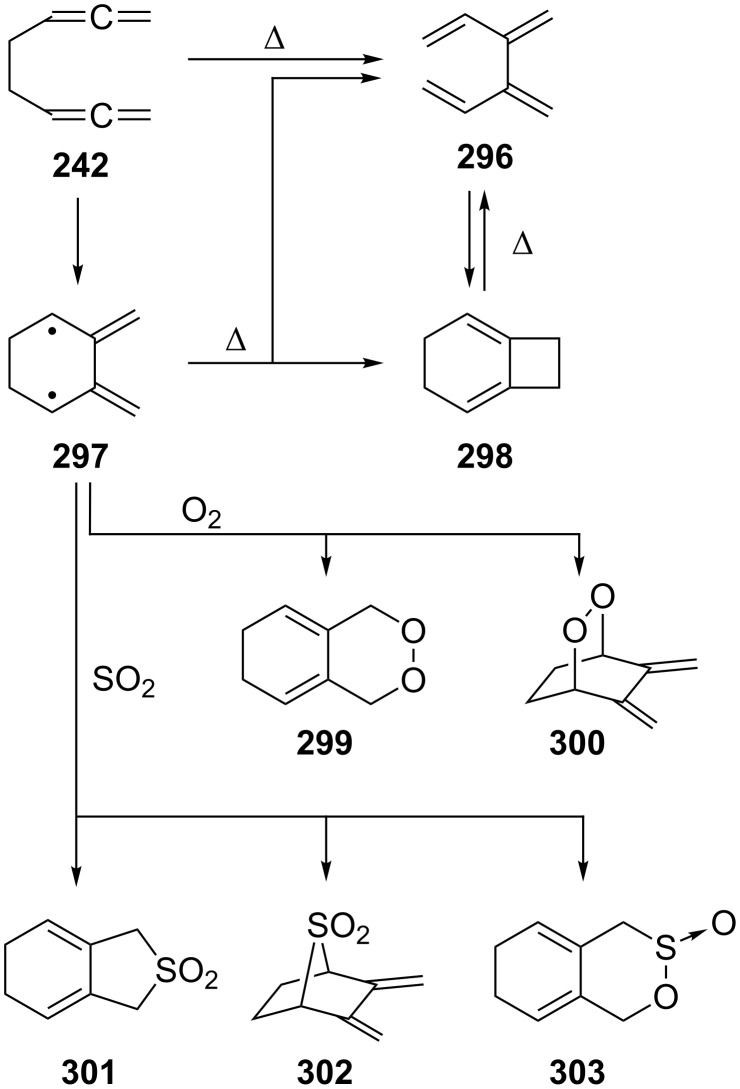
Gas-phase pyrolysis of 1,2,6,7-octatetraene (**242**).

The thermal cyclization of 1,2,6,7-octatetraene (**242**) was first studied by Skattebøl and Solomon (Scheme 69) [170]. On being heated in the gas phase at 310 °C hydrocarbon **242** rearranges quantitatively into 3,4-dimethylene-1,5-hexadiene (**296**). What formally may be regarded as an allenic variant of the classical Cope-rearrangement of 1,5-hexadiene (a subsystem of **242**) is actually considerably more complex, as was shown by Roth [171–173], Grimme [174] and others. In fact not only **296** is generated under pyrolysis conditions, but also the C_8_H_10_ isomer bicyclo[4.2.0]octa-1,5-diene (**298**), and it was shown that the two hydrocarbons are in thermal equilibrium. As above, the diradical intermediate **297** may be trapped by added gases such as oxygen or sulfur dioxide to provide the 1:1 adducts **299**–**303**.

The *meso*- and *d*,*l*-2,3,7,8-decatetraenes behave similarly and suggest the formation of diradicals of type **297** in these cases also [150]. A computational study (quantum chemical CI calculations with the semiempirical MO method SINDO1) on the C_8_H_10_ network involving the above hydrocarbons and intermediates has been carried out by Jug and Iffert [175].

**Scheme 70:**
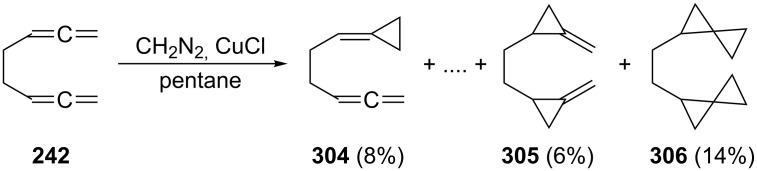
The cyclopropanation of 1,2,6,7-octatetraene (**242**).

The cyclopropanation (CH_2_N_2_, CuCl) of **242**, performed by de Meijere and Kaufmann [176], gave all possible mono- to tetraadducts. As in the case of **2** (see Section 1.4.1, Scheme 46) Scheme 70 only shows a selection of these nine products, **304**–**306**; to separate and identify these rather similar cyclopropanes constitutes an impressive experimental feat. Again, it would be of interest to know, e.g. the thermal behavior of these hydrocarbons.

**Scheme 71:**
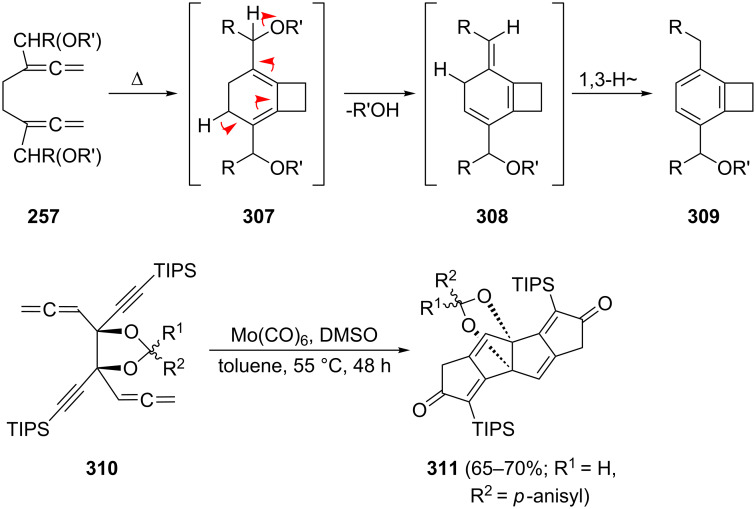
Intramolecular cyclization of 1,2,6,7-octatetraene derivatives.

As far as derivatives of **242** carrying functional groups are concerned, we mention the already referred to bisallenic diethers **257**, which, under pyrolysis conditions, cyclize in good yields (55–80%) to substituted benzocyclobutenes **309** involving the intermediates **307** and **308**, as shown in Scheme 71 [156]. A second transformation involves the remarkable Pauson–Khand reaction of the highly functionalized 1,2,6,7-octatetraene derivative **310**, which was already encountered in the form of the diol **262** in Scheme 62; with molybdenum hexacarbonyl it cyclizes to the dicyclopenta[*a*,*e*]pentalene derivative **311** [158].

**Scheme 72:**
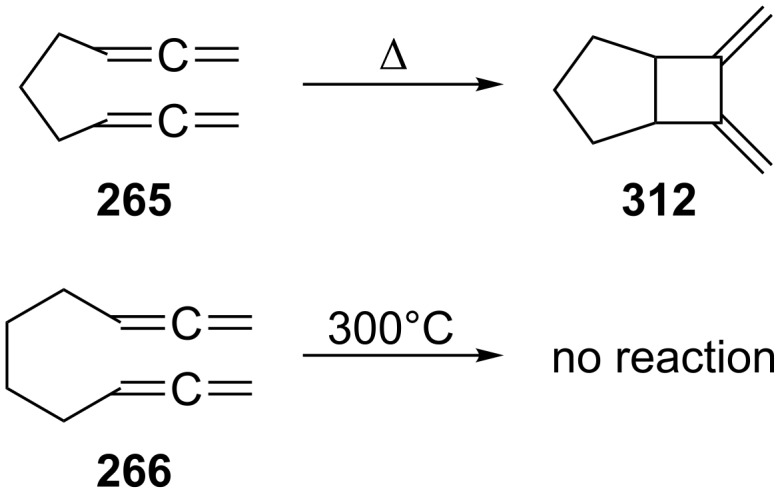
The gas-phase pyrolysis of 1,2,7,8-nonatetraene (**265**) and 1,2,8,9-decatetraene (**266**).

The next higher homologues of **242**, 1,2,7,8-nonatetraene (**265**, see Scheme 63) and 1,2,8,9-decatetraene (**266**), were also subjected to gas-phase pyrolysis at 390 and 300 °C, respectively, by Skattebøl and Solomon (Scheme 72) [170]. The former hydrocarbon cyclizes in an intramolecular [2 + 2] cycloaddition to **312**, i.e. it undergoes the reaction that is so typical (intermolecularly) for simple monoallenes; whereas the latter does not react, possibly as a consequence of the distance between the allene units being too large. The yield of the **265**→**312** transformation is poor (32%) and the not fully characterized product mixture does not allow the proposal of a mechanism for this thermocyclization. When the temperature for **266** is increased to 440 °C a product mixture of unknown composition is obtained in low yield.

Recent years have witnessed numerous reports on the use of functionalized derivatives of **265** and **266** in preparative organic chemistry. In principle any position of these bisallenes can carry a functional group (or several ones). In practice, however, it is often the internal allene carbon atom and/or the central methylene group of, e.g., **265** that carries substituents. Quite often methylene groups of the tether connecting the allene moieties are replaced by hetero atoms also, in particular by nitrogen and oxygen, as we shall see in Section 6.

**Scheme 73:**
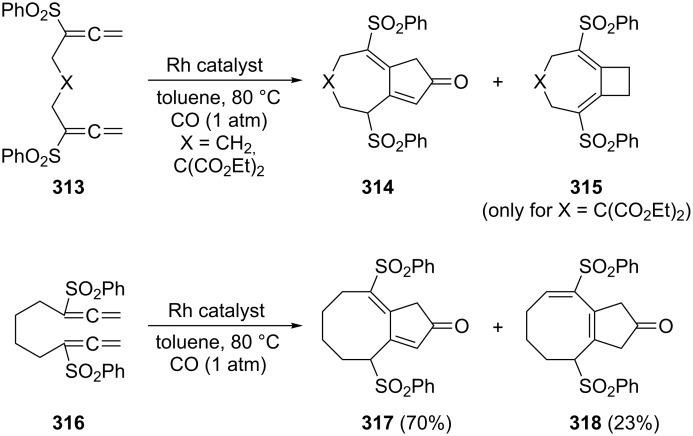
Rh-catalyzed cyclization of a functionalized 1,2,7,8-nonatetraene.

A typical example for the above strategy has been described by Mukai and co-workers (Scheme 73) involving a Pauson–Khand reaction with the bisallene bissulfone **313** (X = CH_2_) [177]. On treatment with various rhodium catalysts under carbon monoxide it cyclizes in near quantitative yield to the bicyclo[5.3.0]decadienone compound **314** (X = CH_2_). The “allene dimerization product” **315** is not produced. With X = C(CO_2_CH_3_)_2_, however, **315** becomes a side product and, with certain rhodium catalysts, even the main product of the cyclization (up to 70%). Heteroorganic variants of this process (X = heteroatom) are known and will be discussed in Section 6. Non-sulfonylated bisallenes do not undergo this [2 + 2 + 1] cycloaddition, which probably involves the intermediate generation of a rhodacycle. The C_4_-tethered derivative **316** behaves analogously, furnishing the bicyclic ketone derivatives **317** and **318**. Even chain extension to five carbon atoms still permits some intramolecular bicyclization. Heating **316** in the absence of CO, but under the influence of [{RhCl(CO)dppp}_2_], causes cyclization to the corresponding [3]dendralene derivative in excellent yield (92%) [48,177].

**Scheme 74:**
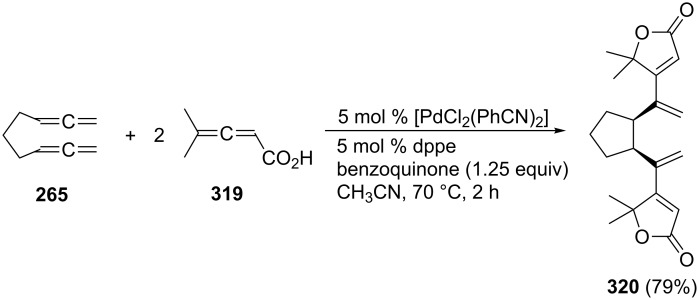
A triple cyclization involving two different allenic substrates.

A remarkable triple cyclization of two molecules of the 2,3-allenoic acid **319** in the presence of one molecule of the parent bisallene **265** has recently been described by Ma and Lian (Scheme 74) [178]. The reaction yields the tricyclic compound **320** in excellent yield considering what has been accomplished preparatively.

**Scheme 75:**
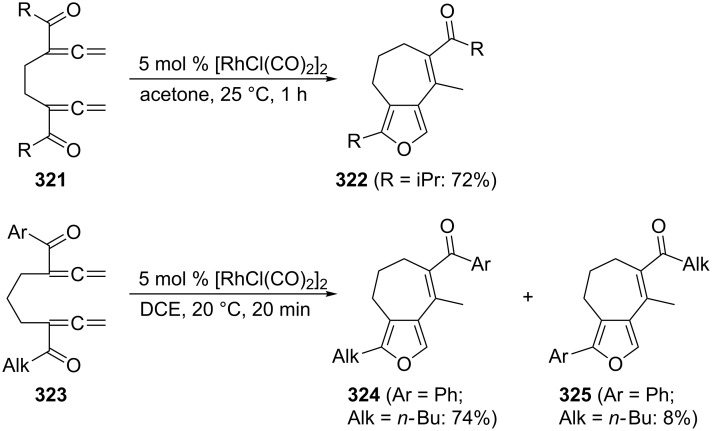
Bicyclization of keto derivatives of 1,2,7,8-nonatetraene.

The group of Ma has revealed interesting cyclization reactions of various aliphatic and aromatic diketones derived from **242** and **265** (Scheme 75) [179]. For example, diketone **321** isomerizes under very mild conditions into the bridged furan derivative **322**. Making the two substituents different, as in **323**, causes the cyclization to provide two differently substituted furans, **324** and **325**.

**Scheme 76:**
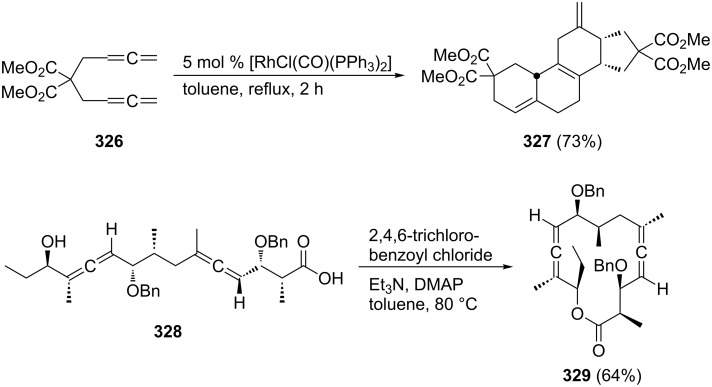
The preparation of complex organic compounds from functionalized bisallenes.

Many other multifunctionalized bisallenes derived from **265** have been cycloisomerized similarly and constitute an important high-yielding and multifaceted route to interesting bi- and polycyclic compounds [180–187], as the following examples show and with which we wish to conclude this section on cyclization reactions of α,ω-bisallenes. In Scheme 76 the first reaction displays a Rh-catalyzed cycloisomerization/dimerization in which the diester **326** is converted into a product with the carbon skeleton of a steroid, **327** [188–189]. To obtain the high yield given in the scheme it is mandatory to reduce the concentration of **326** to low levels (0.04 M). The second example concerns the lactonization of a bisallenic hydroxy acid **328**, which leads to a macrocyclic bisallene **329**, a pivotal intermediate for the preparation of various erythronolides (see Scheme 108 below); with 64% the yield of the process is remarkable [190].

**Scheme 77:**
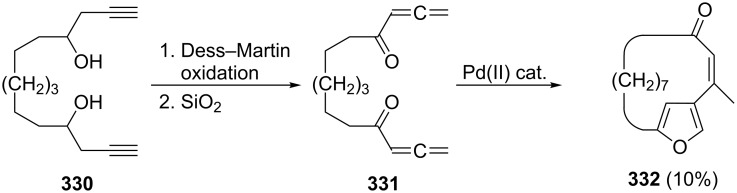
Cycloisomerization of an α,ω-bisallene containing a C_9_ tether.

A bisallene in which the two allene moieties are separated by no less than nine carbon atoms is the diketone **331**, which is obtained from the diol **330** by a Dess–Martin oxidation, followed by propargylic isomerization on workup (column chromatography on silica gel; Scheme 77) [191]. Treatment of **331** with various Pd(II)-catalysts causes cyclization to different furanophanes, among them **332**, which is produced in 10% yield. In fact, the spacer between the two carbonyl groups may be extended up to 14 methylene groups and the combined yields of the resulting macrocycles are still astonishingly high (up to 30–40%).

**Scheme 78:**
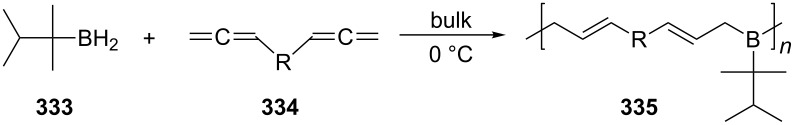
Organoborane polymers from α,ω-bisallenes.

Since α,ω-bisallenes are bifunctional compounds, the question may be asked whether they could be used as monomers for the preparation of new polymeric materials. The answer is yes and not surprisingly it involves the more elongated bisallenes preferentially. For example, the diester **326**, which has already been shown to be one of the work horses in intermolecular cyclization reactions (see above), was polymerized with an allylnickel catalyst [192]. The resulting polymer, which is soluble in common organic solvents, is composed of an almost comparable content of 1,2 and 2,3-polymerized units. In another polymerization experiment, the α,ω-bisallenes **334** (R = –(CH_2_)_4_–, –(CH_2_)_6_– and –*para*-C_6_H_4_–) were subjected to a hydroboration experiment with thexylborane (**333**) and other organoboranes (Scheme 78) [193]. The obtained organoborane polymers **335** were submitted to chain-transformation reactions, leading, e.g., to the corresponding polyalcohols.

### 3. Bisallenes with unsaturated spacer elements

As delineated in the Introduction, unsaturated moieties can be used in many different ways to connect two allene units (see structures **8**–**12** in Figure 3).

**Scheme 79:**
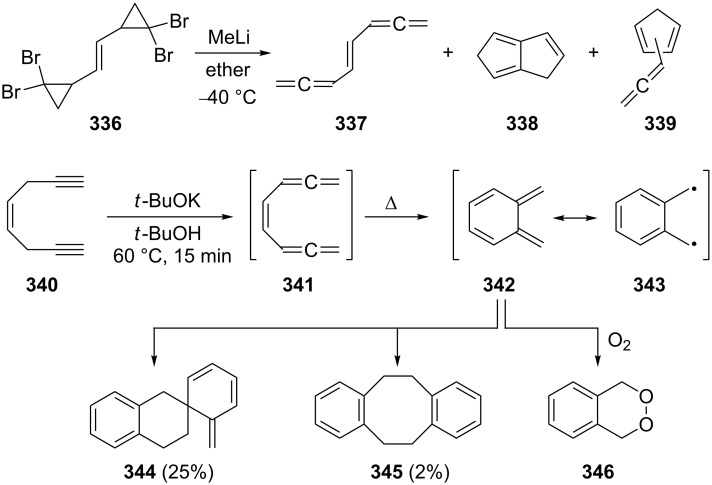
Preparation of *trans-* (**337**) and *cis*-1,2,4,6,7-octapentaene (**341**).

One of the simplest unsaturated spacers is the carbon–carbon double bond. When interspersing it between two allene units 1,2,4,6,7-octapentaene results, which may be present as the *trans*- or the *cis*-diastereomer, **337** [194] and **341**, respectively (Scheme 79). Although the *E*-diastereomer **337** is indeed formed when the double dibromocyclopropanation product of *E*-1,3,5-hexatriene, i.e., the tetrabromide **336**, is subjected to the DMS protocol (see above, Section 1.1), the overall yield of the reaction is low (32%), and hydrocarbon **337** is not the only reaction product. For example, the five-membered-ring products **338** and **339** are also among the debromination products. The formation of cyclopentadiene-derived products, notably fulvenes, has often been noted in this transformation (see above) [26,194].

The *Z*-diastereomer of **337**, the pentaene **341**, is apparently unknown as an isolated product up to the present day. It was postulated as early as 1963 by Sondheimer and Ben-Efraim to be generated from the diacetylene **340** by base treatment (*t*-BuOK, *t*-BuOH) [195]. That it actually had been generated in the above process was inferred from the isolation of two dimers, **344** and **345**, formed by a [2 + 4] and a [4 + 4] cycloaddition process, respectively. In the presence of oxygen the *endo*-peroxide **346** is produced. The instability of **341** was rationalized by its rapid cyclization to *o*-xylylene, **342**/**343**, under the reaction conditions. A very similar process has been observed for those molecules in which the double bond in **341** has been replaced by a benzene or naphthalene ring as will be discussed below (see Section 4.1).

**Scheme 80:**
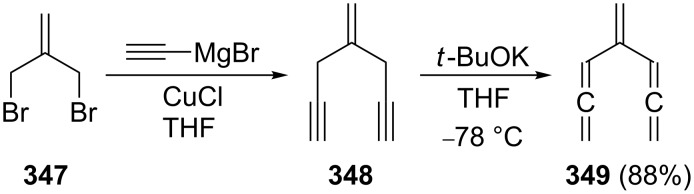
The preparation of 4-methylene-1,2,5,6-heptatetraene (**349**).

If the allene groups are not bonded to the ethylene unit in vicinal fashion, as in **337**/**341**, but geminally, the hydrocarbon **349** results (Scheme 80). This cross-conjugated pentaene was prepared from its isomer **348** by a base-induced isomerization again, and the diacetylene was obtained by coupling the dibromide **347** with ethynylmagnesium bromide. The characterization of **349** rests on spectroscopic data as well as the formation of a Diels–Alder adduct when it is treated with tetracyanoethylene (TCNE) [196–197].

**Scheme 81:**
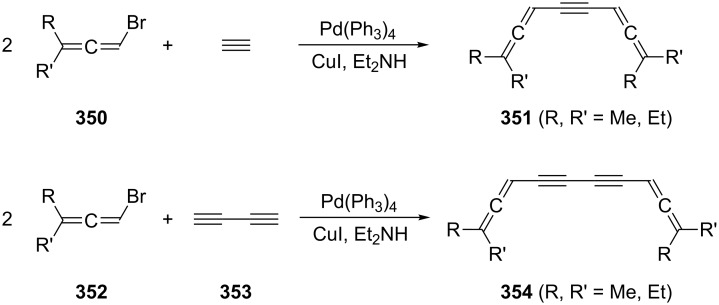
The preparation of acetylenic bisallenes.

Several derivatives of **2** are known with interspersed acetylenic functions. One of the simplest hydrocarbons in this category is **351**, which results when acetylene is bubbled through a solution of 1-bromo-3,3-dialkylallenes **350** in diethylamine in the presence of Pd(PPh_3_)_4_ and CuI (Scheme 81) [198]. The products **351** are crystalline solids, which are produced in acceptable yields (30–50%). Analogously, **354** is produced (in yields up to 62%) when 1,3-butadiyne (**353**) is employed as the coupling partner. Both **351** and **354** can be converted into dianions by treatment with *n*-butyllithium. The quench with trimethylsilyl chloride subsequently provided the respective bis-silylated derivatives.

**Scheme 82:**
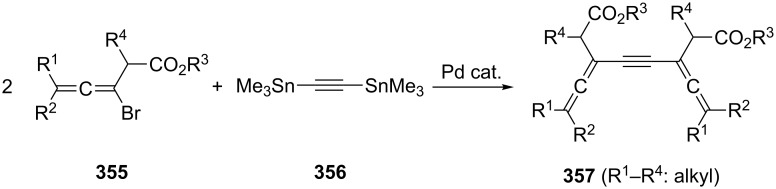
The preparation of derivatives of hydrocarbon **351**.

In a comparable, now Stille-type coupling reaction, Saalfrank and co-workers connected two molecules of **355** with bis(trimethylstannyl)acetylene (**356**) to furnish the diester derivative **357** (Scheme 82) [199].

**Scheme 83:**
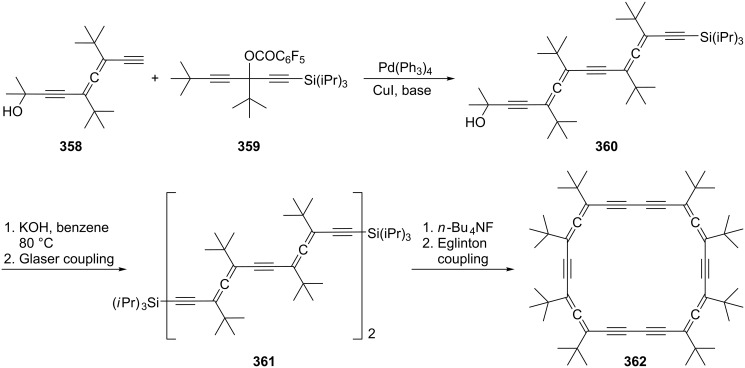
The construction of macrocyclic alleno-acetylenes.

More complex allenynes were prepared by Diederich and co-workers as building blocks for shape-persistent chiral alleno-acetylenic macrocycles and cyclophanes. A typical example is shown in Scheme 83 [200]. By Pd-catalyzed coupling of the building blocks **358** and **359** the bisallene diyne **360** was first generated under propargylic rearrangement (S_N_2'). The protecting dimethylcarbinol function was then cleaved off by base treatment (so-called Favorskii cleavage) and the resulting terminal acetylene dimerized to **361** by Glaser coupling. In the final ring-closing step the tris(isopropylsilyl)-protecting groups were removed by fluoride treatment and the resulting α,ω-diacetylene was oxidatively cyclized by Eglinton coupling. The macrocyclic product **362** was produced in the form of two pairs of enantiomers and three achiral diastereomers.

**Scheme 84:**
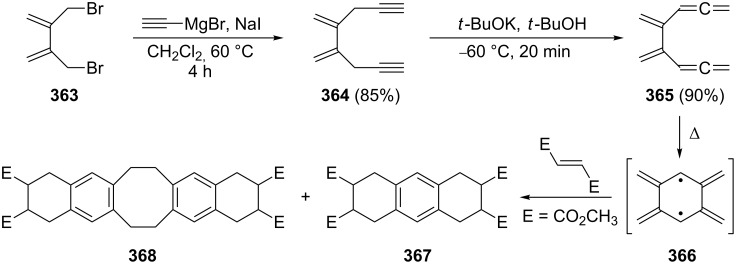
Preparation and reactions of 4,5-bismethylene-1,2,6,7-octatetraene (**365**).

Clearly, many more unsaturated spacer units can be designed (and prepared) and we would like to close this section with just one final example from the Roth group (Scheme 84) [146]. Grignard reaction of the dibromide **363** first led to the diacetylene **364** in very good yield, and base-catalyzed isomerization as above in Sondheimer's case (Scheme 79) provided in quantitative yield the bisallene **365**, in which the two allene moieties are separated by a 2,3-butadienyl unit. This bisallene is an interesting, highly unsaturated C_10_H_10_-hydrocarbon. On heating, for example, it cycloisomerizes to “tetramethylene benzene”, **366**, an unusual diradical that may be intercepted by double-bond dienophiles, such as fumarates, to furnish the 2:1 adduct **367** and the 2:2 product **368**.

### 4. Bisallene-containing ring systems

#### 4.1 Aromatic bisallenes

A relatively large number of compounds have been described in the chemical literature in which an aromatic system is connected to two (and sometimes more) allene moieties. It is not the purpose of this review to present all of these derivatives, but several typical examples will be discussed.

**Scheme 85:**
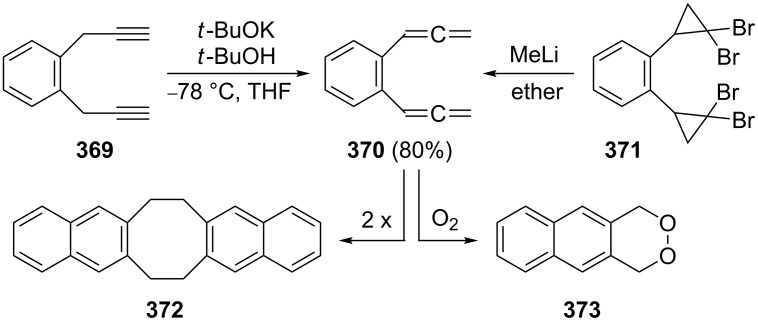
Preparation of 1,2-bis(propadienyl)benzene (**370**).

It is not surprising that the preparative methods we have come across several times so far are also extensively employed for the synthesis of aromatic bisallenes. For example, the *ortho*-bisallene **370** was prepared by both the base-catalyzed propargylic isomerization of the corresponding diacetylene **369** and the application of the DMS route to the *ortho*-divinylbenzene adduct **371** (Scheme 85) [201].

Hydrocarbon **370** can be isolated, i.e. it is considerably more stable than the “parent” system **341** (Scheme 79). Like its olefinic version **341** it can be trapped by oxygen to yield the *endo*-peroxide **373** or it dimerizes to a complex mixture of dimers (47%) in which **372** forms one component. The next higher benzologue of **370**, 2,3-bis(propadienyl)naphthalene, has also been prepared [201]. A more recent route to **370** involves the reduction of the appropriate bispropargylic diacetate with samarium diiodide in the presence of Pd(PPh_3_)_4_ [202]. A number of derivatives of **370** are known that play an important role in the studies of Braverman and co-workers on the generation of diradicals from bisallenic precursors (see Section 6, hetero bisallenes).

**Scheme 86:**
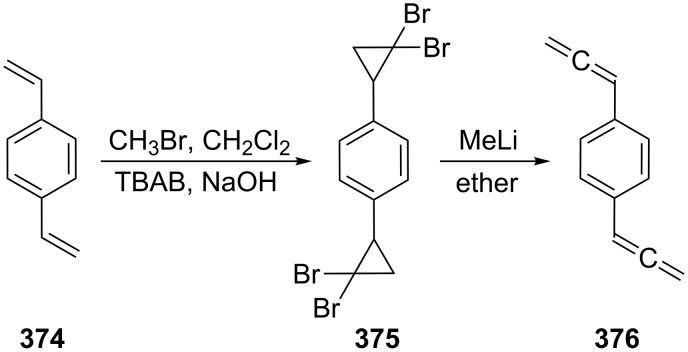
The preparation of 1,4-bis(propadienyl)benzene (**376**).

1,4-Bis(propadienyl)benzene (**376**) and several of its derivatives have recently attracted the attention of a number of research groups. The parent hydrocarbon can be obtained easily by the DMS protocol from *para*-divinylbenzene (**374**) employing phase-transfer catalysis for the preparation of the tetrabromide intermediate **375** (Scheme 86) [203–206].

**Scheme 87:**
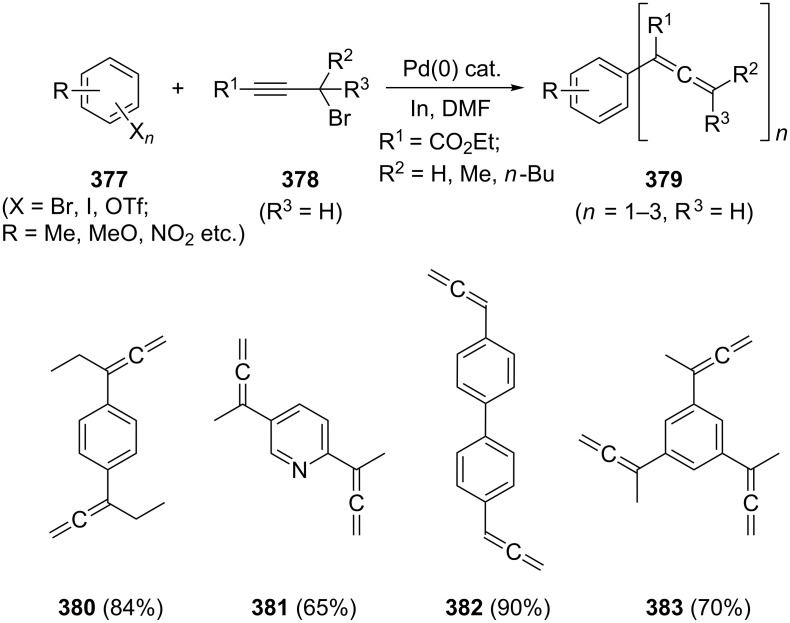
The preparation of aromatic and heteroaromatic bisallenes by metal-mediated coupling reactions.

More often, though, modern metal-mediated coupling reactions are employed to prepare derivatives of **376**. A general route involves different aromatic derivatives **377** and cross coupling of them with propargyl bromides **378** in the presence of Pd(0)/In to provide the aromatic polyallenes **379**, as illustrated in Scheme 87 [186,207]. A selection of allenic coupling products, **380**–**383**, all produced in very good yields, is also shown in Scheme 87. 1,4-Bis(propadienyl)benzene derivatives with varying functional groups (i.e. ester or ether functions) have been prepared by using propargylic carboxylates as substrates [208–209].

**Scheme 88:**
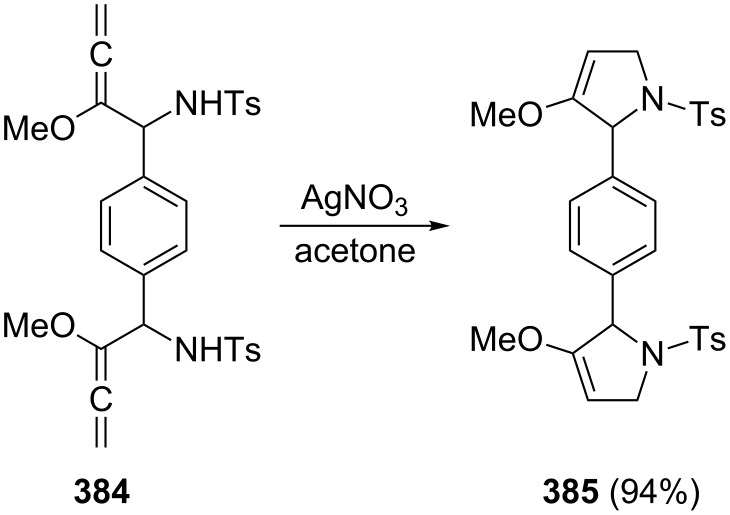
Double cyclization of an aromatic bisallene.

Preparatively, the 1,4-bis(propadienyl)benzenes and their derivatives are of interest for several reasons. Reissig and co-workers demonstrated, for example, that the 1,4-bisallene **384** can be doubly cyclized in quantitative yield to the 3-pyrroline derivative **385** by stirring the allene precursor with silver nitrate in acetone (Scheme 88) [210].

**Scheme 89:**
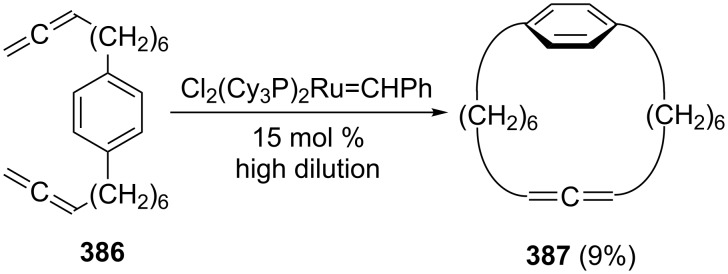
Preparation of an allenic [15]paracyclophane by a ring-closing metathesis reaction of an aromatic α,ω-bisallene.

Even if the allene groups are removed very far from the aromatic core, interaction between them can be induced as illustrated by the ring-closing metathesis reaction of **386**. This hydrocarbon cyclizes in the presence of a Grubbs type I catalyst to the [15]paracyclophane **387** under high-dilution conditions (Scheme 89) [211]. We have noted a similar process already in Section 2.4 (Scheme 77) employing a completely saturated tether (but containing two carbonyl groups) between the allene groups.

**Scheme 90:**
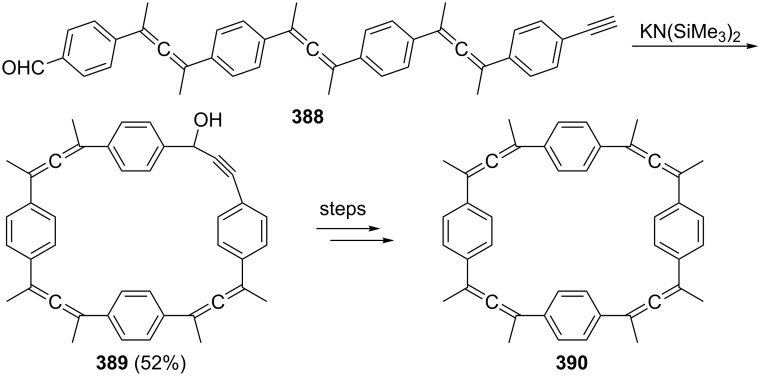
Preparation of a macrocyclic ring system containing 1,4-bis(propadienyl)benzene units.

A more complex case, reported by Krause, Vögtle and co-workers, concerns the trisallene **388**, which contains the 1,4-bis(propadienyl)benzene motive twice; it was assembled from smaller building blocks and connected to the macrocyclic alcohol **389** by treatment with KN(TMS)_2_ (Scheme 90) [212]. Manipulation of the propargyl alcohol section of **389** by standard methods ultimately provided the [3_4_]allenophane **390** [213].

**Scheme 91:**
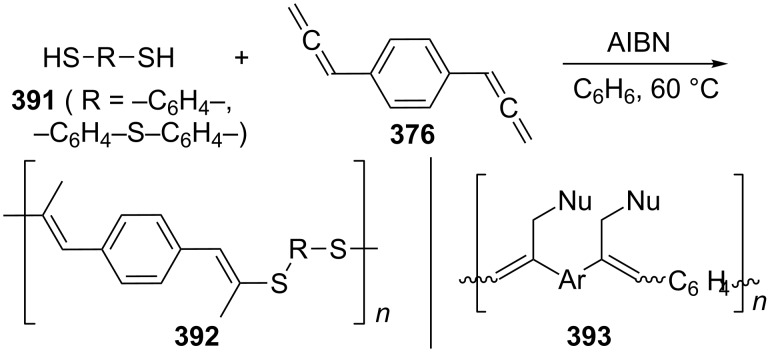
Preparation of copolymers from 1,4-bis(propadienyl)benzene (**376**).

Finally, aromatic bisallenes are interesting substrates for the preparation of new polymeric materials. For example, the disulfides **391** have been copolymerized with 1,4-bis(propadienyl)benzene (**376**) to yield the soluble conjugated polymer **392** (Scheme 91) [193]. And a Pd-catalyzed three-component coupling polymerization of **376** and various difunctionalized aromatics in the presence of nucleophiles (Nu^−^) yielded polymer **393**, a new type of substituted poly(arylene-vinylene) [214].

**Scheme 92:**
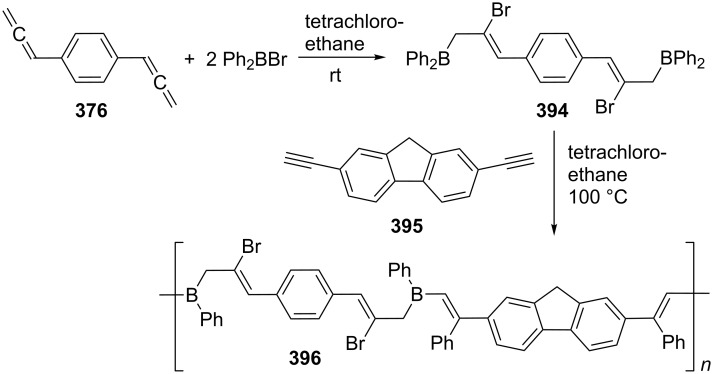
A boration/copolymerization sequence of an aromatic bisallene and an aromatic bisacetylene.

An alternating boration/copolymerization between **376** and 2,7-diethynylfluorene (**395**) was realized by first carrying out a selective haloboration of **376** and then copolymerizing the generated intermediate **394** with **395** (Scheme 92) [189]. When the copolymer **396** was doped with base, it was converted into a polyanion, which may possess σ/π-conjugated-polymer character.

The number of bisallenes in which the allene units are connected to a heteroaromatic ring system seems to be very small [215].

**Scheme 93:**
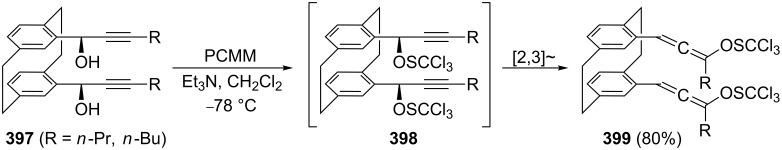
Formation of a layered aromatic bisallene.

In closing we note that a new class of aromatic bisallenes has recently been reported in which the allene units are not bonded to a planar aromatic compound but to a layered one. The case in point is provided by the bisallene **399**, which has been prepared from the [2.2]paracyclophane diol **397**. When this is treated with perchloromethylmercaptane (PCMM) in dichloromethane at low temperature, the bispropargyl intermediate **398** is formed first, which subsequently undergoes a rapid [2.3] sigmatropic shift to **399** in very good yield (Scheme 93) [216–217].

#### 4.2 Bisallenes connected by cyclic spacer groups

There is a countless number of ways by which two (and occasionally more) allene groups may be connected by saturated ring systems and some of these have been discussed in the Introduction already (see above). Here we will begin our review with those compounds in which the two allene moieties are anchored in the ring system in semicyclic fashion. We start with these derivatives for historical reasons and because of their structural simplicity.

**4.2.1 Semicyclic bisallenes**

**Figure 4:**
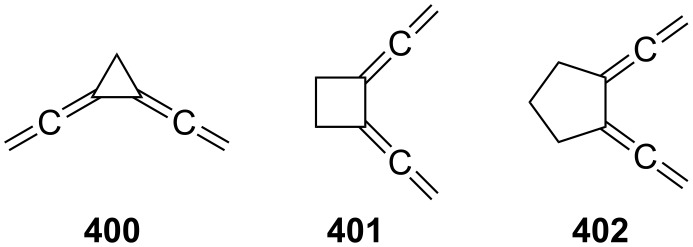
The first members of the semicyclic bisallene series.

The simplest semicyclic bisallene is hydrocarbon **400**, bis(vinylidene)cyclopropane (Figure 4). Taking this compound as a starting point, we will discuss the homologous series with **401** and **402** as the next members in the series. From **401** onwards, the possibility of structural isomers exists, since the allene groups may be positioned in a neighboring or any other arrangement. Whereas **400** apparently has not been synthesized yet, and its heterorganic variants in which the ring methylene group has been replaced by oxygen, sulfur or nitrogen functions are also unknown, the four-membered ring compound **401** and its derivatives have been known and studied for a while.

**Scheme 94:**
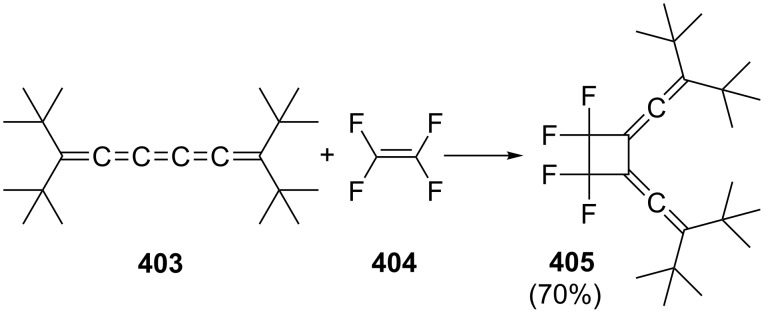
Preparation of the first bis(vinylidene)cyclobutane derivative.

First derivatives of **401** were obtained by Hartzler in his classical studies on the higher cumulenes [218–219]. Having prepared the fully *tert*-butylated hexapentaene **403** for the first time he studied, among other things, its [2 + 2] cycloadditions with activated double and triple bonds. The addition of tetrafluoroethylene (**404**) resulted in the formation of the bis(vinylidene)cyclobutane **405** (Scheme 94). Reactive olefins undergo cycloaddition only to the central double bond of **403**. Interestingly, ethylene itself did not react with the cumulene; in a corresponding experiment (200 °C, EtOAc) only the starting material **403** was isolated at the end of the process.

**Scheme 95:**
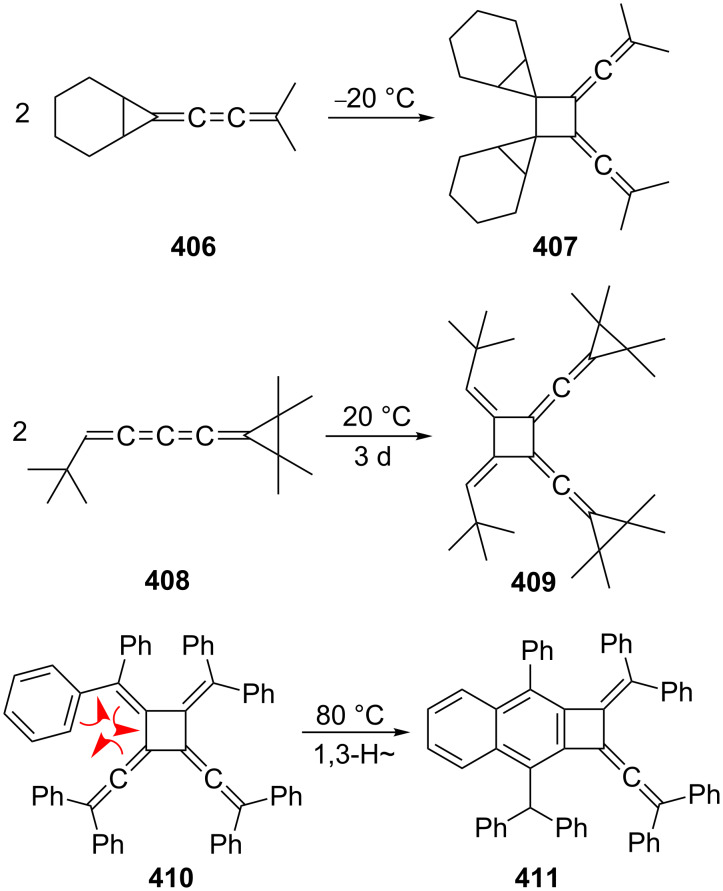
Dimerization of strain-activated cumulenes to bis(vinylidene)cyclobutanes.

In their studies on cumulenic carbenes, Stang and co-workers observed that the [3]cumulene **406** dimerized spontaneously at −20 °C to the bisallenic spiro compound **407**, whose structure as a *syn*-head-to-head dimer was established by an X-ray structural study. Analogously, the pentatetraene **408** leads to **409** quantitatively when left at room temperature for three days (Scheme 95) [220–222]. It is likely that a part of the driving force of these dimerizations is provided by the strain of the three-membered ring. Another derivative of type **409** is produced when tetraphenylpentatetraene is thermally dimerized: the resulting hydrocarbon **410** undergoes an interesting thermal rearrangement to **411** when heated to 80 °C [223].

**Scheme 96:**
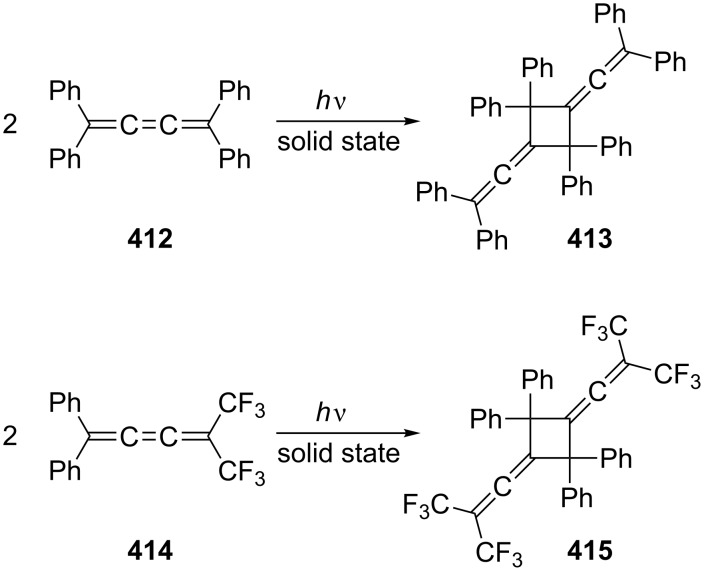
Photodimerization of two fully substituted butatrienes in the solid state.

For compounds such as **407** and **409** there always exists in principle the question of whether these products are head-to-head (1,2-vinylidene) or head-to-tail (1,3-vinylidene) dimers. Additionally, it could be the central double bond of the substrate which reacts, giving rise to the formation of a [4]radialene [224–225]. Whereas the above dimerizations are clearly of the 1,2-type, 1,3-dimers have also been described. Thus, both tetraphenyl-1,2,3-butatriene (**412**) and 1,1-diphenyl-4,4-bis(trifluoromethyl)-1,2,3-butatriene (**414**) photodimerize in the solid state to the 1,3-dimers **413** and **415**, respectively (Scheme 96) [226–227].

**Scheme 97:**
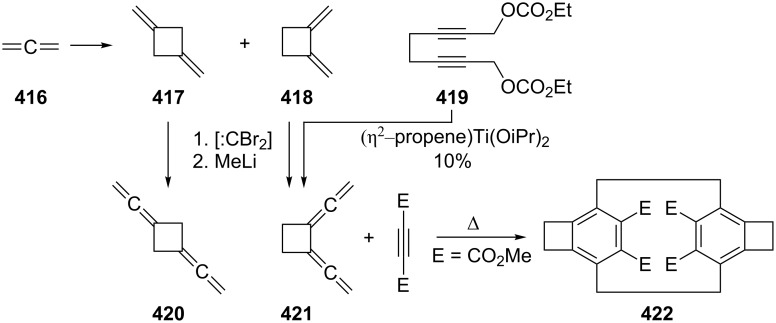
Preparation of the two parent bis(vinylidene)cyclobutanes.

The two parent hydrocarbons 1,2- and 1,3-bis(vinylidene)cyclobutane, **421** and **420** respectively, were eventually synthesized by first thermally dimerizing allene, **416**, to a mixture of 1,2-bismethylenecyclobutane (**418**, main product) and its 1,3-isomer, **421**, and then subjecting this mixture to the DMS allenation protocol (Scheme 97) [228]. The two hydrocarbons can be distinguished by their spectroscopic data and, additionally, by a cycloaddition experiment with dimethyl acetylenedicarboxylate: only the 1,2-dimer **421** affords a cycloadduct, the benzocyclobutenophane **422**. More recently it was suggested that **421** can also be generated from the biscarbonate **419** by treating it with (η^2^-propene)Ti(OiPr)_2_; the yield of the bisallene is poor, though, and it has been identified only in situ by ^1^H NMR spectroscopy [229]. We will return to this method in the context of higher homologues of **421** (Scheme 99).

**Scheme 98:**
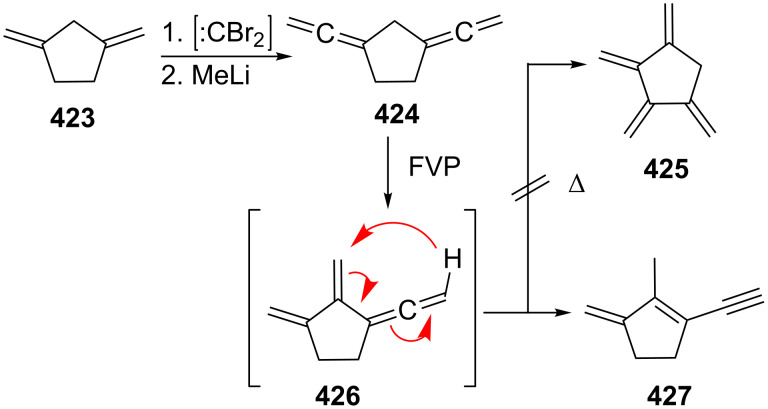
The preparation of 1,3-bis(vinylidene)cyclopentane and its thermal isomerization.

Both isomers of bis(vinylidene)cyclopentane have been prepared: the 1,2-isomer by application of the route just discussed (isolated yield: 45%) [229] and the 1,3-isomer **424** by the DMS method from 1,3-bismethylenecyclopentane (**423**) (Scheme 98) [230]. The intended thermal isomerization of **424** to **425** by flash vacuum pyrolysis could not be realized. Rather than yielding this “nor[5]radialene” the pyrolysis provided the dienyne **427**, presumably via the “half-isomerized” intermediate **426** by a symmetry-allowed 1,5-hydrogen shift.

**Scheme 99:**
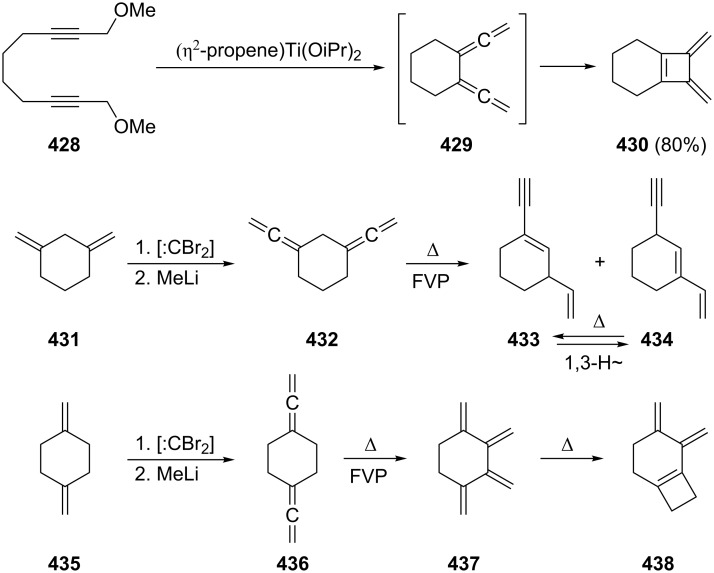
The preparation of the isomeric bis(vinylidene)cyclohexanes.

Proceeding to the corresponding bis(vinylidene)cyclohexanes, we note that three isomers are conceivable now and that the problem of preparing all three has been addressed in the chemical literature already (Scheme 99) [230]. However, when the Sato method was applied to the bisether **428**, only the bicyclic bismethylenecyclobutene **430** resulted, although in very good yield. A similar result was observed for the next higher homologue, 1,2-bis(vinylidene)cycloheptane. Evidently, the particular arrangement of the allene units in the relatively unstrained ring of **429** makes interaction between them particularly favorable. In the cases of the 1,3 and 1,4-isomers, **432** and **436** respectively, their preparation from the starting dienes **431** and **435** posed no problems. Whereas the thermal isomerization of **432** caused isomerization to a mixture of the dienynes **433** and **434**, which are themselves in thermal equilibrium by a 1,3-hydrogen shift, the thermal rearrangement of **436** indeed provided a 1,2,3,4-tetramethylenecyclohexane (**437**), formally a 2,5-bridged [4]dendralene [48]. The latter hydrocarbon is in thermal equilibrium with the cyclobutene isomer **438**, a bicyclic [3]dendralene. The structure of both products was established by spectroscopic and chemical means. The thermal isomerization of **436** also sheds some light on the rearrangement of 1,5,9-cyclododecatriyne into [6]radialene, as will be discussed in Section 5 (Scheme 113) [231].

**Scheme 100:**
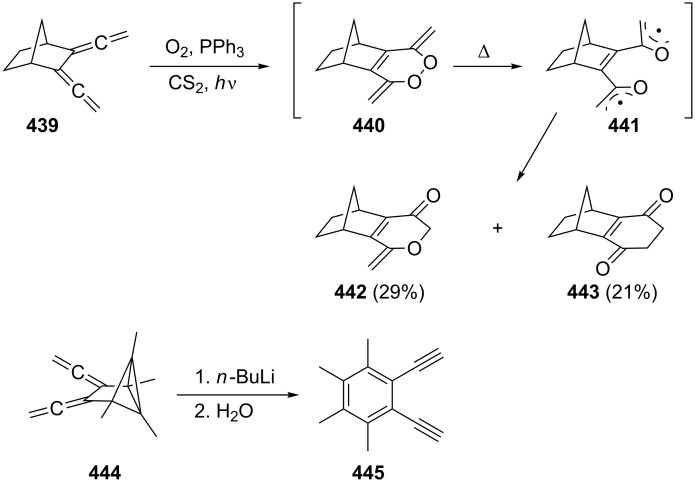
Bi- and tricyclic conjugated bisallenes.

More complex oligocyclic spacer groups have been inserted between the allene moieties as shown by the examples in Scheme 100. Both **439** and **444** were obtained from the corresponding dienes by the DMS route (see Section 1.1). In an attempt to prepare a stable divinylperoxide, **440**, Schuster and Mebane [232] added singlet oxygen to the bicyclic bisallene **439**. However, rather than isolating the expected adduct **440**, the two rearrangement products **442** and **443** were obtained. The process probably involves the diradical intermediate **441**, generated by the homolysis of the oxygen–oxygen bond under the reaction conditions. With TCNE, on the other hand, **439** provided the corresponding [2 + 4] cycloadduct. The bisallene **444** was prepared by Hogeveen and Heldeweg and produced the expected Diels–Alder adduct with TCNE. They noted that under the conditions of its preparation from the corresponding dichlorocarbene bis-adduct, the diethynylbenzene derivative **445**, an aromatic isomer of **444**, is also produced as a secondary product [233].

**Scheme 101:**
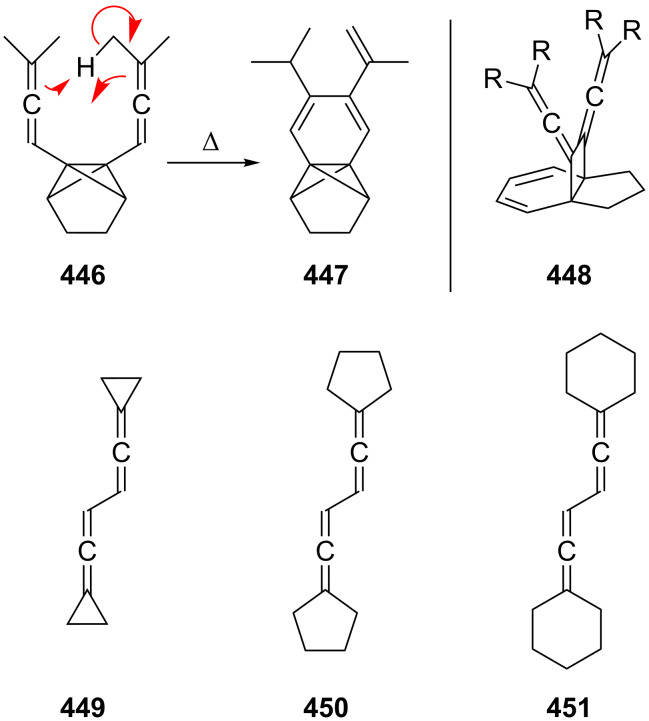
A selection of polycyclic bisallenes.

That these highly strained hydrocarbons are prone to reduce their angular strain by undergoing rearrangement processes is also underlined by an example of Hashmi and Szeimies who reported that the [1.1.0]bicyclobutane derivative **446** cannot be isolated and participates in an intramolecular ene reaction to provide the more stable isomer **447** (Scheme 101) [234].

In closing this section we note that many other types of semicyclic bisallenes can be designed on the drawing board that should possess interesting structural and chemical properties including the (still unknown) cyclopropane derivative **449**. Compounds of this general type, in which the three-membered rings are replaced by five- and six-membered rings, **450** and **451** respectively, have been prepared though [42,58–64]. The four-membered analogue of **449** is also unknown. Another semicyclic bisallene of type **439**/**444**, the fully substituted derivative **448** was obtained by Tobe et al. as a side product in a study concerned with the preparation of cyclo[*n*]carbons by cycloreversion reactions from propellane-annelated dehydro[*n*]annulenes [235].

**4.2.2 Endocyclic bisallenes**

In their simplest form endocyclic bisallenes are those compounds that contain both allene units in one ring system, as represented by structure **15** in Figure 3. The allene units may be directly bonded to each other (conjugated) or nonconjugated (positioned in any section of the ring system). A sizable number of these compounds have been prepared during the past few decades and studied especially from the structural viewpoint.

**Scheme 102:**
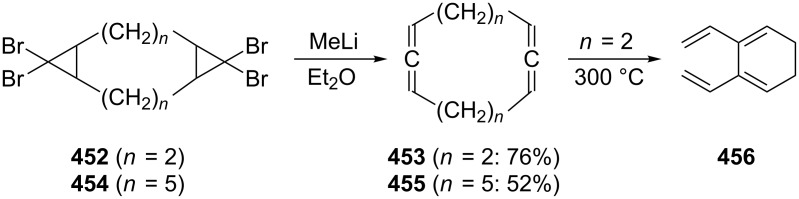
The first endocyclic bisallenes.

The first author to investigate endocyclic, alicyclic bisallenes apparently was Skattebøl, who, in 1961, prepared hydrocarbons **453** and **455** from the respective precursors **452** and **454** by methyllithium treatment (Scheme 102) [236]. Both hydrocarbons were obtained in good (isolated) yields. Since any 1,3-disubstituted allene is chiral, the products **453** and **455** should have been formed as mixtures of diastereomers, a *meso*- and a *d*,*l*-form. However, the first publication did not address this question. It was, on the other hand, soon discovered that **453** undergoes a thermal isomerization to 2,3-divinyl-1,3-cyclohexadiene (**456**), a bridged [4]dendralene [48], which is produced in virtually quantitative yield [237].

**Figure 5:**
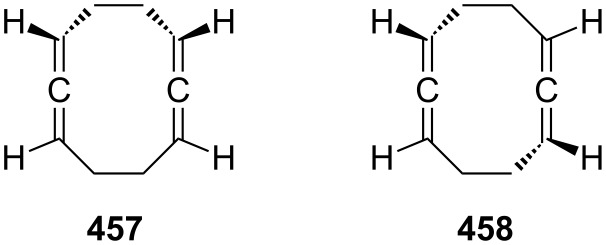
The stereochemistry of 1,2,6,7-cyclodecatetraene.

From that time, the early 1960s, the questions of the detailed stereostructures of endocyclic bisallenes and their reactivity, particularly the behavior of **453**, have interested many authors. Dehmlow and Stiehm later performed a thorough stereochemical analysis of the tetrabromides **452** (and their dibromide precursors) and suggested that **453** has the *meso*-, **457**, and not the *d*,*l*-configuration, **458** (Figure 5) [238–239]. This suggestion was confirmed later by an X-ray structural analysis by Irngartinger [240] and a thorough dynamic NMR analysis (DNMR) by Anet and co-workers [241]. According to Anet's analysis, the lowest energy conformation of **453** has *C_i_* symmetry. Symmetrization of this conformation (**457**) results in a *C*_2_*_h_* time-average symmetry; the experimentally determined barrier of this conformational change by DNMR spectroscopy leads to a Δ*G*^‡^ of 7.1 kcal/mol. The ground state of **458** is found by force-field calculations to possess a “nonintersecting” twofold axis of symmetry [242]. Apparently the *d*,*l*-diastereomer **458** is unknown until the present day.

**Scheme 103:**
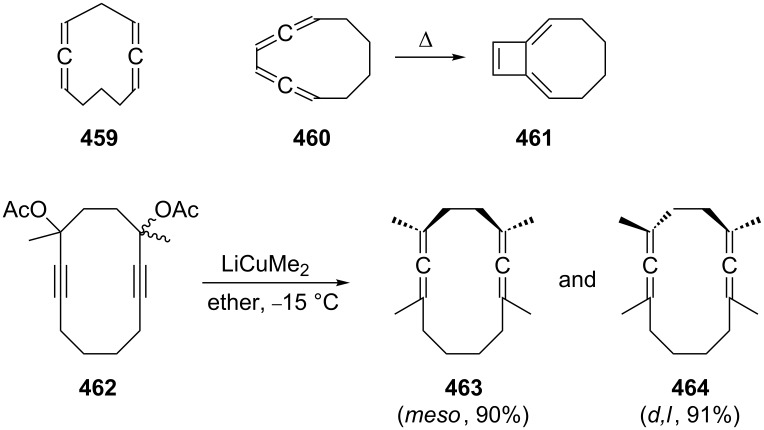
The preparation of several endocyclic bisallenes.

A number of other cyclobisallenes have been described in which the connecting bridges are of different length; most of these hydrocarbons were obtained by application of the DMS protocol to the corresponding dienes. Scheme 103 collects some of these compounds. Whereas the isomer **459** of the above decatetraene **453** could be isolated, its isomeric hydrocarbon **460** valence tautomerized to the bridged bismethylenecyclobutene **461** (for the bisallene to bismethylenecyclobutene isomerization of acyclic bisallenes, see Section 1.4.1) [243]. In a (rare) route not involving the DMS synthesis, Keese and Boss subjected propargylic acetates, such as **462**, to a double methylation reaction with LiCuMe_2_ in ether at −15 °C (Scheme 103). Depending on the configuration of the substrate **462** (*meso* or *d*,*l*) the *meso*-bisallene **463** or its *d*,*l*-diastereomer **464** were obtained in excellent yields. This route can also be applied to the preparation of other derivatives of 1,2,6,7-cyclododecatetraene [155].

**Scheme 104:**
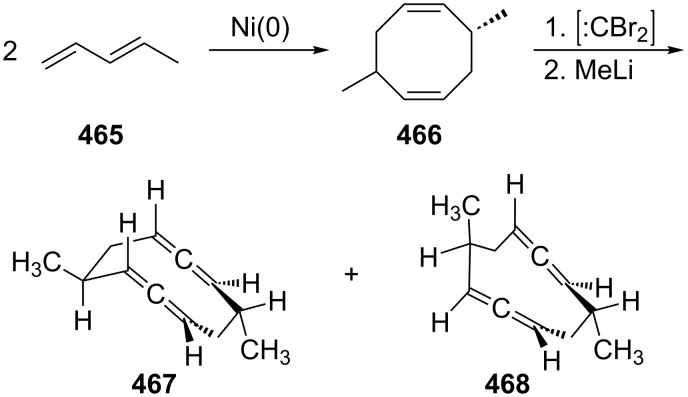
Synthesis of diastereomeric derivatives of 1,2,6,7-cyclodecatetraene.

To prepare the *meso*- (**467**) and the *d*,*l*-diastereomers (**468**) of the 4,9-dimethyl derivatives of 1,2,6,7-cyclodecatetraene **453**, Roth and co-workers first catalytically dimerized (*E*)-1,3-pentadiene (**465**) to the 1,5-cyclooctadiene derivative **466** which, on DMS treatment, provided a mixture of the two stereoisomers in 70:30 ratio (Scheme 104) [244]. The two compounds could be separated by gas chromatography; if the cyclopropylidene-to-allene conversion was carried out in the presence of spartein, **468** was obtained in optically active form. On pyrolysis (300 °C) the above conversion to 2,3-divinyl-1,3-cyclohexadiene derivatives (and their follow-up products) took place. It was concluded from trapping experiments and a stereochemical analysis [245–248] that the *meso*-compound isomerizes by a concerted Cope-type rearrangement via a diradical intermediate, whereas the pyrolysis of the *d*,*l*-compound involves two competing reactions, a concerted and a nonconcerted one. The different behavior of the two diastereomers was traced back to the boat and chair geometries of the respective transition states.

**Scheme 105:**
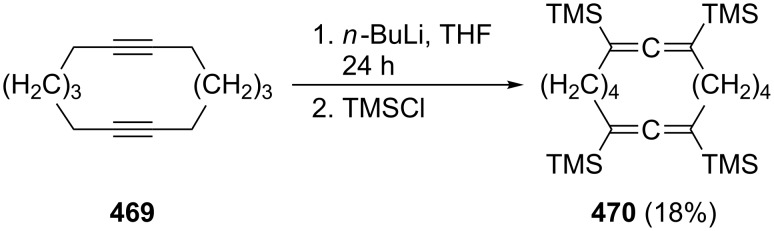
Preparation of a derivative of 1,2,8,9-cyclotetradecatetraene.

A number of functionalized derivatives of cyclic bisallenes have also been described. For example, as demonstrated in Scheme 105, on metalating 1,8-cyclotetradecadiyne (**469**), with *n*-butyllithium in THF, a polyanion is generated, which, after quenching with TMSCl, furnishes three products, among them the cyclic bisallene derivative **470** [249].

**Scheme 106:**
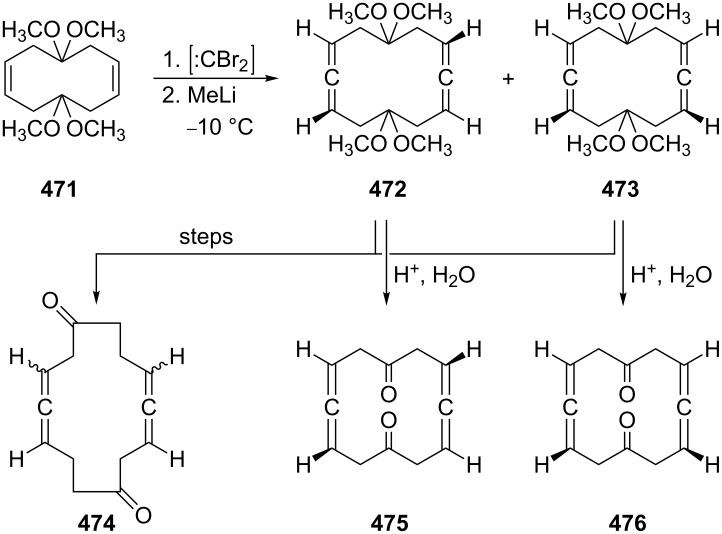
The preparation of keto derivatives of cyclic bisallenes.

A number of derivatives are known in which the usually saturated part of a cyclic bisallene (see **15**, Figure 3) carries a functional group. For example, the Sondheimer group has obtained the two diketones **475** and **476** by first preparing the diketals **472** and **473** from the corresponding diolefin **471** by the DMS route and then hydrolyzing them to the target molecules (Scheme 106) [250–251]. When the allene synthesis was carried out in the presence of (−)-sparteine the two ketals were obtained in optically active, **472**, and inactive form, **473**. The two diastereomers could be separated by column chromatography on silica gel and subsequent hydrolysis yielded the optically active diketone (+)-**475** and its inactive stereoisomer **476** (*meso*) [252]. In several preparative applications of both **472**/**473** and **475**/**476** the Sondheimer and Garrat groups exploited the potential of these cyclic bisallenes for further ring expansions. Thus, by several steps the ketals were converted into the fourteen-membered allenic ketones **474**, which, again, were obtained as *d*,*l*- and *meso*-diastereomers [252].

**Scheme 107:**
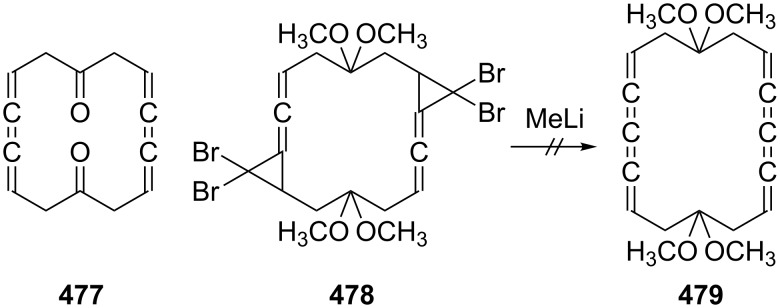
The preparation of cyclic biscumulenic ring systems.

Later studies saw the use of these diketals for the preparation of even more extended cumulenic systems. Whereas the monocyclic dicumulenic dione **477** could be prepared by application of the above protocol [253], the attempted preparation of 3,4,5,6,11,12,13,14-cyclohexadecaoctaen-1,9-dione from the cyclic bis-allenic precursor **479** failed, since this bisketal could not be obtained from its precursor **478** (Scheme 107) [254–255].

**Scheme 108:**
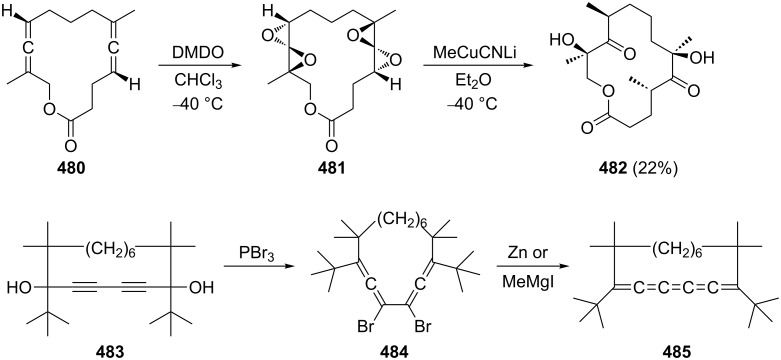
Cyclic bisallenes in natural- and non-natural-product chemistry.

We conclude this section on cyclic bisallenes by making reference to two functionalized systems of this general type and their use for the preparation of large ring systems. In Scheme 108 it is shown how a compound of type **329** (Scheme 76) was used by Williams and co-workers to develop the synthetic methodology for the efficient preparation of various compounds related to erythronolide A [190,256]. Model system **480** was first oxidized with dimethyldioxirane (DMDO), and the resulting bis(spiro)diepoxide derivative **481** was subsequently opened by a nucleophile (a cuprate in this case) to **482**. Although the yield of 22% is not high, it is sufficient considering what has been accomplished from the view of structural complexity. The second example involves the macrocycle **484**. For its preparation the bispropargylic alcohol **483** (itself obtained from the appropriate precursor by a Glaser coupling procedure) is treated with PBr_3_ (Scheme 108) [60]. On reduction of **484**, which itself is one of the rare 3,4-dihalo-diallenes (see above, Section 1.2, Scheme 15) with zinc powder, the [5]cumulene **485** is produced. Other bridging elements than the shown polymethylene chain may be introduced to provide comparable ring systems in corresponding yields. The structure of **484** was established by X-ray structural analysis with the two bromine substituents in a *transoid* arrangement.

**Scheme 109:**
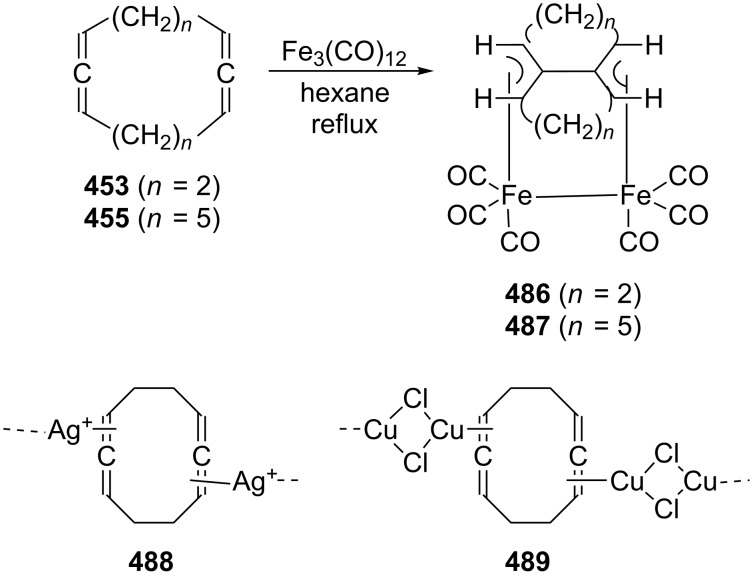
The preparation of iron carbonyl complexes from cyclic bisallenes.

Cyclic bisallenes belong to the few compounds of this class of unsaturated systems for which metal complexes have been reported. Thus both 1,2,6,7-cyclodecatetraene (*n* = 2, **453**) and 1,2,9,10-cyclohexadecatetraene (*n* = 5, **455**) on heating with Fe_3_(CO)_12_ in boiling hexane yield red-orange bicyclic oligomethylene ethane diiron hexacarbonyl complexes. The structures of **486** and **487** have been derived from spectroscopic data and X-ray structural analyses (Scheme 109) [257]. Furthermore, **453** has been shown to form well-defined crystalline π-complexes with silver(I) nitrate and copper(I) chloride. According to spectroscopic and analytical data these metal complexes are of polymeric nature and possess structures **488** and **489** (Scheme 109) [258].

**4.2.3 Exocyclic bisallenes**

**Figure 6:**
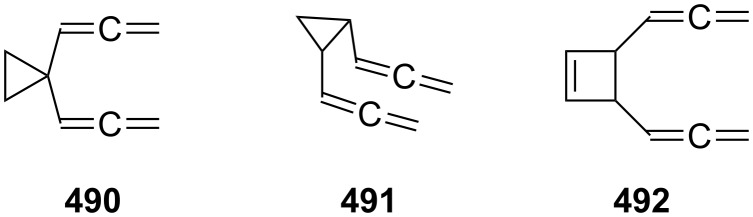
A selection of unknown exocyclic bisallenes that should have interesting chemical properties.

Bisallenes in which the two allene groups are outside of an alicyclic ring system (i.e., exocyclic) have hardly been described in the chemical literature. Usually the ring systems in these derivatives are of aromatic nature (see Section 4.1). Many such systems with interesting chemical properties can be imagined; Figure 6 displays just three of them, the hydrocarbons **490**–**492**. Any of these (and many more) should display interesting chemical behavior in, e.g., isomerization and addition reactions.

### 5. Bisallenes as reactive intermediates

We have already mentioned several bisallenes as reactive intermediates that easily undergo cycloadditions and/or rearrangement reactions, see for example Scheme 55 (**229**), Scheme 79 (**341**) and Scheme 97 (**421**). Nevertheless we would like to return to this topic in a separate chapter since bisallenes have been suggested as reactive intermediates in some very interesting (multistep) transformations. In none of these examples could the bisallenic intermediates actually be isolated or directly observed.

**Scheme 110:**
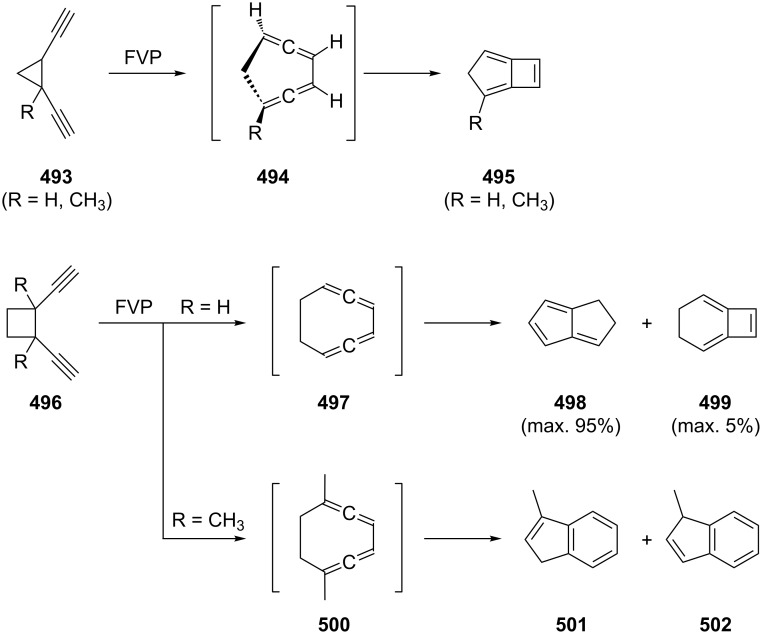
The thermal isomerization of 1,2-diethynylcyclopropanes and -cyclobutanes.

In their studies on the thermal isomerization of *cis*- and *trans*-1,2-diethynylcyclopropanes **493**, Bergman and co-workers postulated the generation of 1,2,4,5-cycloheptatetraenes **494**, as reaction intermediates, produced from **493** by a Cope-type isomerization (see Section 1.4.1). These highly strained cyclic bisallenes, which according to quantum chemical calculations prefer the *d*,*l*-configuration, immediately cycloisomerize to the bicyclo[3.2.0]heptatrienes **495** (Scheme 110) [259–261]. The next higher homologue of **493**, *cis*- and *trans*-1,2-diethynylcyclobutane (**496**, R = H) was studied by Eisenhuth and Hopf [262–263]. Although in this case the initially generated, highly strained intermediate **497** still cyclizes to the bismethylenecyclobutene product, **499**, its by far preferred mode of stabilization involves a 1,5-bridging step and insertion of the thus produced carbene intermediate into the neighboring C,H-bond to produce 1,2-dihydropentalene **498**, in high yield. If the α-position is blocked by a methyl substituent, **496** (R = CH_3_), the thermal isomerization of the bisacetylenic substrate initially provides the expected cyclic bisallene **500**, but then takes a different course, furnishing, among other things, the indene derivatives **501** and **502**.

**Scheme 111:**
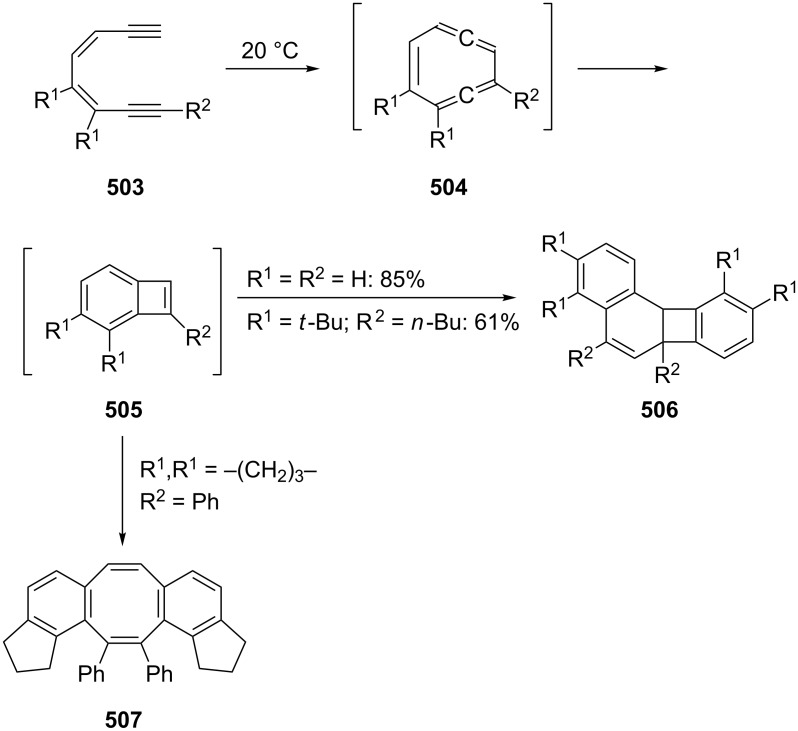
Intermediate generation of a cyclooctapentaene.

The dehydro analogue of **497**, 1,2,4,6,7-cyclooctapentaene (**504**, R^1^ = R^2^ =H) was suggested by Sondheimer and Mitchell to be produced from 3,5-octadien-1,7-diyne (**503**, R^1^ = R^2^ = H) at room temperature (Scheme 111) [264]. The monocyclic bisallene isomerizes immediately to the benzocyclobutadiene **505**, which produces a stable dimer, the hydrocarbon **506**, in good yield. Derivatives of **503** with, e.g., cycloalkyl and phenyl substituents behave analogously up to the benzocyclobutadiene **505** but subsequently dimerize to cyclooctatetraene **507** via an intermediate [3]ladderane [265].

**Scheme 112:**
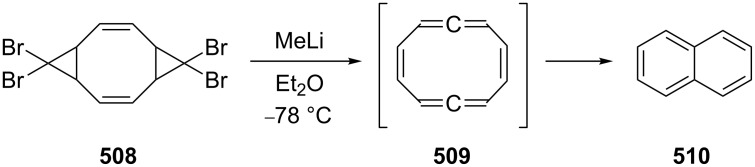
Attempted preparation of a cyclodecahexaene.

In an important experiment Dehmlow and Ezimora prepared the dibromocarbene bis-adduct of cyclooctatetraene **508** and treated it with methyllithium in diethylether at −78 °C. Rather than the expected 1,2,4,6,7,9-cyclodecahexaene (**509**), they isolated naphthalene (**510**) as the sole reaction product (Scheme 112) [238–239].

**Scheme 113:**
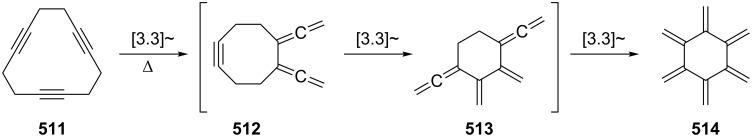
The thermal isomerization of 1,5,9-cyclododecatriyne (**511**) into [6]radialene (**514**).

In another thermal isomerization experiment, Vollhardt and Dower studied the conversion of 1,5,9-cyclododecatriyne (**511**), into [6]radialene (**514**), a process that in principle could take place via an intramolecular [2 + 2 + 2] cycloaddition to a triscyclobutenobenzene intermediate and ring opening of the latter, or by three consecutive [3.3] sigmatropic shifts involving two semicyclic bisallene intermediates, **512** and **513** (Scheme 113) [266–267]. That this second alternative is indeed the more likely one was demonstrated by introducing a ^13^C-label into the substrate **511** and then following its distribution in the final rearrangement product **514**.

**Scheme 114:**
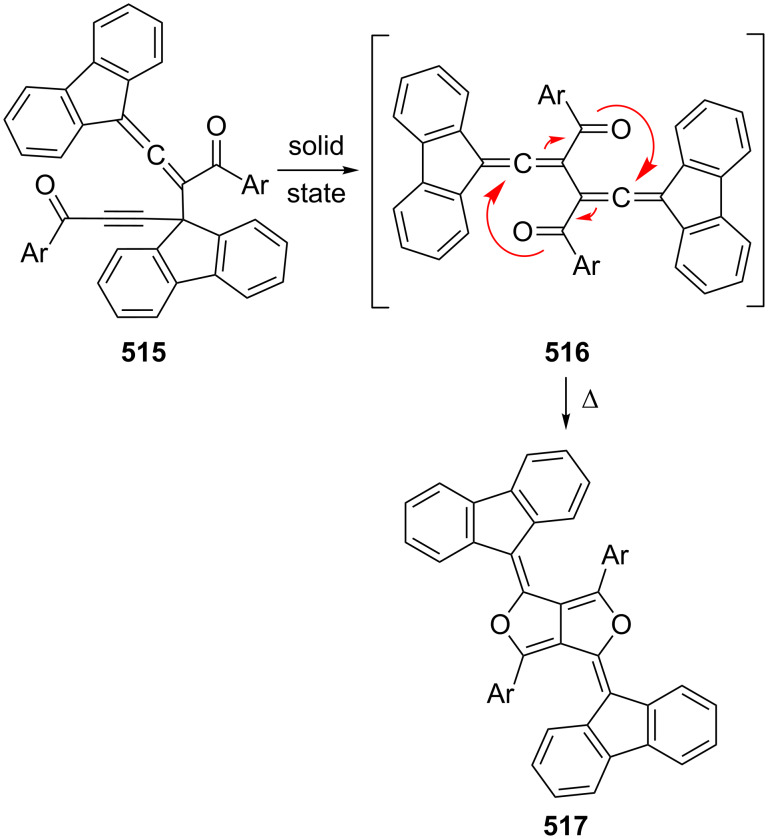
An isomerization involving a diketone derived from a conjugated bisallene.

A solid-state thermal transformation probably involving a functionalized derivative of a conjugated bisallene **516** was described by Tanaka and co-workers (Scheme 114) [268]. Heating the colorless propargylallene derivative **515** at ca. 200 °C causes a color change to copper brown without melting of the substrate crystals. Spectroscopic and X-ray evidence revealed that the furofurane derivative **517** had been produced; a plausible intermediate in this interconversion is the bisallene **516**, which cyclizes as indicated in Scheme 114.

### 6. Bisallenes with heteroatoms in their tethers (heteroorganic bisallenes)

**Scheme 115:**
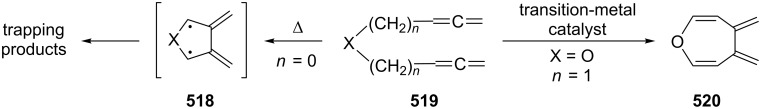
Typical reaction modes of heteroorganic bisallenes.

Bisallenes in which the structural element connecting the two allene moieties is or contains a non-carbon atom (usually S, N and O), form an important and interesting subgroup of this class of organic compounds. Trying to distill a unifying concept out of the vast literature is difficult. One solution may be to group the many results into two categories (Scheme 115).

In the first category we find those reactions of the heterorganic bisallenes **519** that are initiated by a (usually thermal) cycloaddition yielding a diradical intermediate, **518**. Depending on the substituents present in the substrate, the actual reaction conditions and whether, for example, the reaction mixture contains other reaction partners (trapping agents), **518** can subsequently lead to quite diverse products.

In the second category we find transition-metal-initiated or catalyzed processes, which lead to cyclization products often containing medium-sized ring systems, as symbolized by **520**. This reaction mode is particularly characteristic for the 1,5-diallenes already briefly discussed in Section 2.4 for all-carbon systems.

#### 6.1 Thermally induced reactions of heteroorganic bisallenes

**Scheme 116:**
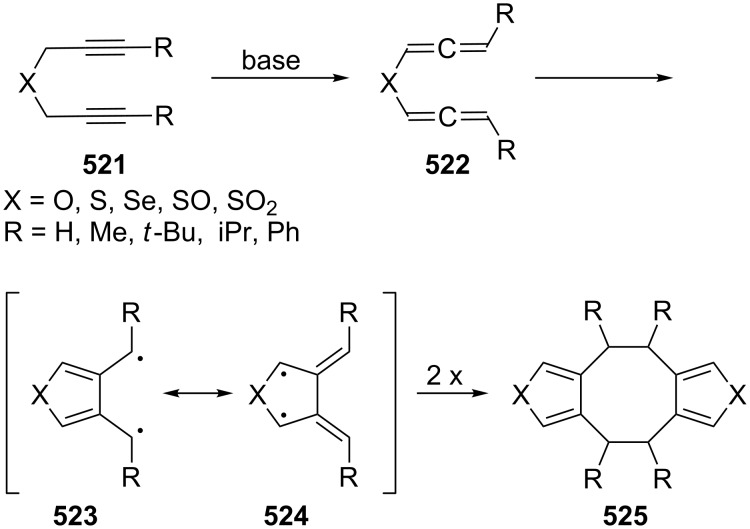
Generation and thermal behavior of acyclic hetero-organic bisallenes.

The generation and chemical behavior of many heterorganic acyclic bisallenes is summarized in Scheme 116 and basically consists of three steps [269]. In the first step the bisallene **522** is generated from a usually readily available diacetylene **521** by base treatment. This reactive species subsequently undergoes C–C bridging to furnish the resonance-stabilized diradical **523**↔**524**. In the terminating step, dimerization to compounds of type **525** often occurs. In practice, the first step can be technically quite difficult, though, requiring often carefully controlled reaction conditions, which, additionally, may differ significantly from one substrate system (**521**) to the other. Quite often the bisallene intermediate is not detected at all, which for the present review would have meant to deal with it in Section 5.

**Scheme 117:**
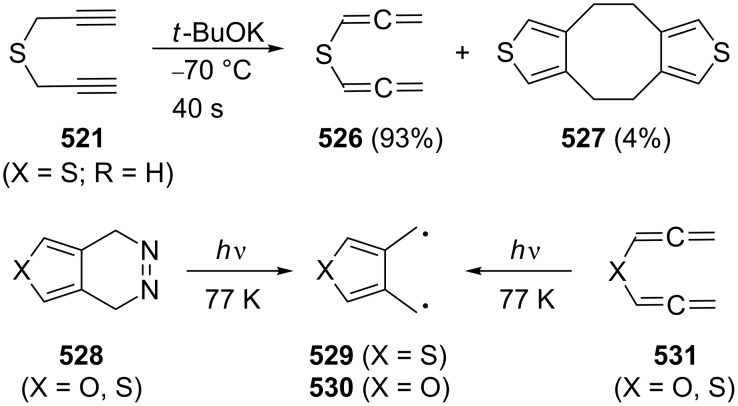
Generation of bis(propadienyl)thioether.

A typical example, studied by Garratt and co-workers, involves the thioether **521** (X = S, R = H) [270–271]. When this diacetylene was treated with *t*-BuOK in THF at −70 °C for 40 s a mixture of **526** (93%), the dimer **527** (4%) and the substrate **521** (4%) resulted (Scheme 117) [270]. However, for the analogous ether **521** (X = O, R = H) harsher reaction conditions were required: the temperature had to be increased to 0 °C and the reaction time extended to 25 min [270–271]. Not surprisingly, when this process was carried out with the thioether in the presence of oxygen as a trapping reagent, the corresponding *endo*-peroxide was isolated. The two diradical intermediates **529** and **530** can also be studied directly by stabilizing them in a matrix at low temperatures (77 K) as demonstrated by Berson and co-workers (Scheme 117) [272–273]. As was shown by these workers using cross-polarization magic-angle spinning (CPMAS) ^13^C NMR spectroscopy, the reaction intermediates possess the diradical structures **529** and **530** and not, e.g., those of a bicyclic full-valence isomer. Both diradicals were generated by matrix photolysis of the precursor bisallenes **531** and by low-temperature photolysis of the corresponding diazenes **528** in these investigations (X = O, S).

**Scheme 118:**
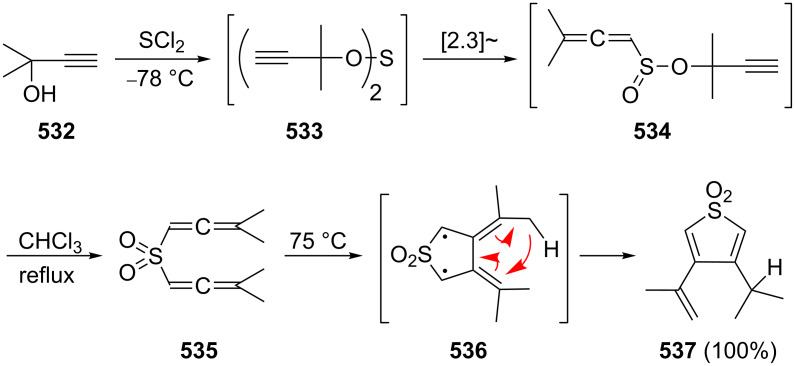
The preparation of a bisallenic sulfone and its thermal isomerization.

Modification of the substitution pattern and/or the heteroorganic bridging element leads to substantial changes in the reaction mechanisms and the preparative outcome of these processes. For example, in a classic paper Braverman and co-workers prepared the diallenic sulfone **535** from 2-methyl-3-butyne-2-ol (**532**) and sulfur dichloride by a double [2.3] sigmatropic rearrangement involving **533** and **534** as reaction intermediates (Scheme 118) [274–275]. On heating of **535** it undergoes quantitative cyclization to the thiophen-1,1-dioxide **537**. Analogues of **535** in which the sulfone group has been substituted by S, Se and O could also be prepared and they undergo the same cycloisomerization, although under milder conditions [276–277]. A mechanistic study of the rearrangement was performed by using deuterated methyl groups in e.g. **535** and studying the label distribution in the products and the isotope effect. From its absence it was concluded that the reaction takes place in two steps, with the first rate-determining step involving ring closure to an intermediate for which structure **536** can be postulated, in analogy to the above studies [274,278].

**Scheme 119:**
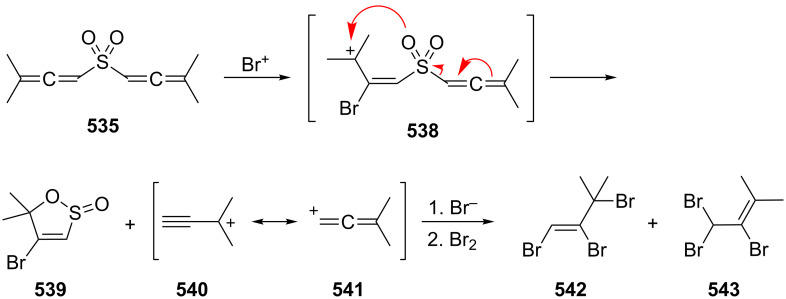
Bromination of the bisallenic sulfone **535**.

The diallenyl sulfone **535** is an interesting substrate in other respects, too. On adding bromine to it at room temperature the compound undergoes an addition reaction accompanied by a fragmentation (Scheme 119) [279]. The initially produced carbocation **538** fragments to **539**, one of the isolated products, and evidently to the resonance-stabilized cation **540**↔**541**, since the bromine trapping products resulting from this species, the tribromides **542** and **543**, can also be isolated.

**Scheme 120:**
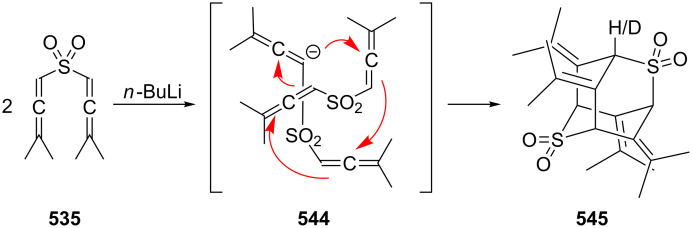
Metalation/hydrolysis of the bisallenic sulfone **535**.

On metalation of **535** with *n*-butyllithium in THF at 0 °C, followed by hydrolysis/deuterolysis a remarkable dimer **545** can be isolated, the adamantanoid structure of which was established by X-ray structural analysis (Scheme 120) [280]. It has been proposed that the monoanion derived from **535** attacks a second molecule of the substrate in the “crossed” fashion shown in **544**. The resulting dimer should possess a deuterium atom in the position shown in the scheme after D_2_O workup, and this is indeed found experimentally.

**Scheme 121:**
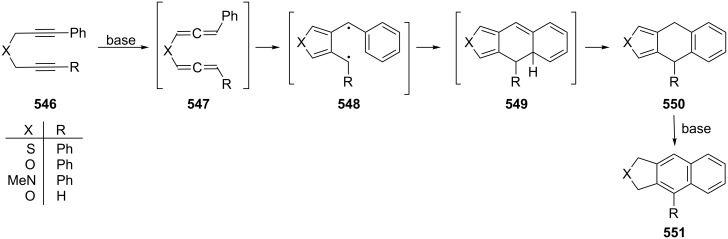
Aromatic compounds from hetero bisallenes.

Taking diyne **521** (see Scheme 116) as the parent system, this can be varied in countless ways, for example by changing the substituents R or by replacing the heteroorganic molecular “bridge” by other combinations of heteroatoms. The corresponding studies have been carried out over a vast range of exchanges/permutations, notably by Braverman and by Garratt and their co-workers. Beginning with the simple phenyl and diphenyl derivatives **546**, Iwai and Ide [281] and later Garratt and Neoh [282] have shown that they isomerize in good yield (40–100%) by base treatment to the naphthalene derivatives **551**. Although several mechanisms have been proposed for this isomerization the one summarized in Scheme 121 seems to be the most likely one. The process begins with the formation of the hetero bisallene **547**, which next undergoes the expected ring closure to **548**. This diradical intermediate, however, carries at least one phenyl substituent, which can participate in the next step, cyclization to **549**. As an “isotoluene” **549** is unstable and isomerizes to the bridged heterocycle **550**. This compound, which can be isolated, finally stabilizes itself by forming the more stable naphthalene isomer **551**.

**Scheme 122:**
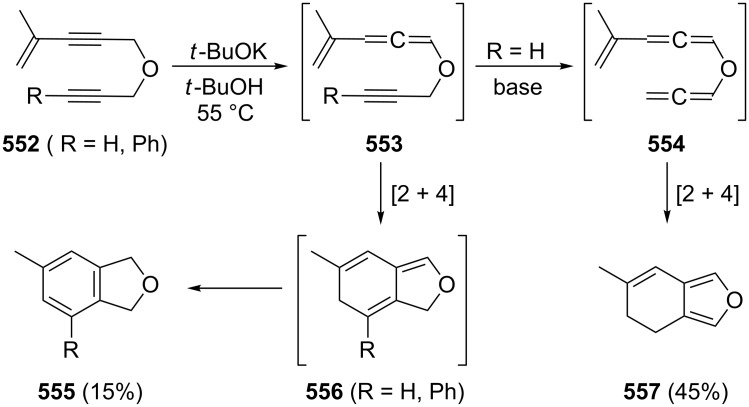
Isomerization/cyclization of bispropargylic ethers.

An analogous mechanism can also be proposed for the isomerization of the vinyl-substituted bispropargyl ether **552**, which has been studied by Ollis et al. (Scheme 122) [283–285]. In this case the initial isomerization leads to the monoallenes **553**, which can either undergo a second acetylene-to-allene rearrangement and provide the bisallenyl ether **554** or participate in a Diels–Alder addition to **556**. Ring closure of **554** as above leads to the furan derivative **557**, whereas **556** stabilizes itself to **555**.

**Scheme 123:**
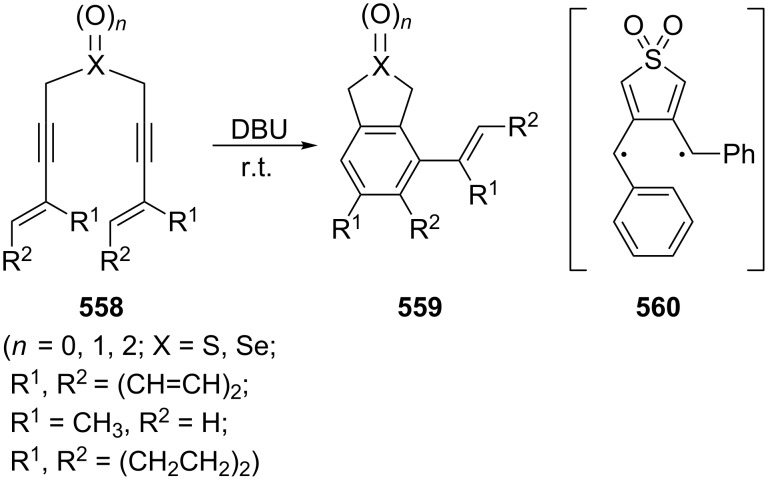
The preparation of novel aromatic systems by base-catalyzed isomerization of bispropargyl ethers.

Braverman et al. generalized these processes and, in particular, extended them to bispropargylic sulfides, selenides, sulfoxides and sulfones, which in the presence of amine bases, such as DBU, undergo facile isomerization to the corresponding bisallenes (which are usually not isolated or even detected by, e.g., NMR spectroscopy) [286–289]. The ensuing tandem cyclization and aromatization reactions are comparable to the ones discussed above. The comprehensive studies of this group can be presented in general form by the conversion of **558** (substrate) into **559**, the product formed by the sequential process discussed above (Scheme 123). Some of the diradicals formed in these processes, such as **560**, possess a high potential for DNA cleavage [286].

**Scheme 124:**
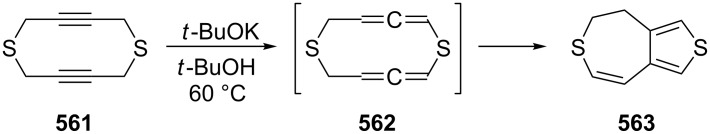
The isomerization of bisacetylenic thioethers to bicyclic thiophenes.

Cyclic variants of these rearrangements have been observed as early as 1964 when Eglinton, Raphael and co-workers isomerized 1,6-dithia-3,8-cyclodecadiyne (**561**) to the bridged thiophene **563**. Presumably, **562** is generated as an intermediate in this transformation and subsequently cyclizes via the corresponding diradical intermediate followed by a hydrogen shift (Scheme 124) [290].

**Scheme 125:**
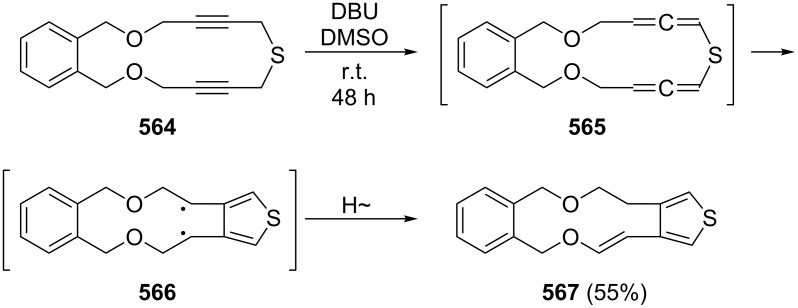
Aromatization of macrocyclic bispropargylic sulfides.

Modern variants involve more complex sulfides such as **564** as well as various selenides; these derivatives have been investigated by the Braverman group (Scheme 125) [291]. The formation of the product **567** can be explained readily by postulating the intermediates **565** and **566**.

**Scheme 126:**
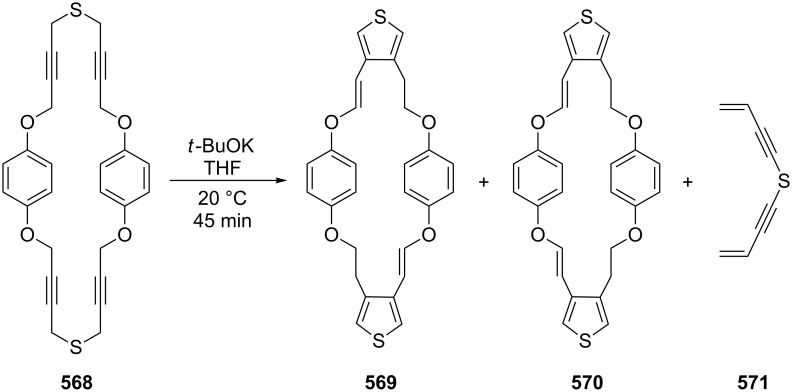
Preparation of *ansa*-compounds from macrocyclic bispropargyl thioethers.

The isomerization of the hydroquinone-derived bissulfide **568** is, however, more complex (Scheme 126) [291]. Here the bisthiophene derivatives **569** and **570** are the expected products, but the cleavage product **571** is also produced (**571**/**570**/**569** = 1:10:6). It results by cleaving off the hydroquinone dianion, which is a good leaving group.

**Scheme 127:**
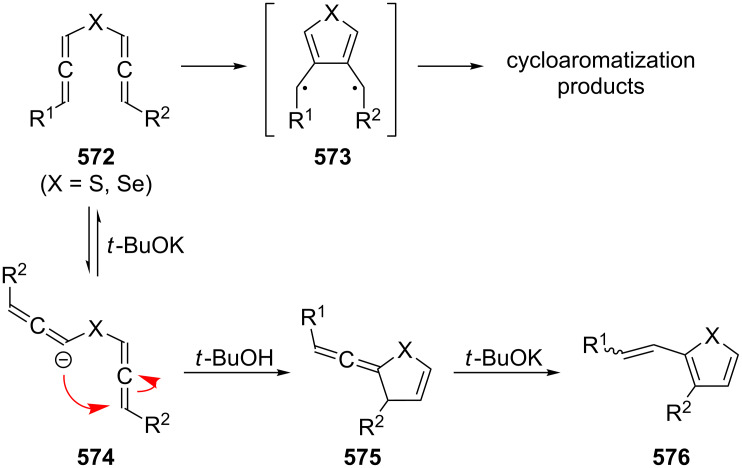
Alternate route for cyclization of a heterorganic bisallene.

The discussed diradical pathway is not the only route by which a dipropargyl (or a tetrapropargyl) sulfide (disulfide) or the corresponding selenide can react (Scheme 127) [292]. As displayed in Scheme 127 the heterobisallene **572** can react according to the “normal” path via diradical **573** to the diradical cycloaromatization products as discussed above several times. Alternatively, however, the base could abstract a (nonterminal) allene proton and generate the bisallenyl anion **574** as shown. If this attacks the other allene group and the resulting cycloallene carbanion is protonated by the solvent, then the semicyclic allene **575** results, which stabilizes itself to a vinyl thiophene/vinyl selenophene **576** under the influence of the base.

**Scheme 128:**
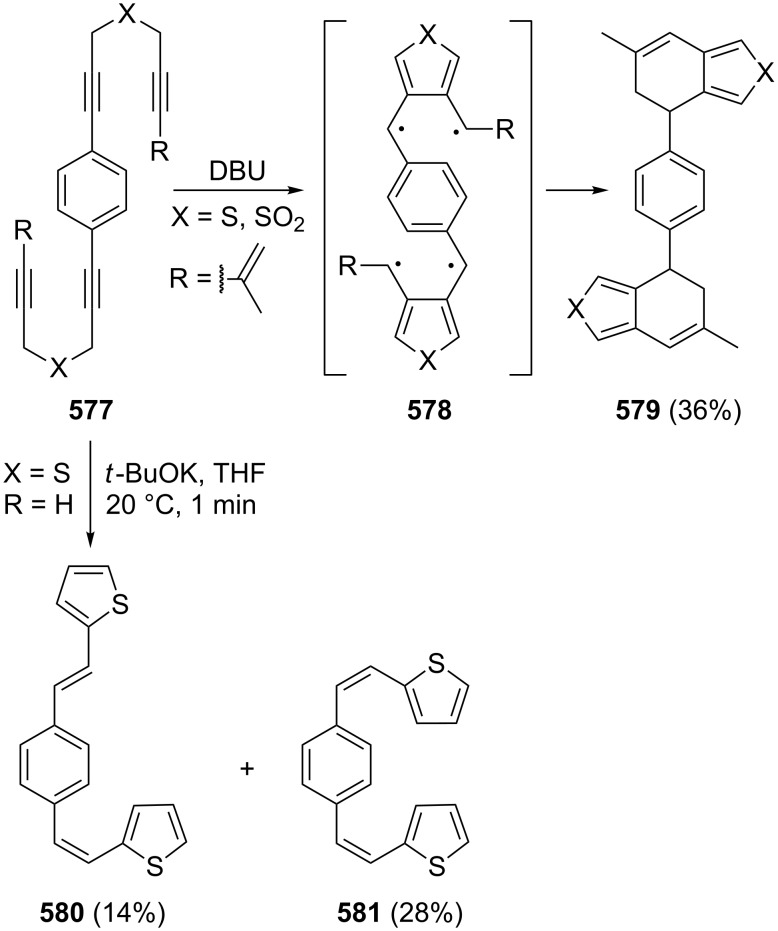
Multiple isomerization/cyclization of “double” bispropargylic thioethers.

One case in which such a competing reaction has been observed, concerns the “double” bispropargylic derivative **577** in Scheme 128. Here the usual diradical pathway leads to **579** via the intermediate species **578** (actually the process will most likely occur in two steps via a diradical rather than the “tetraradical” shown in **578**). The anionic cyclization pathway will lead to a mixture of the diastereomers **580** and **581** [292]. Surprisingly, no *E*,*E*-diastereomer of **580**/**581** was detected in the reaction mixture.

**Scheme 129:**
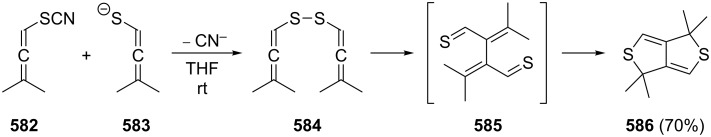
Preparation of a bisallenyl disulfide and its subsequent bicyclization.

Changing the bridging heteroatom to more complex molecular units opens up many new reaction pathways leading to a plethora of novel products. For brevity we only present some of these results in very condensed form, as obtained again largely by the Braverman group. For many of the mechanistic details of these processes the reader is referred to the original literature. The thienothiophene derivative **586** was produced in 70% yield when γ,γ-dimethylallenyl thiocyanate (**582**), was treated with lithium methoxide in THF at room temperature (Scheme 129) [293]. In this multistep transformation it is assumed that **582** is first cleaved to **583** and that these two components subsequently produce bis(γ,γ-dimethylallenyl) disulfide (**584**) as the decisive intermediate. The latter is then believed to undergo a consecutive [3.3] sigmatropic rearrangement to **585** followed by a double Michael addition to furnish the bicyclic product **586**.

**Scheme 130:**
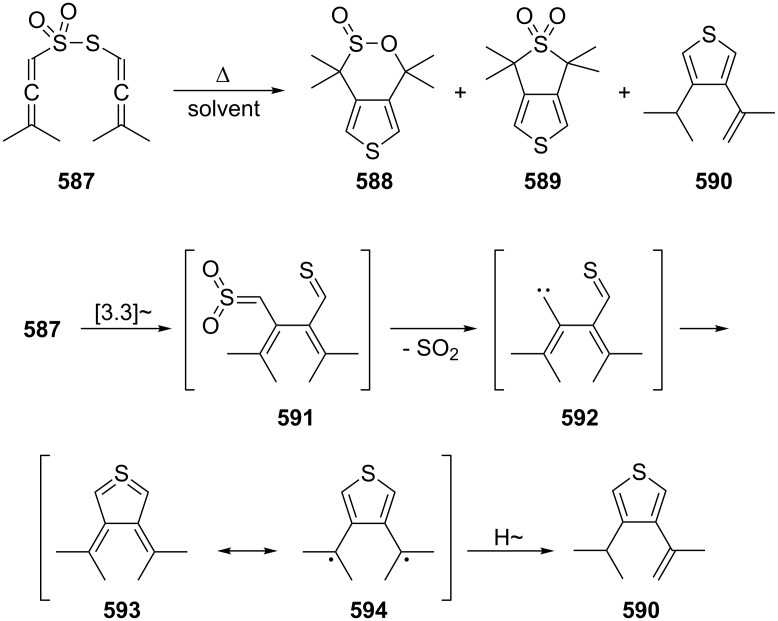
Thermal cyclization of a bisallenyl thiosulfonate.

On heating, bis(γ,γ-dimethylallenyl) thiosulfonate (**587**) undergoes a series of rearrangement and cyclization reactions to provide the three thiophene derivatives **588**–**590**. The transformation strongly depends on the nature of the solvent and the isomerization temperature. For example, in chloroform at 55 °C it yields the three products in 47:6:47 ratio in 50% total yield after 12 h, whereas in DMSO at 40 °C the yield increases to 80% after 12 h and only the first two products are obtained in 72:28 ratio (Scheme 130) [293–294]. Whereas **588** and **589** are presumably formed by an ionic mechanism (hence the solvent dependency of the reaction), the authors suggest a carbene–diradical sequence for the generation of the thiophene derivative **590** as summarized in Scheme 130 also. The isomerization begins with a [3.3] sigmatropic shift, which converts the substrate **587** into **591**, an intermediate that by sulfur dioxide extrusion is transformed into the carbene **592**. On cyclization of the latter, intermediate **593** is generated, which via its diradical resonance structure **594** is converted to the isolated product **590** by a hydrogen-transfer step.

**Scheme 131:**
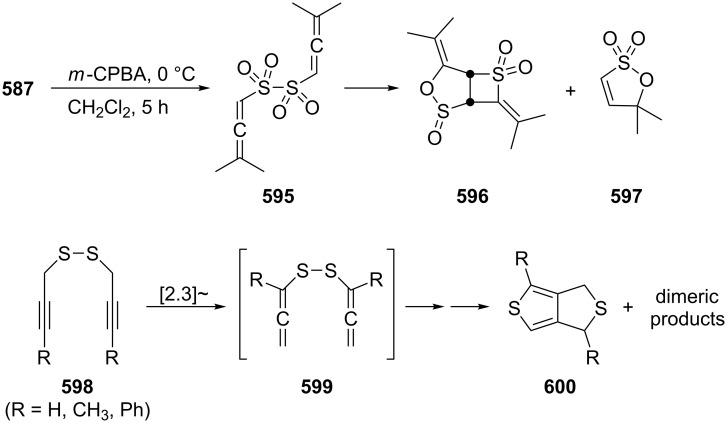
Some reactions of heteroorganic bisallenes with two sulfur atoms.

When **587** is oxidized with *meta*-chloroperbenzoic acid (*m*-CPBA) at 0 °C the bisallenic α-disulfone **595** is produced; it is much more reactive than its saturated counterpart and undergoes a multistep rearrangement to the highly functionalized bicyclic product **596** as well as a cleavage reaction of the S–S bond, which ultimately leads to the monocyclic product **597** (Scheme 131) [295]. The reduced form of **595**, i.e. intermediate **599**, can be accessed by a double [2.3] sigmatropic rearrangement of the dipropargylic disulfide **598**. This heteroorganic bisallene subsequently cyclizes to the bicyclic product **600**, among other things, in a multistep tandem process of high atom economy [296–297]. Note the structural similarity of product **586** in Scheme 129 with that of **600** in Scheme 131.

**Scheme 132:**
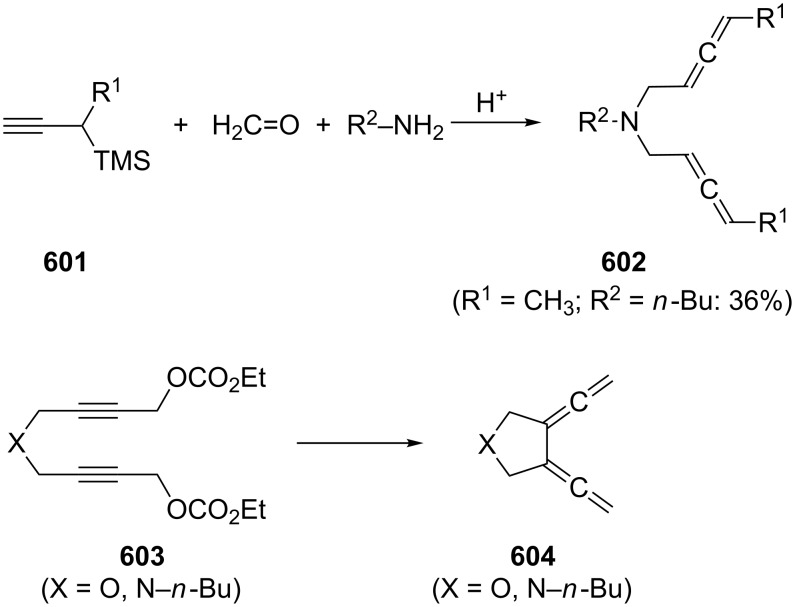
Further methods for the preparation of heteroorganic bisallenes.

Bisallenes containing nitrogen atoms in their spacers, i.e., bisallenic amines, have been obtained by various routes. Thus, Miginiac and co-workers prepared the bisallene **602** by an aminomethylation/desilylation process in which the propargylic substrate **601** is converted into the tertiary amine **602** (Scheme 132) [298–299]. The method reported by Sato [229] and by Hara et al. [231], mentioned already for all-carbon systems above (see Schemes 101 and 103), has also been employed for the preparation of heterocyclic semicyclic bisallenes, as demonstrated by the conversion of **603** into **604** [231].

#### 6.2 Transition metal-induced reactions of heteroorganic bisallenes

**Scheme 133:**
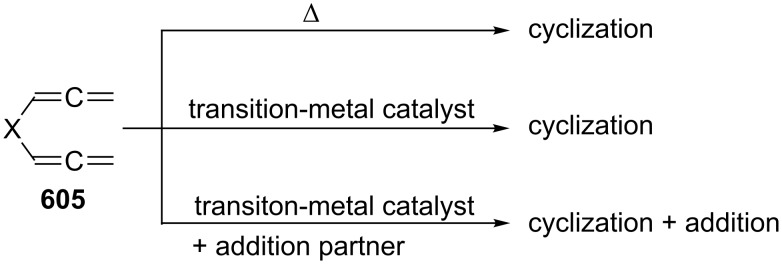
Cyclization reactions of heteroorganic bisallenes.

Principally, hetero bisallenes **605** can be employed in cyclization reactions in three ways (Scheme 133).

On heating without any addition of other reagents, they could undergo a [2 + 2] cycloaddition as described already in Section 6.1. This is, incidentally, the most characteristic reaction of allene itself. For the derivatives **605**, which are connected by a spacer –X– and hence less symmetric than allene, the cycloaddition could take place in head-to-head or head-to-tail fashion and involve either one of the two double bonds. One would therefore expect a complex mixture of [2 + 2] cycloadducts [300]. The second cyclization mode, taking place in the presence of a (transition) metal catalyst, could lead to a similar set of structural isomers. However, we would expect a greater selectivity here, as in many other metal-catalyzed (cyclo)additions. In the third case, the cyclization takes place in the presence of another component in stoichiometric amounts (carbon monoxide, simple amines, but also more complex organic compounds, see below). The prediction of the stereochemical selectivity is even more difficult in this case.

**Scheme 134:**
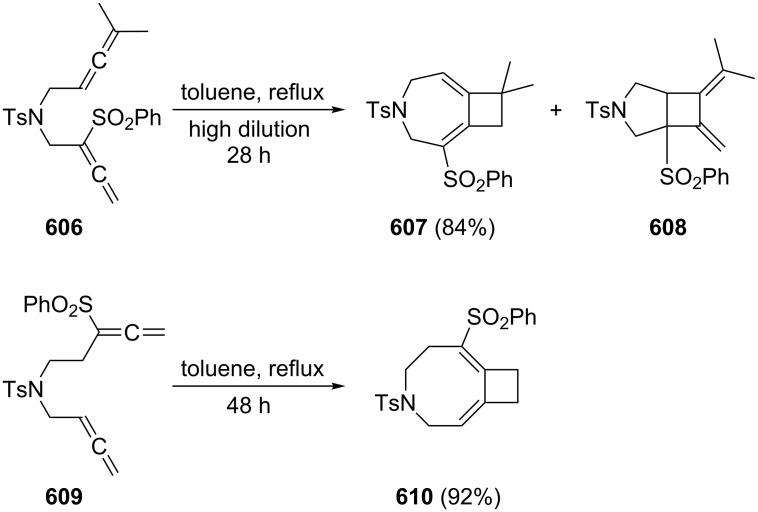
Thermal cycloadditions of bisallenic tertiary amines.

As it turns out, all types of cycloadditions of the heterorganic bisallenes have been reported. A typical example of the first reaction type has been described by Ma and co-workers (Scheme 134) [179]. Heating the 1,5-bisallene **606** provided the bicyclo[5.2.0]system **607** in very good yields. The product resulting from the reaction of the “inner” allene double bonds, i.e. the bicyclo[3.2.0]system **608**, was only produced as a minor cycloadduct (ratio **607**/**608** = 88.5:11.5). Obviously, both cycloadducts are head-to-head products. For the next higher homologue, the 1,6-bisallene **609**, the selectivity was even better with **610** formed exclusively in nearly quantitative yield [183].

**Scheme 135:**
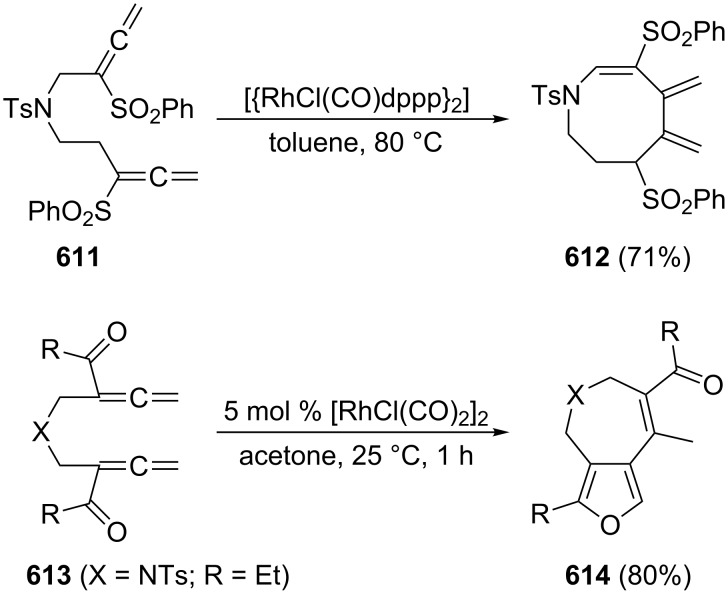
Cyclization of a bisallenic tertiary amine in the presence of a transition-metal catalyst.

Among the processes of the second category (reactions of heterorganic α,ω-bisallenes in the presence of a transition-metal catalyst) the following characteristic example by Mukai and co-workers may be cited (Scheme 135) [177]. Heating of the bissulfone diallene **611** in toluene solution in the presence of [{RhCl(CO)dppp}_2_] at 80 °C results in cyclization to the eight-membered ring compound **612** in good yield; the process is accompanied by the formation of a [2 + 2] cycloadduct and a Pauson–Khand product (see below), both generated in small amounts (10% each). Formally, the reaction constitutes an intramolecular ene reaction. The more likely mechanism involves the formation of a rhodacycle intermediate, which undergoes a 1,5-hydrogen shift. Reductive elimination under Rh(I) removal terminates the sequence [177]. If diketones **613**, rather than the above disulfones, are employed in this process, the reaction takes a different course as shown by Ma and co-workers [301]. Now, one of the carbonyl functions is incorporated into the ring system and the bridged furane derivative **614** is produced in very good yields. Replacing the amine function in **613** by oxygen furnishes a product **614** with X = O; this is, however, accompanied by small amounts of an isomer with a semicyclic double bond in the seven-membered ring, rather than an endocyclic one [181–182].

**Scheme 136:**
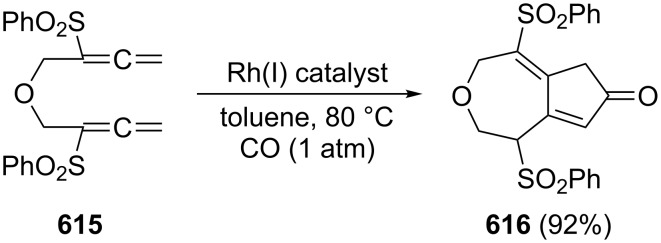
A Pauson–Khand reaction of a bisallenic ether.

More and more reports are appearing in the chemical literature in which an additional component is added in stoichiometric amounts to the catalyst/1,ω-bisallene mixture. In these cases the third component is incorporated into the resulting ring system and complex bicyclic products carrying useful functional groups are generated. A case in point is provided by various Pauson–Khand reactions starting from heteroorganic bisallenes. For example, Mukai and co-workers have shown that the bisallene ether **615** in the presence of rhodium catalysts and carbon monoxide yields the bicyclic ketone **616** in excellent yields (Scheme 136) [177]. The activating PhSO_2_-groups can be removed from the ring system by treatment with excess tri-*n*-butyltin hydride and higher homologues of, e.g., **616** have also been prepared. The mechanism proposed for the cyclization involves the formation of rhodacyclic intermediates.

**Scheme 137:**
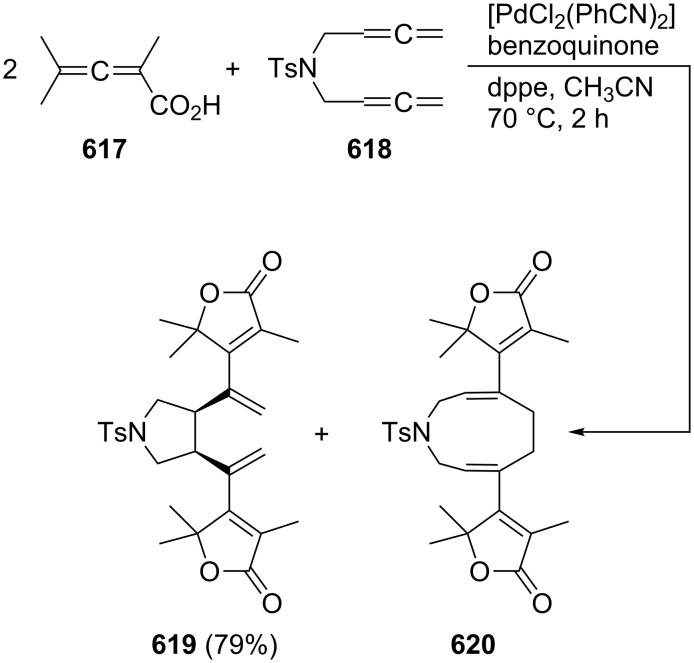
Formation of a 2:1adduct from two allenic substrates.

A sandwich-type triple cyclization of two equivalents of the 2,3-allenoic acid **617** and the bisallenic amine **618** takes place when a mixture of these components is treated with various Pd-complexes to yield the *cis*-configured 2:1 adducts **619** in medium to good yields (30–80%); as a side product **620** is produced in up to 20% yield (Scheme 137) [178]. The all-carbon analogue of this case has already been discussed above (see Scheme 74).

**Scheme 138:**
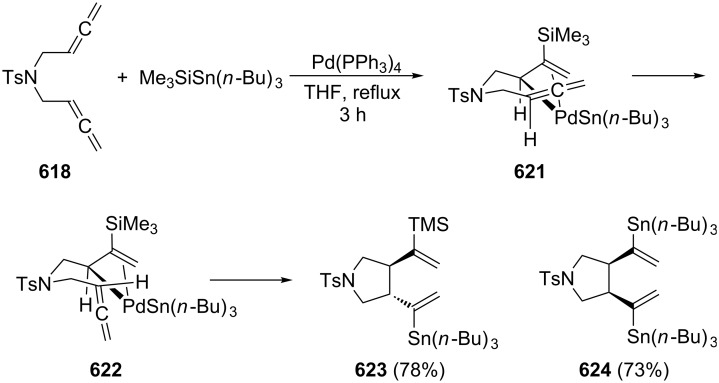
A ring-forming silastannylation of a bisallenic tertiary amine.

A Pd-catalyzed carbocyclization of **618** by way of a silastannylation has been reported by Kang and co-workers (Scheme 138) [186–187]. Thus, when the tertiary amine **618** is heated in THF under reflux in the presence of (trimethylsilyl)tri-*n*-butylstannane and a Pd catalyst, it cyclizes in good yield to the *trans*-configured five-membered ring system **623**. It is assumed that a Me_3_SiPdSn(*n*-Bu)_3_ species adds to one of the allene groups of **618** in the first step to produce a σ- or π-allylpalladium complex **621**. Because of steric hindrance this adopts another conformation, **622**, which fulfills the necessary conditions to cyclize to **623**. When the trimethylsilylstannane is replaced by (*n*-Bu)_3_Sn–Sn(*n*-Bu)_3_, a cyclization reaction occurs with **618** too, but it leads to the *cis*-configured product **624**; steric reasons are held responsible for this stereoselectivity.

**Scheme 139:**
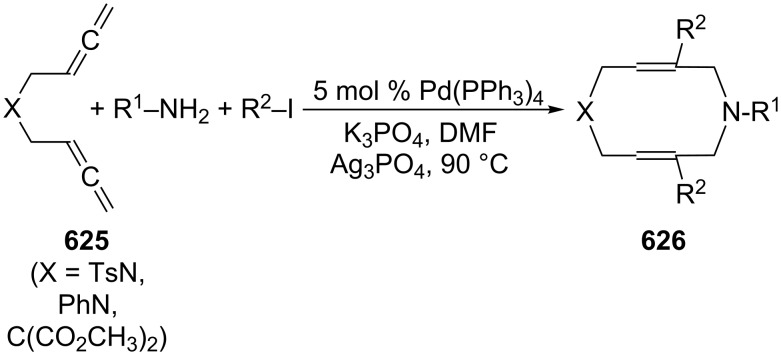
A three-component cyclization involving a heterorganic bisallene.

That these cyclizations can be developed into three-component routes yielding, e.g., a functionalized ten-membered ring was demonstrated by Ma and co-workers (Scheme 139) [184]. Bisallenes of the general type **625** afforded the cyclodecadiene derivatives **626** in moderate to good yields (40–60%) when reacted with organic halides and primary amines in the presence of Pd(0) catalysts. The products **626** are produced with high chemo- and stereoselectivity, and a mechanism involving two π-allylic palladium intermediates has been proposed to account for this result.

**Scheme 140:**
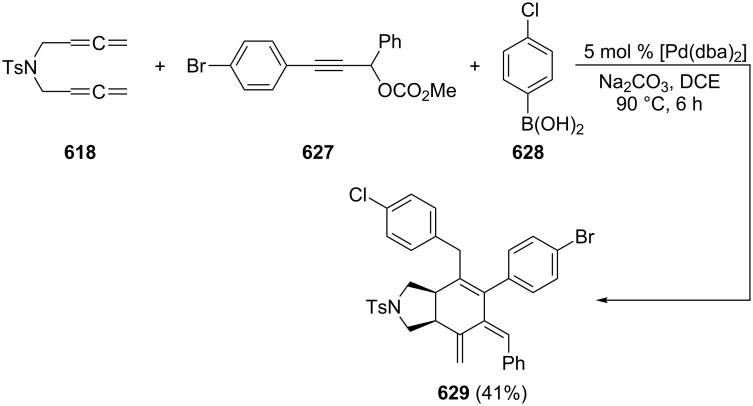
Atom-economic construction of a complex organic framework from a heterorganic α,ω-bisallene.

We close this review with still another coupling/cyclization reaction in which different propargylic carbonates, phenylboronic acids and heterorganic α,ω-bisallenes are reacted with each other in the presence of [Pd(dba)_2_]. A typical example is shown in Scheme 140 [180]. In this case the bromocarbonate **627** is coupled with 4-chlorophenylboronic acid (**628**) and the bisallene **618** to yield the adduct **629** in 41% yield. The product possesses a *cis*-junction between its five- and six-membered rings and is produced as the single diastereomer shown. The yields of this remarkable process vary between 40 and 80%.

## Conclusion

Beginning with the simplest possible bisallene, 1,2,4,5-hexatetraene (**2**), one of the isomers of benzene, we have seen how this class of compounds has rapidly gained importance in structural and synthetic organic chemistry during the past decades. Especially the past few years have witnessed the development of many new approaches to complex organic target molecules by a relatively small number of steps. We assume that this trend will remain strong, since allenes and bisallenes, as well as other highly unsaturated π-systems, with their high concentration of π-electrons on a relatively small number of carbon atoms are very attractive substrates for the rapid and efficient generation of molecular complexity with excellent atom economy.

Needless to say, there are a few neglected areas in bisallene chemistry. One concerns the systematic study of organometallic compounds possessing more than one allene moiety, whether these are complexed bis (or tris and oligo) allenes or covalently bonded derivatives. Bis(allenyl)mercury seems to be the only organometallic compound so far in which the allene groups are bonded by covalent bonds to a metal atom [302–303]. Another neglected area concerns the photochemistry of bisallenes. The photochemistry of allenes as a whole has so far not received the attention it clearly deserves [304–307]. It is likely that novel phototransformations, preferentially photoadditions and photoisomerizations, will be discovered on closer inspection and with a larger variety of allenes than studied so far. Furthermore, the number of bisallenic natural products that have been described so far is very small. In this area one can assume that more discoveries will be made once a directed search is initiated [308]. The occasional use of bisallenes in polymer chemistry has been pointed out in this review (see Section 4.1). More results should be forthcoming in this field with compounds other than the mentioned diallenes serving as monomers.

As far as the synthesis of bisallenes is concerned we predict that the classical routes to these compounds, i.e. base-catalyzed or thermal isomerization reactions of acetylenes (the DMS-approach), will slowly fade out and will be replaced by modern, largely metal-induced coupling reactions. These reactions often take place under mild conditions, which is an important prerequisite for the preparation of highly reactive derivatives; are in many cases more specific than the classical routes; produce a smaller number of side products; and should allow the introduction of additional, preparatively useful functional groups (especially carbonyl functions).

In addition, looking at the many novel and often highly imaginative recent developments in the field of highly unsaturated compounds [309] makes one optimistic about the future of di- and oligoallene chemistry.
